# Apigenin Combined with Aerobic Exercise Is Associated with Attenuated Renal Oxidative Stress and AMPK/SIRT1/PGC-1α Pathway Activation in NAFLD Mice

**DOI:** 10.3390/cimb48090942

**Published:** 2026-09-15

**Authors:** Yu Cheng, Lina Peng, Kunda Yang, Nixi Huang, Yu Wang, Yewenqian Zhang, Yunshuang Liu, Chuangye Liu, Lili Sun

**Affiliations:** 1 Graduate School, Harbin Sport University, Harbin 150008, China; 2 School of Kinesiology and Health, Harbin Sport University, Harbin 150008, China

**Keywords:** apigenin, aerobic exercise, NAFLD, renal injury, oxidative stress, AMPK/SIRT1/PGC-1α pathway

## Abstract

Non-alcoholic fatty liver disease (NAFLD) induces renal injury via the liver–kidney axis, with oxidative stress as the core pathological mechanism. Apigenin (API) and aerobic exercise (AE) exhibit antioxidant and metabolic regulatory properties, but their combined renoprotective effect in NAFLD and the underlying mechanism remain poorly characterized. This study aimed to explore the protective efficacy of API combined with AE against NAFLD-associated renal damage and its association with the AMP-activated protein kinase (AMPK)/sirtuin 1 (SIRT1)/peroxisome proliferator-activated receptor gamma coactivator 1-alpha (PGC-1α) pathway. A NAFLD mouse model was established via 12-week high-fat-diet feeding, followed by 8-week API intraperitoneal injection and/or treadmill AE intervention. Renal histology, biochemical parameters, inflammatory cytokines, tubular injury markers, and pathway molecules were examined. Combined intervention significantly reduced serum creatinine (SCr) and blood urea nitrogen (BUN), alleviated renal lipid accumulation, and attenuated oxidative stress and inflammation. It significantly downregulated the tubular injury marker KIM-1, and induced a downward trend in NGAL and L-FABP without statistical significance. The combined intervention also enhanced AMPK phosphorylation and upregulated SIRT1 and PGC-1α protein expression, with greater effects than single interventions and significant statistical interactions between the two interventions on these pathway molecules. These results suggest that API combined with AE attenuates NAFLD-related renal injury, and that this protective effect is associated with the upregulation of AMPK phosphorylation and SIRT1/PGC-1α protein expression, the significant statistical interaction between the two interventions suggests the potential involvement of this pathway in mediating renoprotection. These findings provide preliminary evidence for the association between the AMPK/SIRT1/PGC-1α pathway and the renoprotective effect of this combined intervention strategy.

## 1. Introduction

With global shifts in dietary patterns and lifestyle, non-alcoholic fatty liver disease (NAFLD) has become the most prevalent chronic liver disease worldwide, affecting approximately 25% of the global population [1]. In China, the prevalence of NAFLD has reached 20.09% and continues to rise in younger populations [1]. Beyond hepatic injury itself, NAFLD significantly increases the risk of extrahepatic complications including type 2 diabetes mellitus, cardiovascular disease, and chronic kidney disease (CKD). Among these, NAFLD-associated renal injury has emerged as a non-negligible public health concern [2]. Clinical evidence demonstrates that the prevalence of CKD is markedly higher in patients with NAFLD than in non-NAFLD populations, and the severity of NAFLD is positively correlated with the incidence and progression of CKD [3]. Under NAFLD conditions, redox imbalance driven by dysregulated lipid metabolism serves as the core pathological mechanism underlying renal injury. As a mitochondria-rich organ with high metabolic activity, the kidney is highly susceptible to oxidative damage [4]. Excessive production of reactive oxygen species (ROSs) and impaired antioxidant defense systems directly damage glomerular and tubular epithelial cells, while concurrently exacerbating inflammatory responses and mitochondrial dysfunction, collectively driving renal injury progression [5]. To date, no specific pharmacotherapies have been approved for NAFLD. Given the substantial medical and health burden imposed by this condition, exploring safe and effective intervention strategies has become a clinical priority [6].

Apigenin (API) is a natural flavonoid widely present in vegetables, fruits, and legumes, with verified biological activities including glycolipid metabolism regulation [7], antioxidant capacity [8], anti-inflammatory effects [9,10,11], and hepatorenal protection [12]. The phenolic hydroxyl groups in its molecular structure form the structural basis for its antioxidant activity [9]. The existing studies indicate that apigenin alleviates renal oxidative stress injury by directly scavenging free radicals and regulating antioxidant signaling pathways [9], and modulates the AMPK/SIRT1/PGC-1α pathway to promote mitochondrial biogenesis and improve cellular energy metabolism [13]. Aerobic exercise (AE) is a first-line non-pharmacological intervention for NAFLD, which improves circulating lipid profiles by enhancing whole-body fat oxidation and energy expenditure [14,15]. The redox regulation effect of aerobic exercise follows a dose-dependent pattern: strenuous exercise induces a transient elevation in ROS levels, whereas regular moderate-intensity aerobic exercise sustainably upregulates endogenous antioxidant defense capacity and improves cellular redox status [16]. It also activates the AMPK/SIRT1/PGC-1α pathway [17] to strengthen renal antioxidant defense and reduce the accumulation of lipid peroxidation products such as malondialdehyde (MDA) and 4-hydroxynonenal (4-HNE). Although single interventions with apigenin or aerobic exercise show promising renoprotective potential, the existing studies predominantly focus on monotherapy effects, and NAFLD-related research largely centers on hepatic lesions. The regulatory mechanism of renal oxidative stress in NAFLD-associated renal injury remains insufficiently characterized, and whether the combination of the two exerts greater renoprotective effects than either monotherapy, and whether there is a statistical interaction between the two interventions in regulating the AMPK/SIRT1/PGC-1α pathway, has yet to be determined.

AMP-activated protein kinase (AMPK) is a core cellular energy sensor that governs systemic lipid and energy homeostasis in metabolic organs, including the kidney [18]. Upon activation, AMPK inhibits lipogenesis and promotes fatty acid oxidation, making it a key therapeutic target for metabolic diseases [19,20]. Silent information regulator 1 (SIRT1), an NAD+-dependent deacetylase, is widely expressed in renal podocytes and tubular epithelial cells [21]. It suppresses the transcription of pro-inflammatory cytokines such as tumor necrosis factor-α (TNF-α) and interleukin-6 (IL-6), and upregulates antioxidant enzymes including superoxide dismutase (SOD), catalase (CAT), and glutathione peroxidase (GPx). Excessive lipid accumulation impairs AMPK signaling and concurrently inhibits SIRT1 activity, forming a positive feedback loop that exacerbates metabolic dysregulation [22]. Peroxisome proliferator-activated receptor gamma coactivator 1-alpha (PGC-1α) is a master regulator of mitochondrial biogenesis and energy metabolism that is highly expressed in high-energy-demand organs such as the kidney and liver, and it acts as a critical modulator of the endogenous antioxidant system [23]. PGC-1α directly enhances cellular defense capacity by promoting antioxidant enzyme expression [24], and alleviates oxidative damage by scavenging excess ROSs and maintaining mitochondrial function [25]. Its activity is tightly regulated by phosphorylation and acetylation; SIRT1-mediated deacetylation is a key step for PGC-1α activation and oxidative stress clearance [26]. Collectively, the AMPK/SIRT1/PGC-1α cascade forms a core signaling network integrating energy metabolism, mitochondrial function, and antioxidant defense, and its suppression contributes to exacerbated renal oxidative stress in NAFLD mice [27].

Despite accumulating evidence that apigenin or aerobic exercise alone can activate the AMPK/SIRT1/PGC-1α axis and mitigate metabolic organ damage, three critical knowledge gaps persist regarding NAFLD-associated renal injury: whether the combined regimen produces renoprotective effects exceeding those of either single intervention; whether this effect is accompanied by coordinated upregulation of the renal AMPK/SIRT1/PGC-1α cascade; and whether upregulation of these key pathway molecules correlates with the attenuation of downstream lipotoxicity-driven oxidative stress and inflammation.

To address these gaps, we hypothesize that combined apigenin and aerobic exercise exerts non-additive renoprotective effects relative to either single intervention (a statistical interaction pattern), and that this benefit is associated with activation of the renal AMPK/SIRT1/PGC-1α signaling axis, a concurrent reduction in ectopic lipid deposition, decreased ROS levels, and alleviation of the vicious cycle between oxidative stress and inflammation, which together correspond to improved tubular injury and functional impairment in NAFLD mice.

Accordingly, this study was designed to (1) evaluate the ameliorative effects of the combined intervention (API + AE) on renal function, histology, lipid metabolism, oxidative stress, inflammation, and tubular injury in a high-fat-diet-induced NAFLD mouse model; (2) examine the statistical interaction between the two interventions via two-way ANOVA; and (3) analyze the expression profiles of the AMPK/SIRT1/PGC-1α pathway and its downstream antioxidant targets in response to the combined regimen. Our findings provide correlational evidence that the enhanced renoprotection afforded by the combined intervention is associated with coordinated upregulation of the AMPK/SIRT1/PGC-1α pathway, and lay a foundation for future mechanistic dissection using pathway-specific inhibitors or genetic models.

## 2. Materials and Methods

### 2.1. Experimental Animals and NAFLD Model Establishment

Fifty-two male C57BL/6 mice aged 6–8 weeks of specific pathogen-free (SPF) grade were purchased from Liaoning Changsheng Biotechnology Co., Ltd. (Benxi, China), with the animal production license number 210726240102645116. All animal experimental procedures were performed in strict accordance with the ARRIVE guidelines and the requirements for laboratory animal welfare and ethics, and the protocol was approved by the Laboratory Animal Ethics Committee of Harbin Sport University (Ethics Approval No. 2025010). Mice were housed in a barrier maintenance system at Harbin Veterinary Research Institute under controlled conditions (22 ± 2 °C, 12 h light/12 h dark cycle, 50–60% relative humidity), with 4–5 mice per cage and free access to food and water throughout the experiment. After a 1-week acclimatization period, the 52 mice were randomly divided into two groups based on body weight: the blank control group (CON group, *n* = 10) and the NAFLD model group (HFD group, *n* = 42). Mice in the CON group were fed a standard maintenance diet with no additional intervention, while mice in the model group were fed a high-fat diet to induce NAFLD. The high-fat diet used for modeling was the D12492 formula, with 60% of total calories derived from fat. Mice in the model group first underwent a 1-week gradient acclimation with progressively increasing dietary fat content, followed by 12 weeks of ad libitum access to the full high-fat diet (experimental weeks 2 to 13).

The initial sample sizes were designated with allowance for model validation animals, and were determined based on conventions in similar NAFLD mouse model studies, which have consistently reported sufficient statistical power for detecting differences in renal function and oxidative stress parameters with 6–8 mice per group. Mice that died spontaneously or developed overt signs of illness (e.g., severe infection, tumors) during the experimental period were excluded from the analysis. No mice met these exclusion criteria.

At the end of week 13 (after 12 weeks of high-fat feeding), 3 mice were randomly selected from each of the CON and model groups for model validation. After euthanasia by cervical dislocation under deep anesthesia (detailed in Section 2.4), liver tissues were dissected, fixed, and prepared into paraffin sections for hematoxylin and eosin (H&E) staining. Histological evaluation was performed by two independent researchers blinded to grouping. Mice in the model group showing diffuse macrovesicular steatosis in hepatocytes were considered to have established the NAFLD model successfully.

After successful modeling, the remaining 7 mice in the CON group continued normal feeding until the end of the experiment. The remaining 39 mice in the model group were randomly divided into 5 subgroups based on body weight: high-fat-diet group (HFD group, *n* = 8), high-fat-diet + solvent control group (SO group, *n* = 8), high-fat-diet + aerobic exercise monotherapy group (HFD + AE group, *n* = 8), high-fat-diet + apigenin monotherapy group (HFD + API group, *n* = 8), and high-fat-diet + aerobic exercise combined with apigenin group (HFD + AE + API group, *n* = 7). All subgroups continued to receive high-fat-diet feeding until the end of the experiment.

### 2.2. AE Intervention Protocol

The exercise protocol was established with reference to the classic moderate-intensity treadmill training scheme for NAFLD mice [28], with adjustments based on the actual exercise capacity of the animals. This protocol corresponds to approximately 65% of maximal oxygen uptake (VO_2_max) in mice, which is defined as moderate-intensity aerobic exercise. Mice in the HFD + AE and HFD + AE + API groups performed aerobic exercise on a treadmill with 0° incline. During experimental week 14, a 1-week adaptive training phase was conducted: mice started running at 10 m/min for 20 min on the first day, with daily increments of 10 min in duration and 1 m/min in speed until reaching 14 m/min for 60 min per session.

Formal training was initiated at experimental week 15 and continued for 8 consecutive weeks (weeks 15 to 22), with 5 training sessions per week. Mice in the non-exercise groups were placed on a non-moving treadmill for the same frequency and duration to control for environmental factors and handling stress. During training, mice were gently prodded with a soft brush when necessary to maintain running; no electrical shock was used. All mice were euthanized and sampled 48 h after the last training session to eliminate the acute effect of the final exercise bout.

### 2.3. API Administration Protocol

API (purity ≥ 98%, purchased from Shanxi Yuning Biotechnology Co., Ltd., Taiyuan, China) is a natural flavonoid with potent antioxidant activity. The administration dose of 50 mg/kg/d was set with reference to previous preclinical studies on renal protection [29,30]. Briefly, API powder was first dissolved in 100% dimethyl sulfoxide (DMSO) to prepare a stock solution. Before use, the solution was diluted to working concentration with sterile 0.9% normal saline, ensuring a final DMSO concentration of 1% in the injection solution. This concentration of DMSO has been verified to have no nephrotoxicity in mice [31].

API was administered via intraperitoneal (i.p.) injection at a dose of 50 mg/kg/d. The injection volume was adjusted according to individual body weight (10 mL/kg), and did not exceed 0.5 mL per mouse. All solutions were prepared fresh immediately before use. From experimental week 15, mice in the HFD + API and HFD + AE + API groups received daily intraperitoneal injection of apigenin for 8 consecutive weeks (weeks 15 to 22). Administration was performed at a fixed time every morning, and on exercise days, injection was given 1 h before treadmill training. Mice in the SO group received an equal volume of 1% DMSO-normal saline solvent via intraperitoneal injection over the same period. The SO group was included to control for any potential effects of the DMSO vehicle on the measured renal parameters.

### 2.4. Tissue and Serum Sample Collection

At the end of experimental week 22, all mice were fasted overnight (12 h, with free access to water). After anesthesia via intraperitoneal injection of ketamine (80 mg/kg) and xylazine (10 mg/kg), terminal blood was collected by retro-orbital sinus puncture and subsequent enucleation to obtain sufficient volume. Immediately thereafter, mice were euthanized by cervical dislocation, in full compliance with the AVMA Guidelines for the Euthanasia of Animals.

Whole blood samples were allowed to stand at room temperature for 1 h, then centrifuged at 4500 rpm for 10 min at 4 °C. The upper serum layer was aliquoted into RNase/DNase-free cryotubes and stored at −80 °C for subsequent biochemical detection.

Following euthanasia, bilateral kidneys were rapidly dissected and rinsed with ice-cold normal saline to remove residual blood, and processed independently for different assays. The right kidney was trimmed to retain representative renal tissue blocks from the same anatomical site, fixed in 4% paraformaldehyde for paraffin embedding and section preparation (*n* = 3 per group, for histological and immunohistochemical analyses). The left kidney was snap-frozen in liquid nitrogen and transferred to −80 °C storage for subsequent biochemical and molecular biological detection. Tissue homogenates prepared from the left kidneys were used for biochemical assays and ELISA (*n* = 5 per group); aliquots from the same homogenate batches were used for Western blotting and quantitative real-time PCR (qPCR) (*n* = 3 per group). All sample subsets were randomly selected from the full animal cohort of each experimental group.

### 2.5. Renal Histological Examination

For paraffin section preparation, renal tissues were fully fixed in 4% paraformaldehyde fixative for 24 h, dehydrated in a graded ethanol series, cleared with xylene, infiltrated with paraffin, and embedded in paraffin wax. Sections with a thickness of 5 μm were prepared using a rotary microtome.

For H&E staining, paraffin sections were dewaxed with xylene and rehydrated using graded ethanol, then stained with hematoxylin for 5 min, differentiated with 1% hydrochloric acid ethanol, rinsed in tap water, and counterstained with eosin for 2 min. The sections were then dehydrated with graded ethanol, cleared with xylene, and mounted with neutral balsam. For periodic acid–Schiff (PAS) staining, dewaxed and rehydrated paraffin sections were oxidized in 0.5% periodic acid solution for 10 min at room temperature. After thorough rinsing with distilled water, sections were incubated with Schiff reagent in the dark for 15 min. The sections were then counterstained with hematoxylin for 2 min, differentiated with acidic ethanol, and blued in running tap water. Following dehydration through a graded ethanol series and clearing with xylene, sections were mounted with neutral balsam.

All histological evaluations were performed by two independent pathologists blinded to the experimental groups. To quantify glomerular mesangial expansion, the mesangial matrix area fraction was measured on PAS-stained sections. Briefly, 10–15 glomeruli per mouse (*n* = 3 per group) were randomly captured at ×400 magnification. Using Fiji (ImageJ 1.54f) software, the glomerular tuft area was manually outlined, and the PAS-positive mesangial matrix area was segmented using a fixed color threshold. The mesangial matrix area fraction was calculated as (PAS-positive area/total glomerular tuft area) × 100%, and averaged per animal.

All histological analyses were performed on 3 randomly selected mice per group (*n* = 3 biological replicates). The sample size for histological scoring (*n* = 3 per group) was chosen based on previous similar studies and was considered adequate for semi-quantitative lesion scoring with consistent effect sizes.

### 2.6. Detection of Renal Function and Lipid Parameters

Serum samples were collected as described in Section 2.4. Serum creatinine (SCr, Cat. No. C011-2-1) and blood urea nitrogen (BUN, Cat. No. C013-3-1) levels were measured using commercial assay kits (Nanjing Jiancheng Bioengineering Institute, Nanjing, China) to evaluate renal function.

Renal cortical tissues were accurately weighed and homogenized in ice-cold normal saline to prepare 10% (*w*/*v*) homogenates. After centrifugation at 12,000× *g* for 10 min at 4 °C, the supernatants were collected. The contents of triglyceride (TG, Cat. No. A110-1-1), total cholesterol (TC, Cat. No. A111-1-1), and free fatty acid (FFA, Cat. No. A042-2-2) in renal tissue were measured using corresponding kits from the same manufacturer.

All procedures were performed strictly according to the kit instructions. Five randomly selected mice per group were used for all assays in this section (*n* = 5 biological replicates).

### 2.7. Detection of Renal Oxidative Stress and Inflammatory Markers

Renal tissue homogenates (10%, *w*/*v*) were prepared as described in Section 2.6. Biochemical assay kits from Nanjing Jiancheng Bioengineering Institute were used to detect the enzymatic activities of SOD (Cat. No. A001-3-2) and CAT (Cat. No. A007-1-1), as well as the contents of reduced GSH (Cat. No. A006-2-1), MDA (Cat. No. A003-1-2), and 8-hydroxy-2′-deoxyguanosine (8-OHdG, Cat. No. H165-1-1) in renal tissue. All procedures were performed strictly according to the kit instructions.

The protein contents of TNF-α (Cat. No. MM-0132M2) and IL-6 (Cat. No. MM-1011M2) in renal tissue were detected using enzyme-linked immunosorbent assay (ELISA) kits purchased from Jiangsu Meimian Industrial Co., Ltd. (Yancheng, China). All procedures were performed strictly in accordance with the manufacturer’s instructions.

All oxidative stress biochemical assays and ELISA tests were performed on 5 randomly selected mice per group (*n* = 5 biological replicates).

### 2.8. Quantitative Real-Time PCR (qRT-PCR) Assay

Renal tissue was minced and placed in a centrifuge tube containing TRNzol universal total RNA extraction reagent. Total RNA was extracted according to the reagent manufacturer’s protocol. RNA purity and integrity were verified by ultraviolet spectrophotometry (OD260/280 = 1.8–2.0, OD260/230 ≥ 1.8) and agarose gel electrophoresis, respectively. Using matching reverse transcription reagents, the total RNA was reverse-transcribed into cDNA on a PCR instrument.

Using cDNA as the template, amplification was performed on a LightCycler^®^ 96 real-time fluorescence quantitative PCR system (Roche, Basel, Switzerland) with SYBR Green premix. The reaction conditions were as follows: pre-denaturation at 95 °C for 30 s; 40 cycles of denaturation at 95 °C for 5 s; annealing at 60 °C for 30 s; and extension at 72 °C for 30 s. Melting curve analysis was performed after amplification to confirm the specificity of PCR products.

Mouse *Gapdh* was used as the internal reference gene, and its stable expression under high-fat-diet conditions was verified in pre-experiments. The relative mRNA expression levels of target genes were calculated using the 2^−ΔΔCt^ method. The primer sequences are listed in Table 1. The target genes detected included *Prkaa1* (AMPKα1), *Sirt1*, *Ppargc1a* (PGC-1α), *Sod1*, *Cat*, *Gpx1*, *Tnf* (TNF-α), *Il6* (IL-6), *Havcr1* (KIM-1), *Lcn2* (NGAL), and *Fabp1* (L-FABP).

For qRT-PCR analysis, 3 mice were randomly selected from each group for detection (*n* = 3 biological replicates).

### 2.9. Western Blot Analysis

An appropriate amount of renal tissue was added to RIPA lysis buffer containing protease and phosphatase inhibitors, fully ground on ice to prepare homogenate, and lysed on ice for 30 min. The mixture was then centrifuged at 12,000× *g* for 15 min at 4 °C, and the supernatant (total protein extract) was collected. The bicinchoninic acid (BCA) protein assay was used to detect the total protein concentration in the extracts, and the protein concentration of each sample was normalized to the same level based on the detection results. Normalized protein samples (5 μg total protein per lane) were loaded into the wells of 10–12% sodium dodecyl sulfate–polyacrylamide gel electrophoresis (SDS-PAGE) gels. Electrophoresis was performed at 80 V for 30 min followed by 120 V for 90 min. After electrophoretic separation, proteins in the gel were transferred onto polyvinylidene fluoride (PVDF) membranes at 300 mA for 90 min on ice. After transfer, the membranes were blocked with 5% skim milk in Tris-buffered saline with Tween 20 (TBST) at room temperature for 1 h to avoid non-specific binding.

Primary antibodies used for immunoblotting were as follows: phosphorylated AMP-activated protein kinase (p-AMPK, Cat. No. M00994-3, Boster Biological Technology, Wuhan, China); total AMPK (Cat. No. A00994-6, Boster Biological Technology, Wuhan, China); SIRT1 (Cat. No. PB0173, Boster Biological Technology, Wuhan, China); PGC-1α (Cat. No. T56630, Abmart, Shanghai, China); superoxide dismutase 1 (SOD1, Cat. No. PB0453, Boster Biological Technology, Wuhan, China); kidney injury molecule-1 (KIM-1, Cat. No. bs-2713R, Bioss, Beijing, China); neutrophil gelatinase-associated lipocalin (NGAL, Cat. No. PB9609, Boster Biological Technology, Wuhan, China); and liver-type fatty acid-binding protein (L-FABP, Cat. No. bsm-60842R, Bioss, Beijing, China). All primary antibodies were diluted at 1:1000 and incubated with membranes overnight at 4 °C. β-actin (Cat. No. BA2305, Boster Biological Technology, Wuhan, China) was used as the loading control for total protein normalization, except for p-AMPK, which was normalized to total AMPK. After washing, the membranes were incubated with horseradish peroxidase (HRP)-conjugated goat anti-rabbit IgG H&L secondary antibody (1:20,000, Cat. No. 511203, ZEN BIO, Chengdu, China) for 1 h at room temperature. After another wash with the TBST buffer, an enhanced chemiluminescence (ECL) substrate (Cat. No. MA0186-1, Meilunbio, Dalian, China) was added, and protein band signals were recorded using a chemiluminescence imaging system. Finally, Fiji (ImageJ 1.54f) software was used for objective quantitative analysis of band gray values. For Western blot analysis, 3 mice were randomly selected from each group for detection (*n* = 3 biological replicates).

### 2.10. Immunohistochemical Staining

Immunohistochemical (IHC) staining was performed on paraffin sections fixed with 4% paraformaldehyde. Briefly, paraffin sections were dewaxed with xylene and rehydrated through graded ethanol, then rinsed with distilled water. Microwave heat-induced antigen retrieval was performed for 15 min using sodium citrate antigen retrieval solution (pH 6.0), and sections were allowed to cool naturally to room temperature.

After washing 3 times with phosphate-buffered saline (PBS, pH 7.4), sections were blocked with 5% bovine serum albumin (BSA) at room temperature for 30 min and incubated with primary antibodies overnight at 4 °C. After 3 washes with PBS (pH 7.4), sections were incubated with the same HRP-labeled goat anti-rabbit IgG secondary antibody used for Western blotting (see Section 2.9) at room temperature for 30 min. After 3 additional washes with PBS, 3,3′-diaminobenzidine (DAB) chromogenic substrate was added for immunohistochemical color development, with reaction time controlled under microscopic observation to avoid over-staining, followed by hematoxylin counterstaining of cell nuclei. Sections were then dehydrated with graded ethanol, cleared with xylene, and mounted with neutral balsam.

Stained tissues were observed under a microscope (Sunny RX50, Sunny Optical, Ningbo, China), and images were captured for subsequent analysis. The primary antibodies used included AMPK (1:800), p-AMPK (1:800), SIRT1 (1:800), PGC-1α (1:800), 4-HNE (1:800; Cat. No. GB150073-100, Servicebio, Wuhan, China), KIM-1 (1:700), NGAL (1:600) and L-FABP (1:600). Except for 4-HNE, all antibodies were the same as those used for Western blotting (see Section 2.9 for catalog numbers and suppliers). Fiji (ImageJ 1.54f) software was used for quantitative analysis of mean optical density of positive staining. All IHC quantification was performed independently by two researchers blinded to the grouping information, with good inter-observer consistency (intraclass correlation coefficient > 0.85). All IHC assays were performed on 3 randomly selected mice per group (*n* = 3 biological replicates).

### 2.11. Statistical Analysis

All data are expressed as mean ± standard deviation (SD), where n represents the number of biological replicates per group. Sample sizes were pre-determined as described in Section 2.1. Statistical analysis was performed using GraphPad Prism 9.0 software (GraphPad Software, San Diego, CA, USA).

Normality of data distribution and homogeneity of variance were verified for all ANOVA models using the Shapiro–Wilk test and Levene test, respectively. Full results of normality and homogeneity tests, together with mean values, SDs and exact P-values for all measured indicators, are provided in Supplementary Tables S1–S4. For overall comparisons across all six experimental groups (CON, HFD, SO, HFD + AE, HFD + API, HFD + AE + API), one-way analysis of variance (one-way ANOVA) followed by Tukey’s post hoc test was applied. If variance was unequal, one-way ANOVA with Welch’s correction, followed by Dunnett’s T3 post hoc test, was used. Prior to the two-way ANOVA, preliminary one-way ANOVA comparisons confirmed that there were no significant differences between the HFD and SO groups for all measured parameters. Therefore, the HFD group was used as the no-apigenin control in the subsequent two-way analysis to avoid over-parameterization.

For the four high-fat-diet subgroups (HFD, HFD + AE, HFD + API, HFD + AE + API), two-way ANOVA was conducted with “apigenin treatment” and “aerobic exercise” as two independent factors to examine the main effect of each intervention and the API × AE interaction effect. Statistical significance was set at *p* < 0.05. A statistically significant interaction effect indicated a non-additive pattern of combined effects, which provides preliminary statistical support for potential synergistic action rather than definitive evidence of biological synergy.

## 3. Results

### 3.1. API Combined with AE Ameliorates Renal Function and Pathological Injury in NAFLD Mice

**Figure 1:**
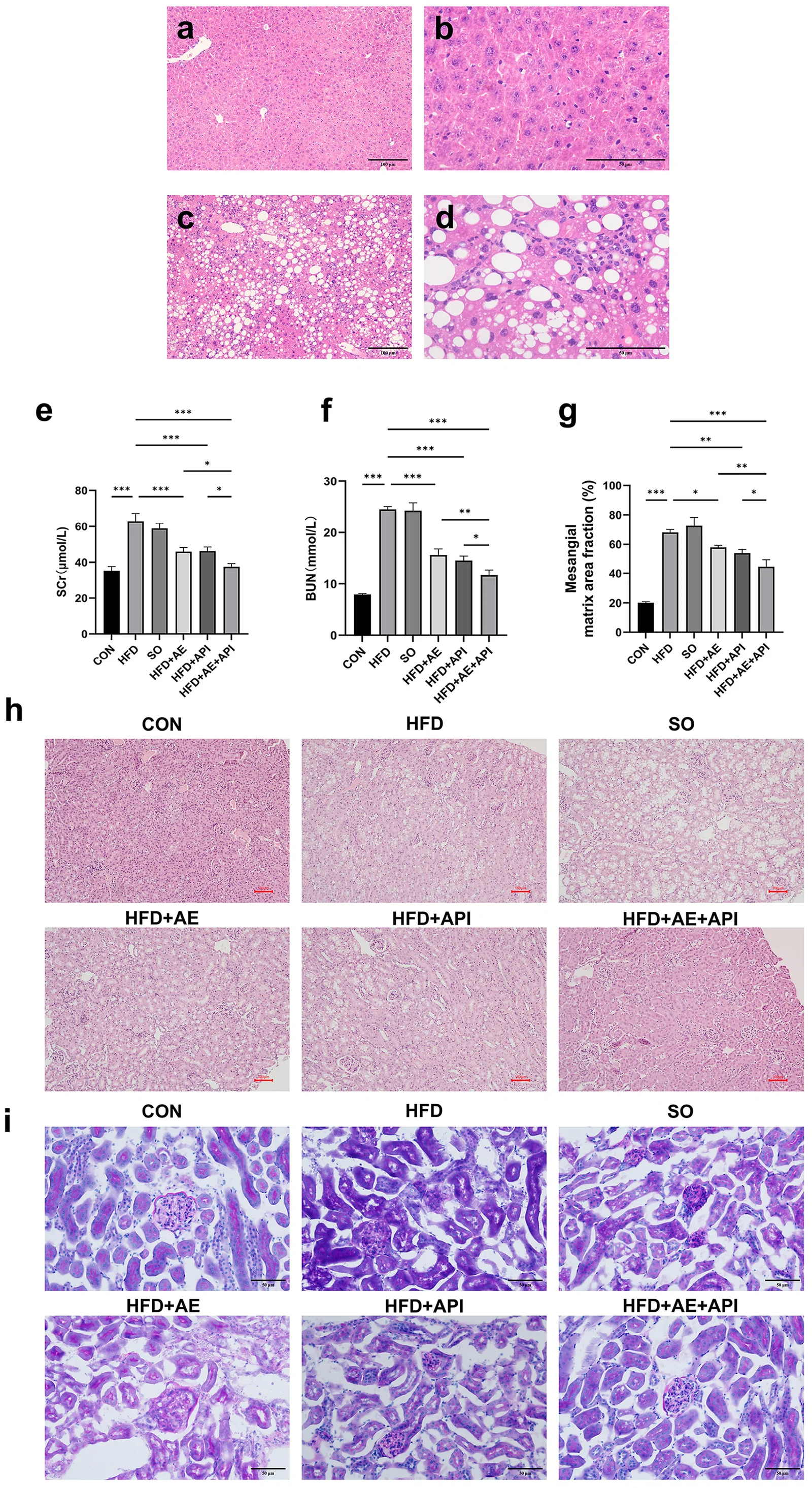
API combined with AE protects against renal dysfunction and histological injury in high-fat-diet-induced NAFLD mice. (**a**–**d**) Representative H&E staining of liver sections from the model validation cohort prior to formal intervention. (**a**,**b**) CON group at 100× and 400× magnification; (**c**,**d**) HFD group at 100× and 400× magnification. HFD induced marked macrovesicular steatosis and hepatocyte ballooning, confirming successful NAFLD modeling. (**e**) Serum creatinine (SCr) levels, (**f**) blood urea nitrogen (BUN) levels, and (**g**) quantitative analysis of mesangial matrix area fraction based on PAS-stained kidney sections across all indicated groups. (**h**) Representative H&E staining and (**i**) PAS staining of kidney sections from the CON, HFD, SO, HFD + AE, HFD + API, and HFD + AE + API groups. Scale bars: 50 μm for panels (**h**,**i**); 100 μm and 400 μm for panels (**a**–**d**) as labeled. Data in panels (**e**–**g**) are presented as mean ± SD. Error bars represent standard deviation (SD). For panels (**e**,**f**) *n* = 5 biologically independent animals per group; for panels (**g**–**i**) *n* = 3 biologically independent animals per group. Statistical analysis: all significance markers shown in panels (**e**–**g**) are derived from one-way ANOVA across all six experimental groups, followed by post hoc multiple comparisons. For panels e and f only, two-way ANOVA was additionally performed on the four HFD-derived subgroups (HFD, HFD + AE, HFD + API, HFD + AE + API) to assess the main effects of API, AE and their interaction; the corresponding F-values and *p*-values are reported in the main text and not marked in the figure. *** *p* < 0.001, ** *p* < 0.01, * *p* < 0.05.

To validate the NAFLD model, liver H&E staining was performed prior to the intervention. HFD feeding for 12 weeks induced marked diffuse macrovesicular steatosis with hepatocyte ballooning and mild lobular inflammation (Figure 1c,d), whereas CON livers preserved normal morphology (Figure 1a,b), confirming successful modeling.

SCr and BUN were significantly elevated in HFD mice compared with the CON group (*p* < 0.001). The SO (1% DMSO vehicle) group did not differ from HFD for any parameter (all *p* > 0.05), excluding solvent interference. Two-way ANOVA revealed significant main effects of both apigenin and aerobic exercise, with significant API × AE interactions for SCr (F(1, 16) = 6.483, *p* = 0.03) and BUN (F(1, 16) = 32.14, *p* < 0.001). Both monotherapies significantly reduced these indicators (*p* < 0.001). Notably, the addition of apigenin to exercise further decreased BUN and SCr beyond the effect of exercise alone (*p* < 0.05; Figure 1e,f).

H&E staining (Figure 1h) revealed that CON kidneys displayed normal architecture with intact glomeruli, well-preserved tubular epithelial cells, and no obvious interstitial abnormalities. In contrast, the HFD group exhibited moderate-to-severe glomerular atrophy with narrowing of the urinary space, widespread tubular epithelial vacuolar degeneration (predominantly proximal tubules), and focal interstitial inflammatory cell infiltrates. These lesions were variably attenuated by monotherapies, whereas the combined intervention resulted in near-complete restoration of normal tubular morphology and markedly reduced interstitial inflammation.

PAS staining (Figure 1i) further substantiated these findings. HFD kidneys showed diffuse glomerular basement membrane thickening, prominent mesangial matrix expansion, extensive tubular brush border loss, and occasional intraluminal proteinaceous casts. Quantitative analysis confirmed that the mesangial matrix area fraction was significantly increased in HFD mice (*p* < 0.001 vs. CON). Both monotherapies significantly reduced this fraction (*p* < 0.05 vs. HFD), and the combination achieved the most pronounced improvement, significantly lower than either monotherapy (*p* < 0.05 for both comparisons; Figure 1g). Thus, the combined API + AE regimen conferred superior structural renoprotection against NAFLD-associated renal injury.

### 3.2. API Combined with AE Regulates Renal Lipid Metabolism Disorder in NAFLD Mice

**Figure 2:**
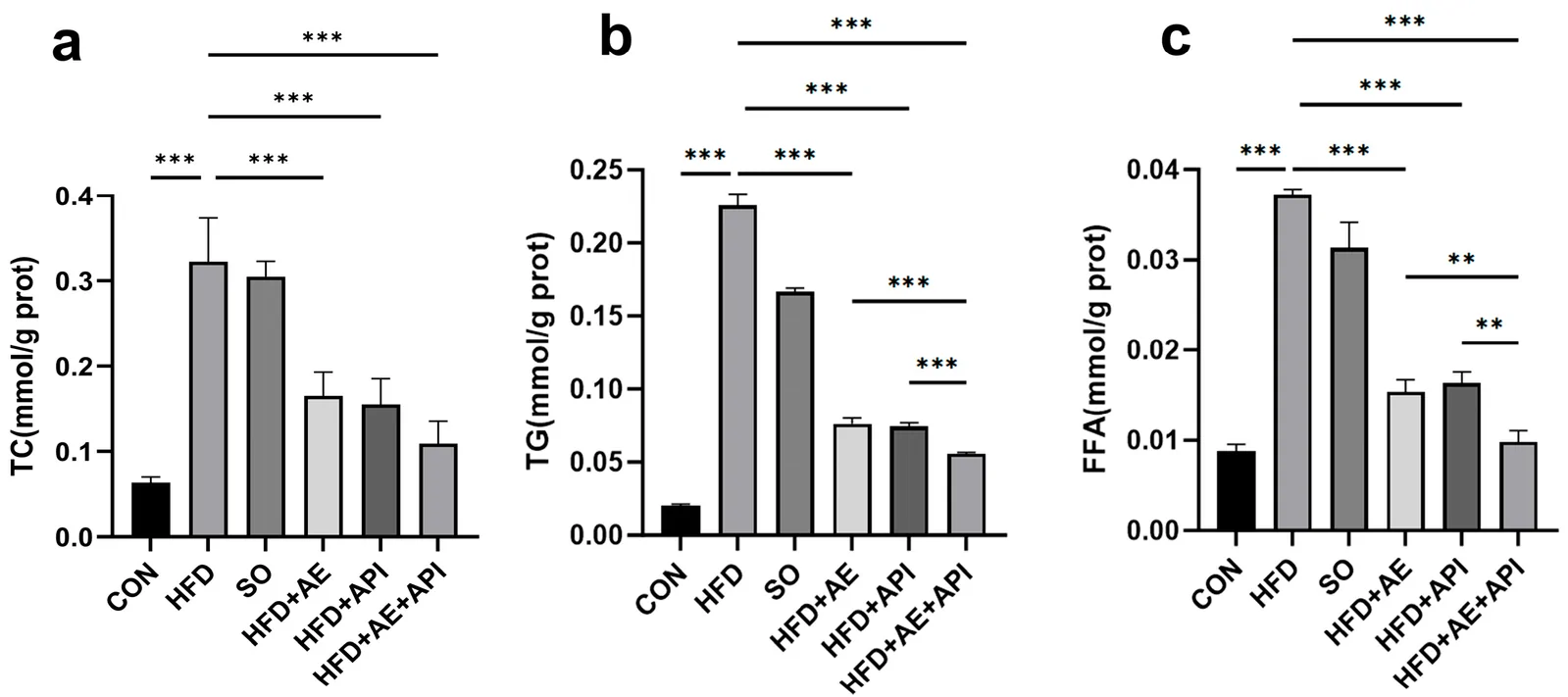
API combined with AE reduces renal lipid accumulation in HFD mice. (**a**) Total cholesterol (TC) content in kidney tissue. (**b**) Triglyceride (TG) content in kidney tissue. (**c**) Free fatty acid (FFA) content in kidney tissue. Data are presented as mean ± SD. Error bars represent standard deviation (SD). *n* = 5 biologically independent animals per group. Statistical analysis: all significance markers shown in the figure are derived from one-way ANOVA across all six experimental groups, followed by post hoc multiple comparisons. Two-way ANOVA was additionally performed on the four HFD-derived subgroups to evaluate the main effects of API, AE and their interaction; the corresponding F-values and *p*-values are reported in the main text and not marked on the figure. *** *p* < 0.001, ** *p* < 0.01.

TC, TG, and FFA contents were significantly increased in HFD mice (all *p* < 0.001 vs. CON). Significant main effects of each intervention and significant API × AE interaction effects were observed for TC (F(1, 16) = 7.539, *p* = 0.03), TG (F(1, 16) = 610.4, *p* < 0.001), and FFA (F(1, 16) = 135.1, *p* < 0.001). All interventions significantly reduced renal lipid contents (*p* < 0.001); the combined intervention produced the largest reduction, which was significantly greater than that of either single intervention (*p* < 0.01; Figure 2). These findings indicate that the combined regimen exerts a superior ameliorative effect on renal ectopic lipid deposition relative to single interventions, and the detected statistical interaction pattern is consistent with potential synergistic action.

### 3.3. API Combined with AE Inhibits Renal Oxidative Stress and Inflammatory Response in NAFLD Mice

**Figure 3:**
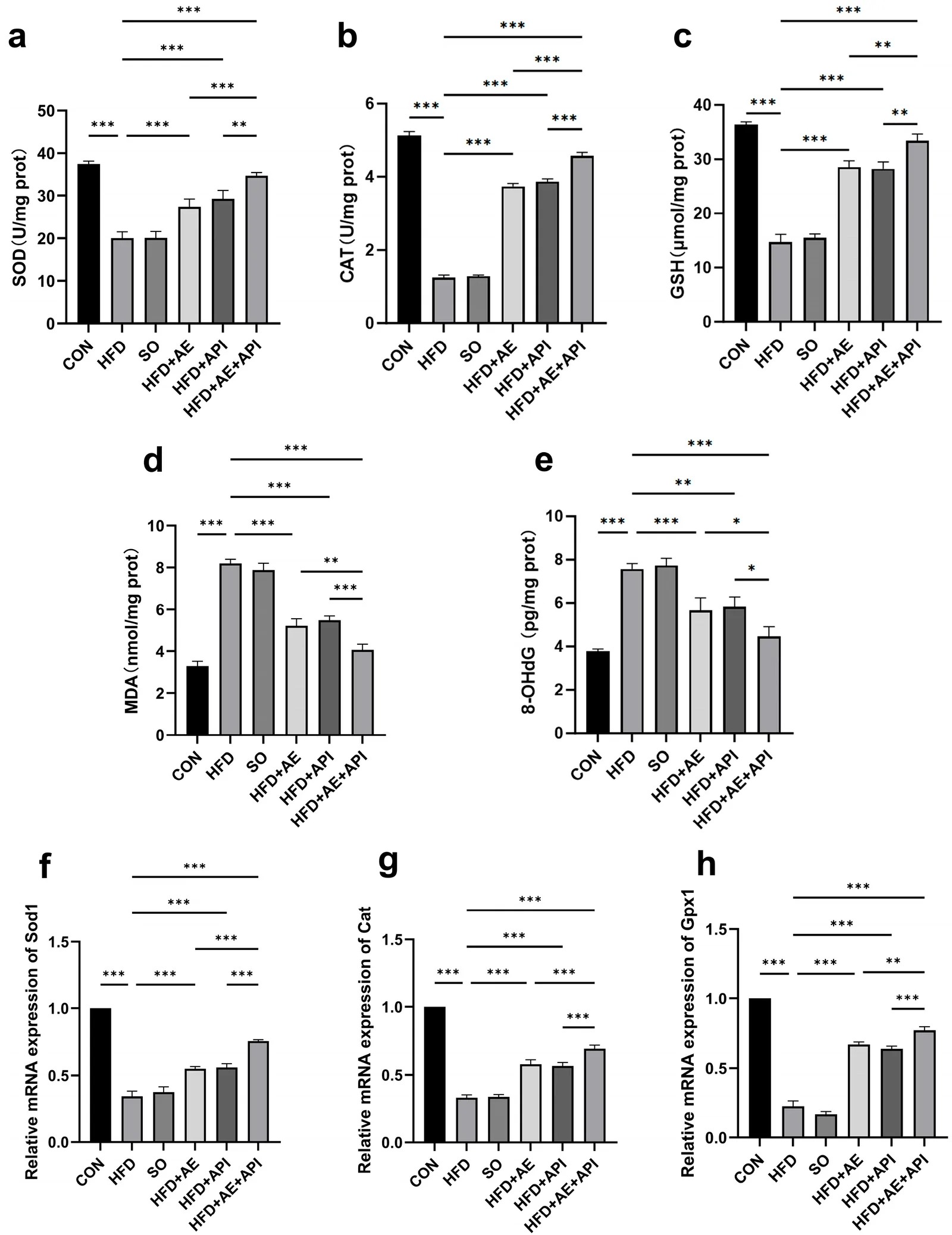
API combined with AE attenuates renal oxidative stress in HFD mice. (**a**) Superoxide dismutase (SOD) activity. (**b**) Catalase (CAT) activity. (**c**) Glutathione (GSH) content. (**d**) Malondialdehyde (MDA) content. (**e**) 8-hydroxy-2′-deoxyguanosine (8-OHdG) content. (**f**) *Sod1* mRNA level. (**g**) *Cat* mRNA level. (**h**) Glutathione peroxidase 1 (*Gpx1*) mRNA level. Data are presented as mean ± SD. Error bars represent standard deviation (SD). For (**a**–**e**), *n* = 5 biologically independent animals per group; for (**f**–**h**), *n* = 3 biologically independent animals per group. Statistical analysis: all significance markers shown in the figure are derived from one-way ANOVA across all six experimental groups, followed by post hoc multiple comparisons. Two-way ANOVA was additionally performed on the four HFD-derived subgroups only for SOD activity (**a**) and MDA content (**d**) to evaluate the main effects of API, AE and their interaction; the corresponding F-values and *p*-values are reported in the main text and not marked on the figure. All remaining endpoints were analyzed by one-way ANOVA only. *** *p* < 0.001, ** *p* < 0.01, * *p* < 0.05.

**Figure 4:**
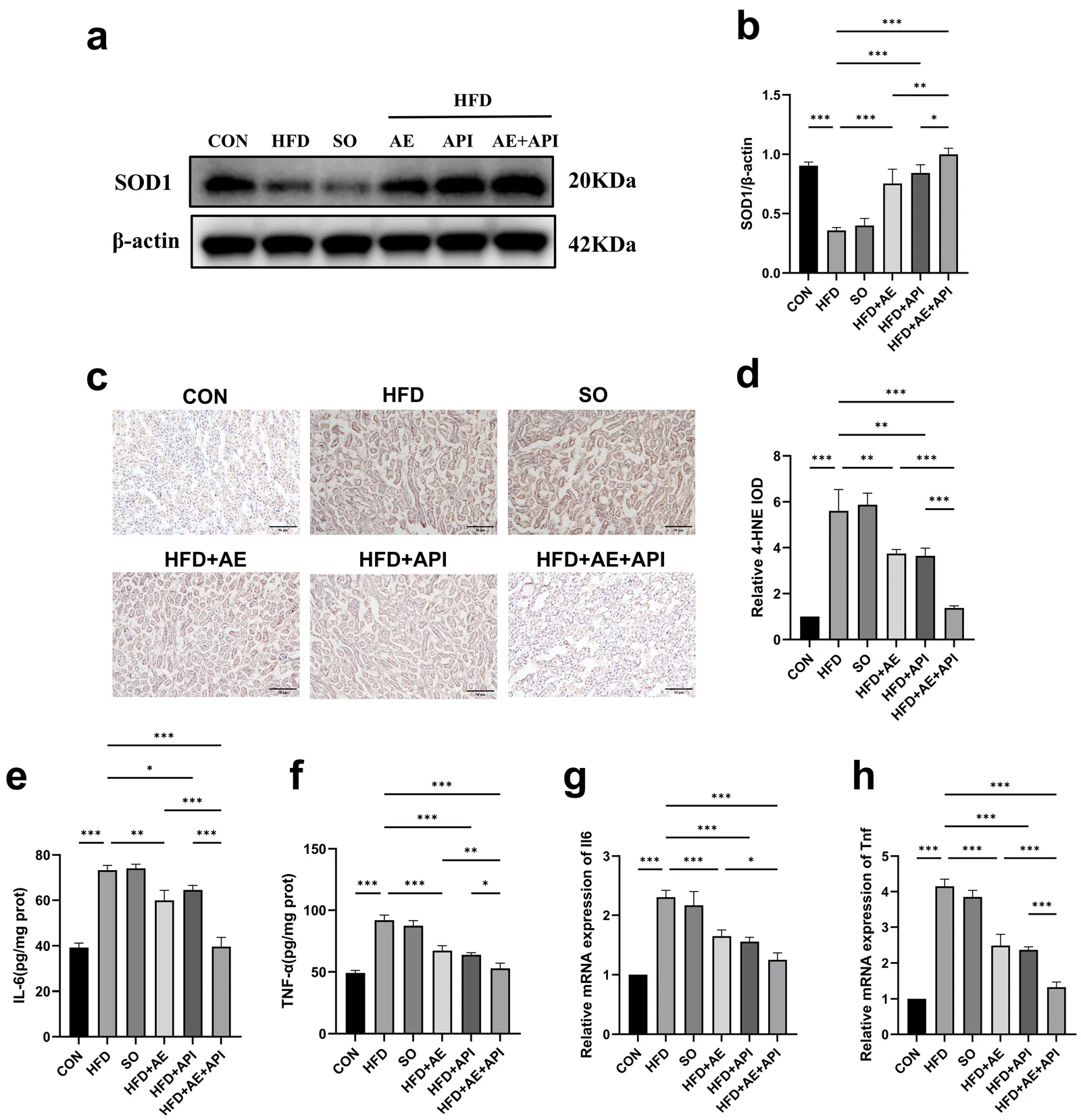
API combined with AE modulates renal antioxidant protein expression, lipid peroxidation, and inflammation in HFD mice. (**a**) Representative Western blot bands of SOD1. (**b**) Quantification of SOD1 protein level normalized to β-actin. (**c**) Representative immunohistochemistry (IHC) staining of 4-hydroxynonenal (4-HNE) in kidney sections. Scale bar, 50 μm. (**d**) Quantification of 4-HNE immunohistochemical staining by relative integrated optical density (IOD). (**e**) Interleukin-6 (IL-6) protein level. (**f**) Tumor necrosis factor-alpha (TNF-α) protein level. (**g**) *Il6* mRNA expression. (**h**) *Tnf* mRNA expression. Data are presented as mean ± SD. Error bars represent standard deviation (SD). For (**e**,**f**), *n* = 5 biologically independent animals per group; for (**a**–**d**,**g**,**h**), *n* = 3 biologically independent animals per group. Statistical analysis: All significance markers shown in the figure are derived from one-way ANOVA across all six experimental groups, followed by post hoc multiple comparisons. Two-way ANOVA was additionally performed on the four HFD-derived subgroups only for IL-6 protein level (**e**) and TNF-α protein level (**f**) to evaluate the main effects of API, AE and their interaction; the corresponding F-values and *p*-values are reported in the main text and not marked in the figure. All remaining endpoints were analyzed by one-way ANOVA only. *** *p* < 0.001, ** *p* < 0.01, * *p* < 0.05. (Original blots are presented in Supplementary Figures S1–S3).

HFD mice exhibited marked oxidative stress, evidenced by significantly decreased SOD and CAT activities and GSH content, alongside elevated MDA and 8-OHdG levels (all *p* < 0.001 vs. CON). Significant API × AE interactions were observed for SOD activity (F(1, 16) = 9.037, *p* = 0.002) and MDA content (F(1, 16) = 26.22, *p* < 0.001). The combination group exerted the most robust antioxidant effect, restoring redox balance toward CON levels (*p* < 0.05 vs. monotherapies; Figure 3a–e). Consistently, mRNA levels of *Sod1*, *Cat*, and *Gpx1*, as well as SOD1 protein expression, were downregulated by HFD (*p* < 0.001) and significantly upregulated by each intervention, with the combination achieving the strongest upregulation (*p* < 0.05; Figure 3f–h, Figure 4a,b). Immunohistochemical staining of 4-HNE confirmed that lipid peroxidation was most profoundly reduced in the combination group (Figure 4c,d).

In parallel, renal inflammatory markers were synchronously elevated. TNF-α and IL-6 protein and mRNA levels were significantly increased in HFD mice (*p* < 0.001 vs. CON). Significant API × AE interactions were detected for both TNF-α (F(1, 16) = 23.17, *p* < 0.001) and IL-6 (F(1, 16) = 9.077, *p* = 0.008). Both interventions significantly reduced these cytokines, and the combination exerted the most prominent anti-inflammatory effect (*p* < 0.05 vs. monotherapies; Figure 4e–h).

### 3.4. API Combined with AE Attenuates Renal Tubular Injury in NAFLD Mice

**Figure 5:**
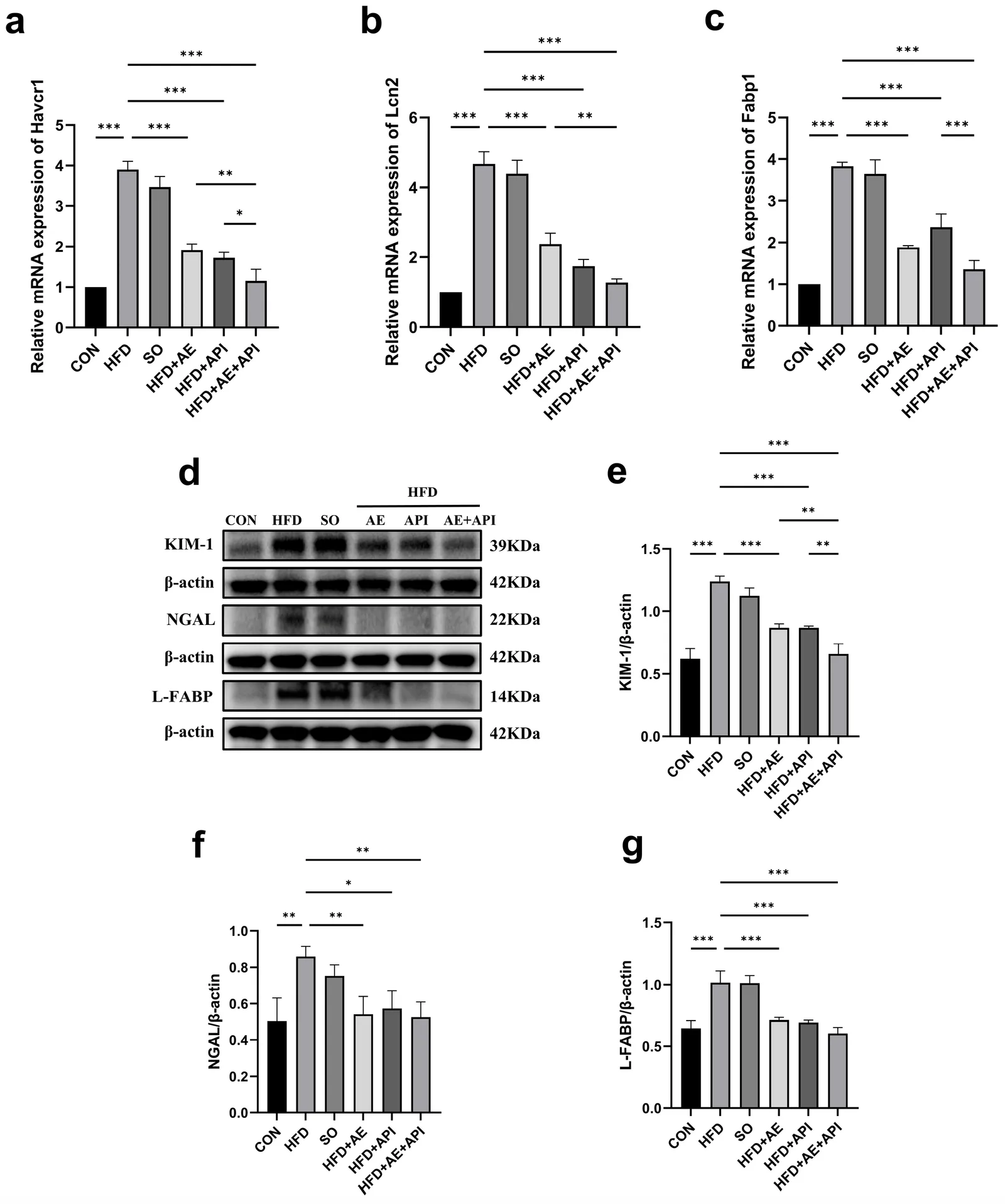
API combined with AE attenuates renal tubular injury markers in HFD mice. (**a**) Hepatitis A virus cellular receptor 1 (*Havcr1*)/kidney injury molecule-1 (KIM-1) mRNA expression. (**b**) Lipocalin2 (*Lcn2*)/neutrophil gelatinase-associated lipocalin (NGAL) mRNA expression. (**c**) Fatty acid binding protein 1 (*Fabp1*)/liver-type fatty acid-binding protein (L-FABP) mRNA expression. (**d**) Representative Western blot images of KIM-1, NGAL, and L-FABP. (**e**) Quantification of KIM-1 protein. (**f**) Quantification of NGAL protein. (**g**) Quantification of L-FABP protein. All protein levels were normalized to β-actin. Data are presented as mean ± SD. Error bars represent standard deviation (SD). *n* = 3 biologically independent animals per group. Statistical analysis: all significance markers shown in the figure are derived from one-way ANOVA across all six experimental groups, followed by post hoc multiple comparisons. Two-way ANOVA was additionally performed on the four HFD-derived subgroups only for the protein levels of KIM-1, NGAL and L-FABP (**e**–**g**) to evaluate the main effects of API, AE and their interaction; the corresponding F-values and *p*-values are reported in the main text and not marked on the figure. The mRNA endpoints (**a**–**c**) were analyzed by one-way ANOVA only. *** *p* < 0.001, ** *p*< 0.01, * *p* < 0.05 (original blots are presented in Supplementary Figures S16–S24).

HFD feeding significantly upregulated the mRNA and protein levels of KIM-1, NGAL, and L-FABP (all *p* < 0.001 vs. CON, Figure 5). At the transcriptional level, *Havcr1* (KIM-1) mRNA was significantly lower in the combination group than in both monotherapy groups (*p* < 0.05). *Lcn2* (NGAL) mRNA in the combination group was lower than in the AE group (*p* < 0.01) but comparable to the API group. *Fabp1* (L-FABP) mRNA reduction was significantly greater in the combination group than in the API group (*p* < 0.001) and similar to the AE group (Figure 5a–c).

**Figure 6:**
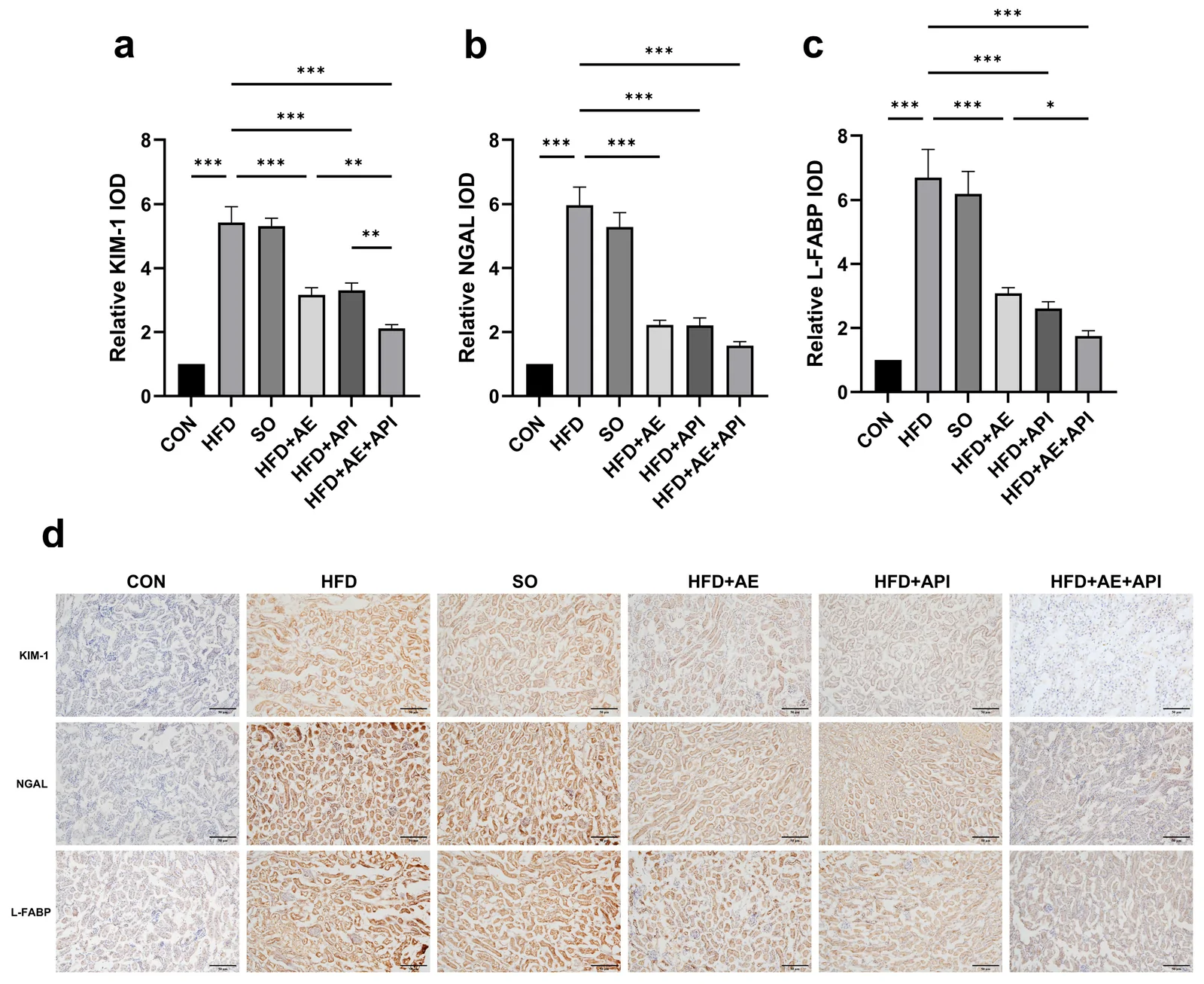
API combined with AE ameliorates renal tubular injury as assessed by immunohistochemistry in HFD mice. (**a**) KIM-1 by relative IOD. (**b**) Quantification of NGAL by relative IOD. (**c**) Quantification of L-FABP by relative IOD. (**d**) Representative IHC staining of KIM-1, NGAL, and L-FABP in kidney sections. Scale bar, 50 μm. Data are presented as mean ± SD. Error bars represent standard deviation (SD). *n* = 3 biologically independent animals per group. Statistical analysis: all significance markers shown in the figure are derived from one-way ANOVA across all six experimental groups, followed by post hoc multiple comparisons. No two-way ANOVA was performed for the endpoints in this figure. *** *p* < 0.001, ** *p*< 0.01, * *p* < 0.05.

At the protein level, two-way ANOVA revealed significant main effects of both interventions and significant API × AE interactions for KIM-1 (F(1, 8) = 8.287, *p* = 0.02), NGAL (F(1, 8) = 7.725, *p* = 0.02), and L-FABP (F(1, 8) = 11.36, *p* < 0.01). KIM-1 protein was significantly lower in the combination group compared with both monotherapies (*p* < 0.001), whereas NGAL and L-FABP exhibited a trend of reduction that failed to attain statistical significance in post hoc analyses (Figure 5d–g). Immunostaining confirmed weak basal expression in CON, strong positive staining in HFD tubular epithelial cells, and markedly reduced intensity in the combination group (Figure 6). (Original blots are presented in Supplementary Figures S4–S12).

### 3.5. API Combined with AE Upregulates Key Molecules of the Renal AMPK/SIRT1/PGC-1α Signaling Pathway

**Figure 7:**
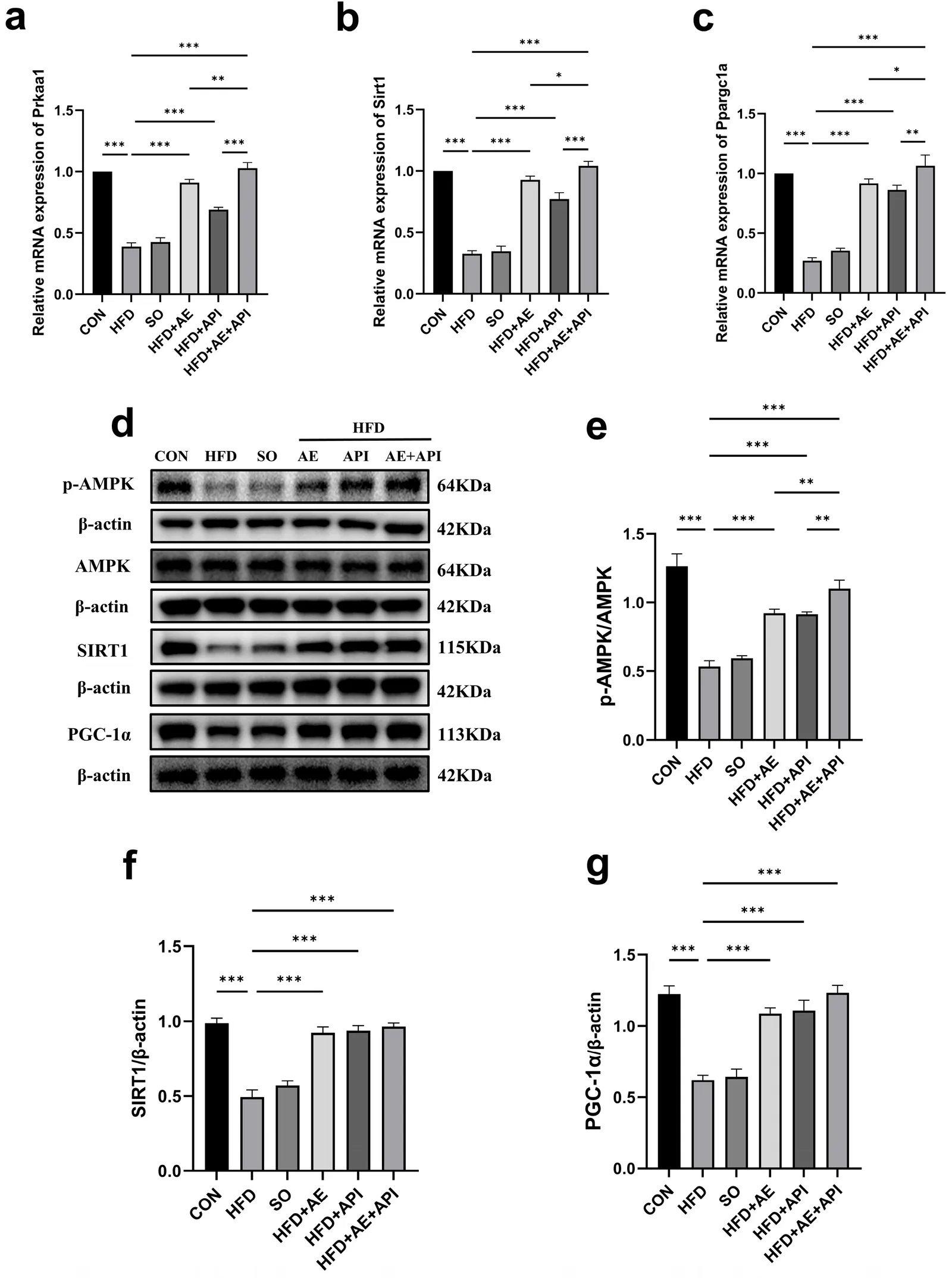
API combined with AE upregulates key molecules of the renal AMPK/SIRT1/PGC-1α signaling pathway at the mRNA and protein levels in HFD mice. (**a**) mRNA expression of protein kinase AMP-activated catalytic subunit alpha 1 (*Prkaa1*; AMP-activated protein kinase, AMPK). (**b**) Sirtuin 1 (*Sirt1)* mRNA expression. (**c**) Peroxisome proliferator-activated receptor gamma coactivator 1-alpha (*Ppargc1a*; PGC-1α) mRNA expression. (**d**) Representative Western blot images of phosphorylated AMPK (p-AMPK), total AMPK, sirtuin 1 (SIRT1), and PGC-1α. (**e**) Quantification of the p-AMPK/AMPK ratio. (**f**) Quantification of SIRT1 protein normalized to β-actin. (**g**) Quantification of PGC-1α protein normalized to β-actin. Data are presented as mean ± SD. Error bars represent standard deviation (SD). *n* = 3 biologically independent animals per group. Statistical analysis: all significance markers shown in the figure are derived from one-way ANOVA across all six experimental groups, followed by post hoc multiple comparisons. Two-way ANOVA was additionally performed on the four HFD-derived subgroups only for the protein endpoints ((**e**–**g**) p-AMPK/AMPK ratio, SIRT1 and PGC-1α protein levels) to evaluate the main effects of API, AE and their interaction; the corresponding F-values and *p*-values are reported in the main text and not marked on the figure. The mRNA endpoints (**a**–**c**) were analyzed by one-way ANOVA only. *** *p* < 0.001, ** *p*< 0.01, * *p* < 0.05. (Original blots are presented in Supplementary Figures S13–S24).

HFD significantly suppressed the renal AMPK/SIRT1/PGC-1α pathway. The *p*-AMPK/total AMPK ratio and SIRT1 and PGC-1α protein levels were significantly decreased compared with CON (all *p* < 0.001), and mRNA levels of *Prkaa1*, *Sirt1*, and *Ppargc1a* were also significantly reduced (all *p* < 0.001). Both interventions upregulated the protein and mRNA expression of key molecules in this signaling cascade, with significant API × AE interactions for p-AMPK/total AMPK (F(1, 8) = 12.43, *p* = 0.008), SIRT1 (F(1, 8) = 89.35, *p* < 0.001), and PGC-1α (F(1, 8) = 38.63, *p* < 0.001). The combination group exhibited the most pronounced upregulation of these pathway molecules (*p* < 0.05 vs. monotherapies; Figure 7).

**Figure 8:**
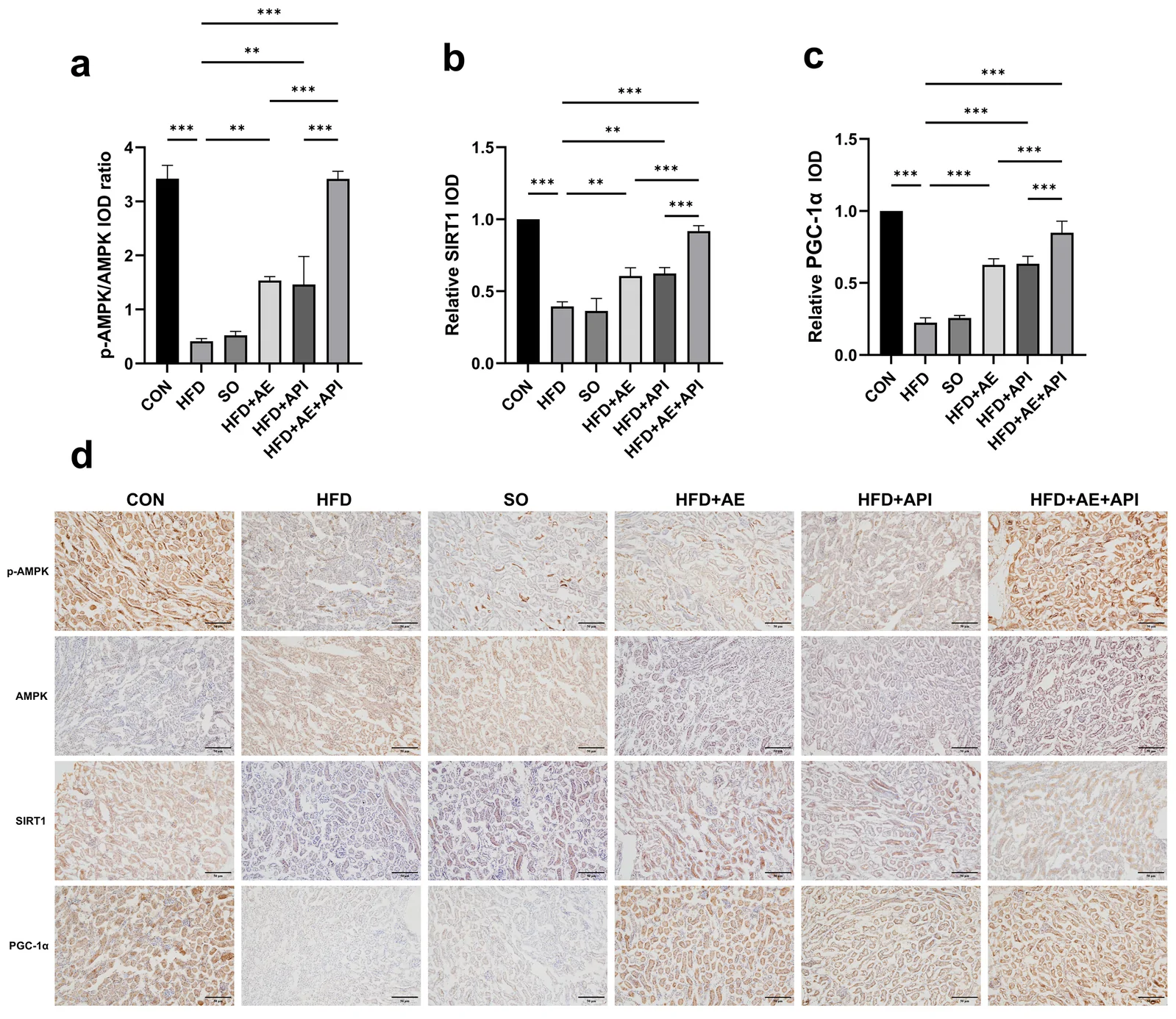
Immunohistochemical validation of the upregulation of key renal AMPK/SIRT1/PGC-1α pathway molecules by API combined with AE in HFD mice. (**a**) Quantification of p-AMPK by relative IOD. (**b**) Quantification of SIRT1 by relative IOD. (**c**) Quantification of PGC-1α by relative IOD. (**d**) Representative IHC staining of AMPK, p-AMPK, SIRT1, and PGC-1α in kidney sections. Scale bar, 50 μm. Data are presented as mean ± SD. Error bars represent standard deviation (SD). *n* = 3 biologically independent animals per group. Statistical analysis: all significance markers shown in the figure are derived from one-way ANOVA across all six experimental groups, followed by post hoc multiple comparisons. No two-way ANOVA was performed for the endpoints in this figure. *** *p* < 0.001, ** *p* < 0.01.

Immunohistochemistry for p-AMPK confirmed these changes, with strongly enhanced positive staining in the combination group (Figure 8). These molecular changes paralleled the improvements in renal lipid metabolism, oxidative stress, inflammation, and tubular injury.

## 4. Discussion

The liver–kidney axis has emerged as a critical pathogenic interface in metabolic diseases, with renal ectopic lipid deposition and subsequent oxidative stress injury constituting the core cascade driving NAFLD-associated chronic kidney disease [32,33]. While apigenin and aerobic exercise each have documented renoprotective properties in separate disease models, their combined efficacy and underlying mechanisms in NAFLD-associated renal injury remain poorly characterized. Using a high-fat-diet-induced NAFLD mouse model, the present study demonstrates that combined apigenin and aerobic exercise intervention exerts an augmented protective effect against renal dysfunction and tubular injury, which is accompanied by improved renal lipid metabolism, suppressed oxidative stress and inflammation, and upregulated phosphorylation and protein expression of key molecules in the AMPK/SIRT1/PGC-1α signaling pathway. These findings provide further evidence of a significant statistical interaction between the dietary flavonoid and aerobic exercise in attenuating NAFLD-associated renal injury, an effect pattern consistent with potential synergistic protection.

To investigate the upstream mechanisms underlying this enhanced renoprotective effect, we first examined renal lipid deposition, the established initiating event in NAFLD-related renal injury. Dysregulated lipid metabolism and ectopic renal lipid deposition are the initiating event of NAFLD-related renal injury, preceding overt renal dysfunction [34,35]. The present study confirmed that sustained high-fat feeding induced overt renal lipid deposition and functional decline, replicating the established NAFLD-associated renal injury phenotype [36]. At the intervention level, monotherapy with either apigenin or aerobic exercise partially ameliorated renal lipid deposition and improved renal function, consistent with findings from prior studies in acute kidney injury and obese mouse models [37]. This lipid-modulating property of apigenin, combined with its natural abundance and favorable safety profile, makes apigenin a particularly attractive candidate for chronic intervention in metabolic renal diseases. The combined intervention yielded significantly greater improvements than either single intervention, and two-way ANOVA confirmed significant statistical interactions for all lipid parameters and renal function indicators, an effect pattern consistent with potential synergistic action. This superior lipid-lowering effect may be attributed to complementary modes of action at the systemic and tissue levels: aerobic exercise primarily reduces circulating lipid flux through increased whole-body energy expenditure and fatty acid oxidation [38], whereas apigenin directly modulates intracellular lipid synthesis and oxidation pathways in renal tubular epithelial cells [3]. Together, these dual actions exert a more potent blockade of upstream lipotoxicity than either intervention alone, laying a pathological foundation for the alleviation of downstream renal injury.

Ectopic lipid accumulation triggers a downstream pathogenic cascade centered on oxidative stress and inflammation, which ultimately converges on renal tubular epithelial cell injury [4,39]. Excess free fatty acids promote excessive ROS production and lipid peroxidation, generating cytotoxic end products such as MDA and 4-HNE that directly damage renal parenchymal cells and activate pro-inflammatory signaling, forming a vicious cycle that drives progressive tubular injury [40,41]. The present study recapitulated this entire pathogenic cascade in NAFLD mice. Specifically, we observed a concurrent suppression of endogenous antioxidant capacity, elevation of lipid peroxidation products and pro-inflammatory cytokines, and upregulation of tubular injury markers. The combined intervention exhibited markedly enhanced inhibition across this entire pathogenic cascade, outperforming both monotherapies across antioxidant and anti-inflammatory endpoints. Mechanistically, this enhanced protective effect, consistent with a pattern of potential synergy, likely arises from multi-level targeting of the cascade: apigenin, as a flavonoid with direct free radical scavenging activity, acts primarily at the molecular level to neutralize excess ROSs and suppress pro-inflammatory cytokine transcription [42]. Additionally, apigenin has been identified as an aryl hydrocarbon receptor (AhR) antagonist, which may contribute to its anti-inflammatory effects in renal injury by inhibiting AhR-STAT3 signaling [43]. In contrast, aerobic exercise acts upstream of the cascade by improving systemic lipid metabolism and reducing the initial trigger of lipotoxicity, while also upregulating endogenous antioxidant enzyme expression through long-term metabolic adaptation [44]. By simultaneously targeting the upstream metabolic trigger and downstream molecular damage, the combined regimen may interrupt the amplification cycle between oxidative stress and inflammation more effectively than single interventions, which in turn may translate into greater protection against renal tubular injury.

Notably, the parallel changes in tubular injury markers with lipid and oxidative stress parameters further support that the protective effect of the combined intervention may be attributable, at least in part, to the correction of upstream metabolic and redox disorders rather than direct action on tubular cells. KIM-1, NGAL and L-FABP are sensitive indicators of early tubular epithelial injury, and their expression levels correlate with the severity of renal damage [45,46]. Interestingly, while the combined intervention produced a consistent supra-additive effect on KIM-1, the reduction in NGAL and L-FABP proteins did not reach statistical significance compared with apigenin monotherapy. This may reflect the differential sensitivity of these markers to exercise-induced hemodynamic changes or differences in their tubular expression kinetics. Notably, this disparity indirectly supports the notion that the combined intervention alleviates tubular injury primarily by correcting upstream metabolic and redox disturbances, rather than through a non-specific pan-suppression of all injury markers. Consequently, KIM-1 might serve as a more reliable indicator of the additive renoprotective benefit conferred by aerobic exercise in this model.

To explore the molecular basis underlying the observed renoprotective effects, we focused on the AMPK/SIRT1/PGC-1α signaling axis, a central regulatory network that couples cellular energy status to lipid metabolism, mitochondrial function and antioxidant defense [27]. In line with previous findings in diabetic nephropathy and obesity-related renal injury [47], our results showed that renal AMPK phosphorylation and downstream SIRT1 and PGC-1α expression were significantly downregulated in NAFLD mice, indicating that suppression of this pathway is a characteristic molecular feature of NAFLD-associated renal damage. Mechanistically, the upregulation of key molecules in this pathway likely arises from converging yet independent upstream inputs: aerobic exercise elevates the cellular AMP/ATP ratio and activates liver kinase B1 (LKB1) in response to increased energy consumption, while apigenin directly sensitizes the AMPK γ subunit and inhibits protein phosphatases responsible for AMPK dephosphorylation [48]. These dual, non-redundant triggers would drive upregulation of the AMPK/SIRT1/PGC-1α axis at the phosphorylation and protein expression levels. This dual regulatory mode could suppress the lipotoxic cascade to an extent that aligns with the observed effect size, significantly exceeding the expected additive effect of either single intervention, an effect pattern consistent with potential synergistic action.

Upregulation of AMPK/SIRT1/PGC-1α pathway molecules offers a plausible unifying molecular hypothesis for the multi-level phenotypic improvements observed in this study. PGC-1α, as the terminal effector of the cascade, is known to coordinately promote fatty acid oxidation, mitochondrial biogenesis and endogenous antioxidant enzyme expression. Thus, upregulated expression of key pathway molecules could simultaneously reduce renal ectopic lipid accumulation, strengthen cellular antioxidant defense, and suppress inflammatory responses, thereby potentially attenuating the entire lipotoxic injury cascade. However, it should be emphasized that the present study only demonstrates a strong correlation between upregulation of these pathway molecules and renal protection at the whole-animal level. In the absence of pathway blockade experiments using specific inhibitors or genetic knockout models, a strict causal relationship cannot be established, and the involvement of alternative signaling pathways cannot be excluded.

Several limitations of this study should be acknowledged. First, the study was conducted exclusively in male C57BL/6 mice. Given that estrogen has been reported to modulate AMPK activity and influence the progression of NAFLD and related renal complications, future studies including female animals are necessary to evaluate potential sex differences in the efficacy of the combined intervention. Second, only conventional serum biomarkers (SCr and BUN) were used to assess renal function, and more sensitive and specific indicators such as urinary albumin-to-creatinine ratio, cystatin C, and estimated glomerular filtration rate were not evaluated. SCr levels can be influenced by muscle mass and hydration status, which may be modulated by aerobic exercise intervention, introducing potential confounding in the interpretation of renal function outcomes. While SCr and BUN remain the most widely used classical indicators for renal function assessment in preclinical studies, incorporation of additional sensitive renal biomarkers will be important in future studies to more accurately characterize the extent of renal injury. Third, the sample size for mechanistic endpoints (including Western blotting, immunohistochemistry, and quantitative real-time PCR) was relatively small (*n* = 3 per group), resulting in limited statistical power for detecting interaction effects in the two-way ANOVA (df = 8 for the interaction term). Although several interaction terms reached very low *p*-values, conclusions regarding the interaction effects should be interpreted with caution. Future studies with larger sample sizes are warranted to verify the robustness of these findings. Fourth, this study was exploratory in nature and encompassed a broad panel of biological endpoints covering renal function, renal lipid accumulation, oxidative stress, inflammatory response, tubular injury, and signaling pathway molecules. While post hoc multiple comparison corrections were applied within each individual family of endpoints, no adjustment for the family-wise error rate was performed across the full set of all tested endpoints. As a result, there is a potential risk of type I error inflation across the entire endpoint panel. Accordingly, the overall findings of this study should be regarded as exploratory results that require further validation in dedicated confirmatory studies. Fifth, the causal role of the AMPK/SIRT1/PGC-1α pathway has not been functionally verified. Subsequent studies using AMPK-specific inhibitors (e.g., compound C) or proximal tubule-specific AMPK knockout models are required to confirm whether this pathway is necessary for the observed renoprotective effects. Sixth, apigenin was administered via intraperitoneal injection in this study, which bypasses first-pass metabolism and achieves higher systemic bioavailability than oral administration. While this route is appropriate for proof-of-concept mechanistic studies, translational application would require formulation strategies (e.g., nanoparticles or co-administration with absorption enhancers) to improve oral bioavailability in clinical settings. Seventh, only a single dose of apigenin and a single exercise intensity were employed. Dose–response studies with gradient apigenin concentrations and exercise intensities are needed to define the optimal combination regimen and clarify the dose–effect relationship. Eighth, direct assessments of mitochondrial function (e.g., mitochondrial ROS production, ATP content, mtDNA copy number) were not performed, so the impact of PGC-1α upregulation on mitochondrial quality and function remains to be confirmed. Finally, this study focused on renal local pathology and did not systematically evaluate hepatic injury or liver–kidney axis crosstalk; further investigation of hepatic–renal metabolic communication would strengthen the translational relevance of the findings. In particular, future studies incorporating systematic hepatic pathology assessments and key mediators of liver–kidney crosstalk, such as FGF21 and specific lipid species, will be essential to fully delineate the in vivo network through which this combined pharmacological and exercise strategy exerts its systemic benefits.

## 5. Conclusions

Collectively, this study provides convergent evidence that combined apigenin and aerobic exercise intervention ameliorates high-fat-diet-induced renal injury in NAFLD mice, associated with an improvement in lipid metabolism, the suppression of oxidative stress and inflammation, and the upregulation of key molecules of the AMPK/SIRT1/PGC-1α signaling pathway. The significant interaction effect between the two interventions across multiple pathological endpoints suggests that combined pharmacological and non-pharmacological strategies may offer superior efficacy over monotherapy for NAFLD-associated renal complications. These findings provide proof-of-concept preclinical evidence supporting the potential of combined lifestyle and nutritional intervention strategies, and lay a foundation for further investigation of the underlying molecular mechanisms.

## Figures and Tables

**Table 1:** Primer sequences used for quantitative real-time PCR.

Gene	Sequence	Primer Size	Product Size
*Prkaa1* (AMPKα1)_F	CTCTTTCCTGGACGACCACC	20	140
*Prkaa1* (AMPKα1)_R	TTGCCTTCCGTACACCTTGG	20	
*Sirt1*_F	TCGGCTACCGAGGTCCATA	19	136
*Sirt1*_R	ACAATCTGCCACAGCGTCAT	20	
*Ppargc1a* (PGC-1α)_F	GTCCTTCCTCCATGCCTGAC	20	103
*Ppargc1a* (PGC-1α)_R	TAGCTGAGCTGAGTGTTGGC	20	
*Sod1*_F	TTGTGGTGTCAGGACAGATTAC	22	92
*Sod1*_R	AGTGGTACAGCCTTGTGTATTG	22	
*Cat*_F	CGGCACATGAATGGCTATGGATC	23	152
*Cat*_R	AAGCCTTCCTGCCTCTCCAACA	22	
*Gpx1*_F	CGCTCTTTACCTTCCTGCGGAA	22	146
*Gpx1*_R	AGTTCCAGGCAATGTCGTTGCG	22	
*Tnf* (TNF-α)_F	ATCGGTCCCAACAAGGAGGA	20	138
*Tnf* (TNF-α)_R	CGCTTGGTGGTTTGCTACG	19	
*Il6* (IL-6)_F	CCAGTTGCCTTCTTGGGACT	20	103
*Il6* (IL-6)_R	CTGGTCTGTTGTGGGTGGTA	20	
*Havcr1* (KIM-1)_F	AGGAAGTCAGCATCTCTAAGCG	22	97
*Havcr1* (KIM-1)_R	TGTTGTCTTCAGCTCGGGAA	20	
*Lcn2* (NGAL)_F	GACTTCCGGAGCGATCAGTT	20	188
*Lcn2* (NGAL)_R	CTGATCCAGTAGCGACAGCC	20	
*Fabp1* (L-FABP)_F	AGAGCCAGGAGAACTTTGAGC	21	157
*Fabp1* (L-FABP)_R	TCATTGCGGGACCACTTTGGG	20	
*Gapdh*_F	CATCACTGCCACCCAGAAGACTG	23	153
*Gapdh*_R	ATGCCAGTGAGCTTCCCGTTCAG	23	

## Data Availability

The data presented in this study are available on request from the corresponding author due to restrictions associated with ongoing related research projects.

## Supplementary Materials

The following supporting information can be downloaded at https://www.mdpi.com/article/10.3390/cimb48090942/s1.

## Abbreviations

The following abbreviations are used in this manuscript:

8-OHdG	8-hydroxy-2′-deoxyguanosine
4-HNE	4-hydroxynonenal
AE	aerobic exercise
AMPK	AMP-activated protein kinase
API	Apigenin
CAT	catalase
CKD	chronic kidney disease
ELISA	enzyme-linked immunosorbent assay
FFA	free fatty acid
GPx	glutathione peroxidase
H&E	hematoxylin and eosin
HFD	high-fat diet
IHC	immunohistochemical
IL-6	interleukin-6
KIM-1	kidney injury molecule-1
L-FABP	liver-type fatty acid-binding protein
NAFLD	non-alcoholic fatty liver disease
MDA	malondialdehyde
NGAL	neutrophil gelatinase-associated lipocalin
p-AMPK	phosphorylated AMP-activated protein kinase
PGC-1α	peroxisome proliferator-activated receptor gamma coactivator 1-alpha
qRT-PCR	quantitative real-time PCR
ROS	reactive oxygen species
SOD	superoxide dismutase
TC	total cholesterol
TG	triglyceride
TNF-α	tumor necrosis factor-α
