# Supplementary material for: Apigenin Combined with Aerobic Exercise Is Associated with Attenuated Renal Oxidative Stress and AMPK/SIRT1/PGC-1α Pathway Activation in NAFLD Mice
Source: Curr Issues Mol Biol. 2026 Sep 15;48(9):942. doi: 10.3390/cimb48090942 (PMC13605681; doi:10.3390/cimb48090942)
Supplement: Supplementary file 1 [file cimb-48-00942-s001.zip › Supplementary Files/Supplementary Figures.pdf]

SOD1 (1)

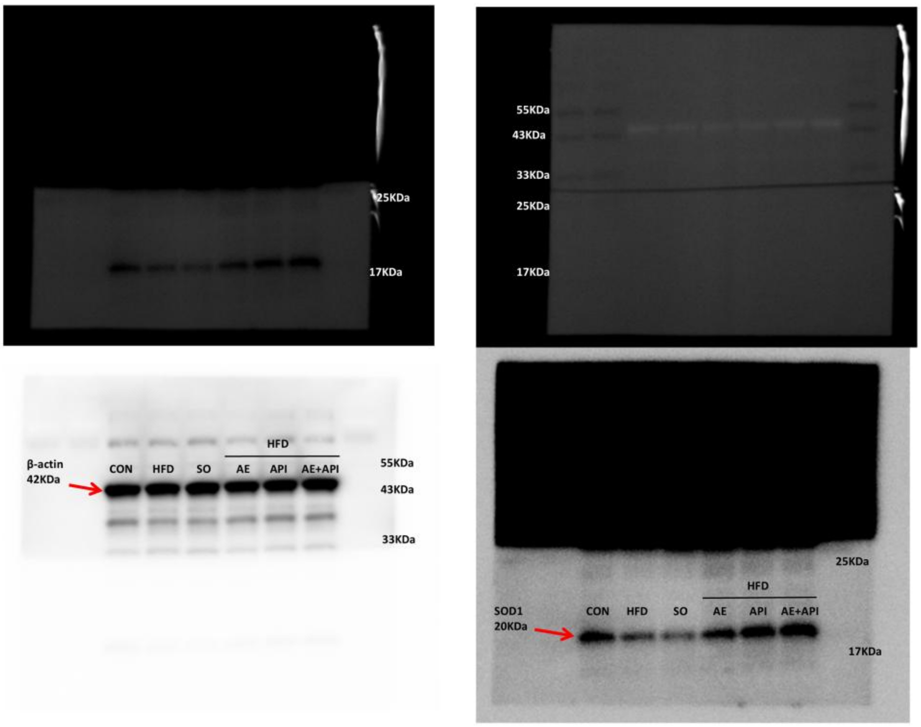

Supplementary Figure S1： Full-length blot image of SOD1 (Replicate 1). Red arrows indicate the target protein and GAPDH bands. Molecular weight markers are indicated in the image.

SOD1(2)

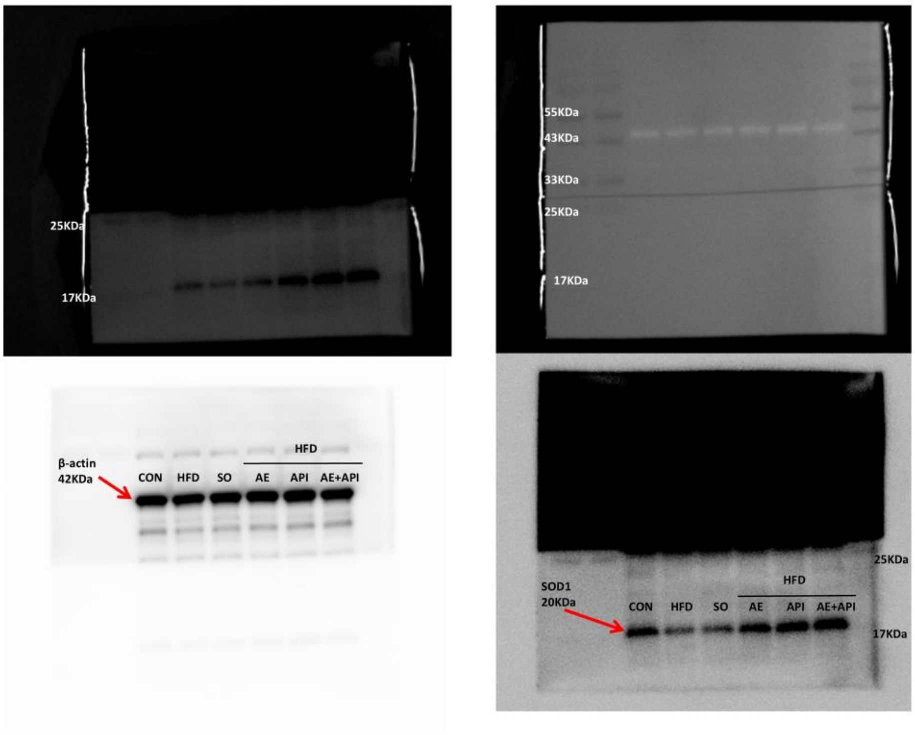

Supplementary Figure S2： Full-length blot image of SOD1 (Replicate 2). Red arrows indicate the target protein and GAPDH bands. Molecular weight markers are indicated in the image.

SOD1 (3)

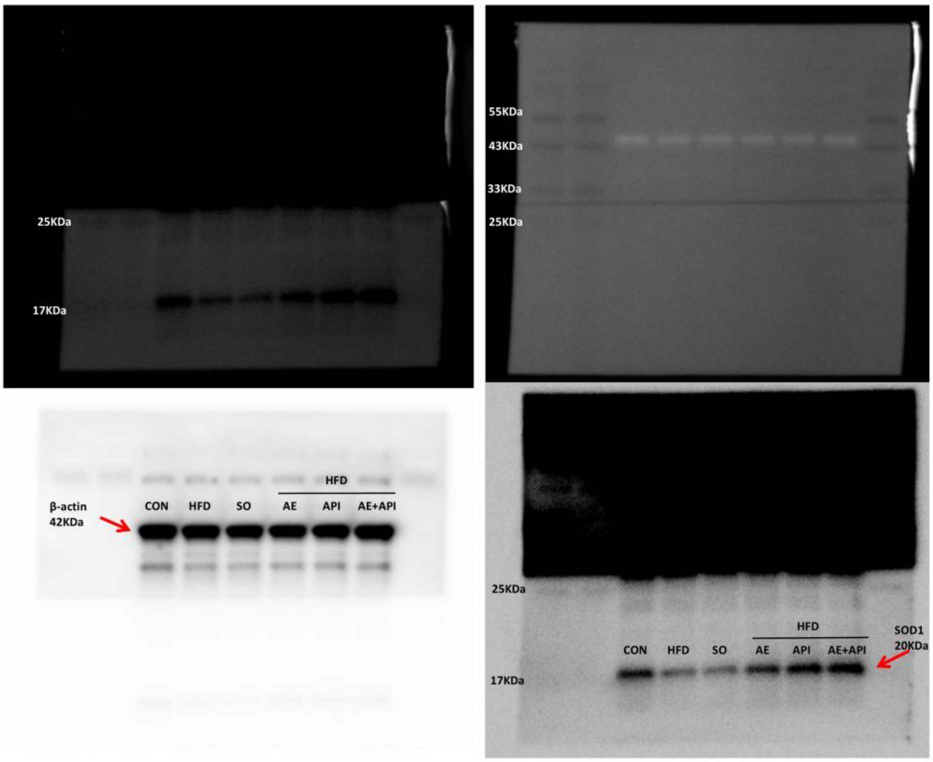

Supplementary Figure S3： Full-length blot image of SOD1 (Replicate 3). Red arrows indicate the target protein and GAPDH bands. Molecular weight markers are indicated in the image.

KIM-1(1)

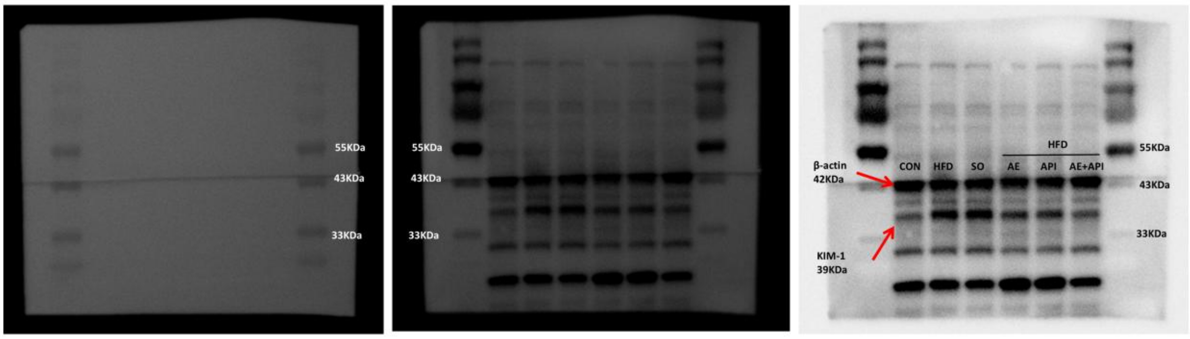

Supplementary Figure S4： Full-length blot image of KIM-1 (Replicate 1). Red arrows indicate the target protein and GAPDH bands. Molecular weight markers are indicated in the image.

KIM-1(2)

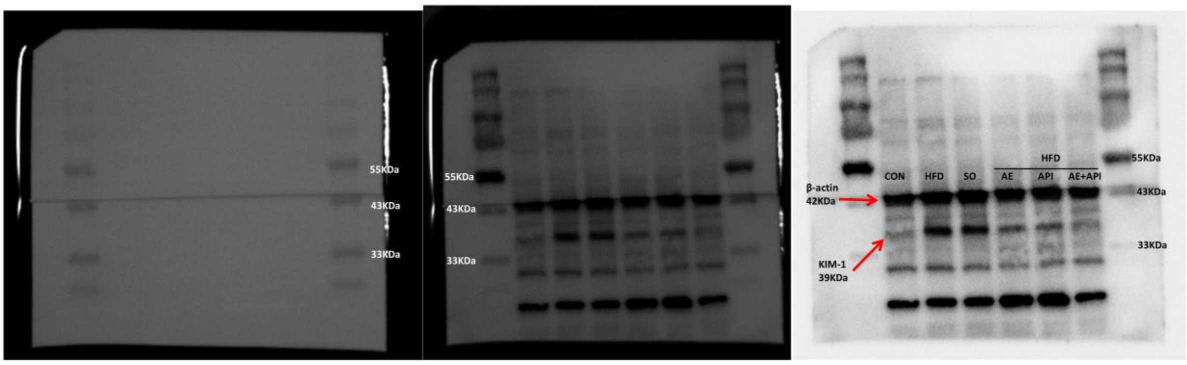

Supplementary Figure S5： Full-length blot image of KIM-1 (Replicate 2). Red arrows indicate the target protein and GAPDH bands. Molecular weight markers are indicated in the image.

KIM-1(3)

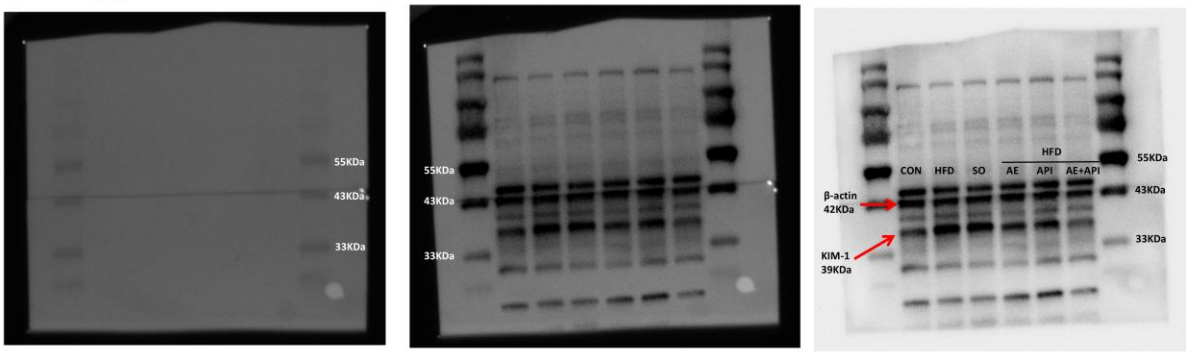

Supplementary Figure S6： Full-length blot image of KIM-1 (Replicate 3). Red arrows indicate the target protein and GAPDH bands. Molecular weight markers are indicated in the image.

NGAL(1)

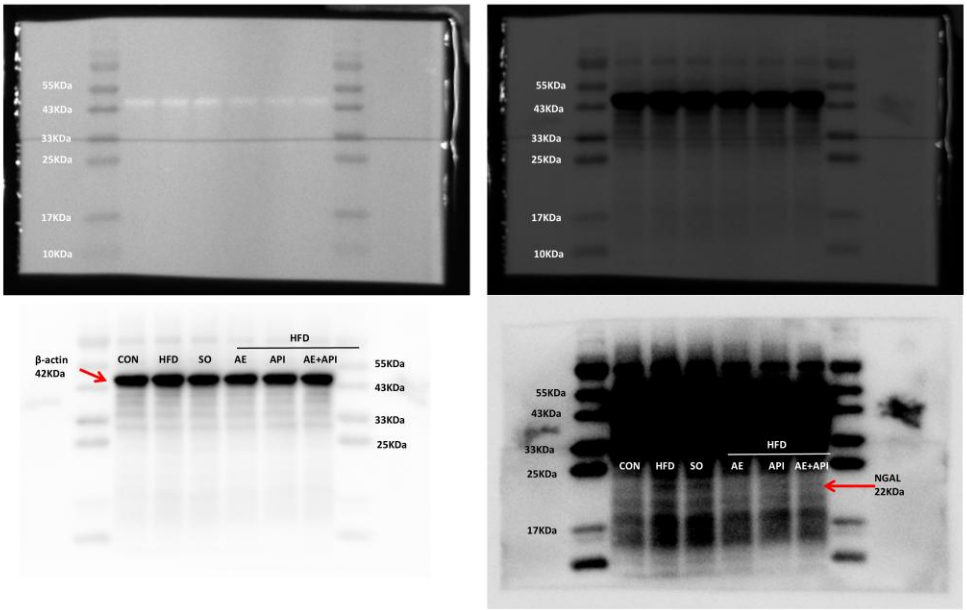

Supplementary Figure S7： Full-length blot image of NGAL (Replicate 1). Red arrows indicate the target protein and GAPDH bands. Molecular weight markers are indicated in the image.

NGAL(2)

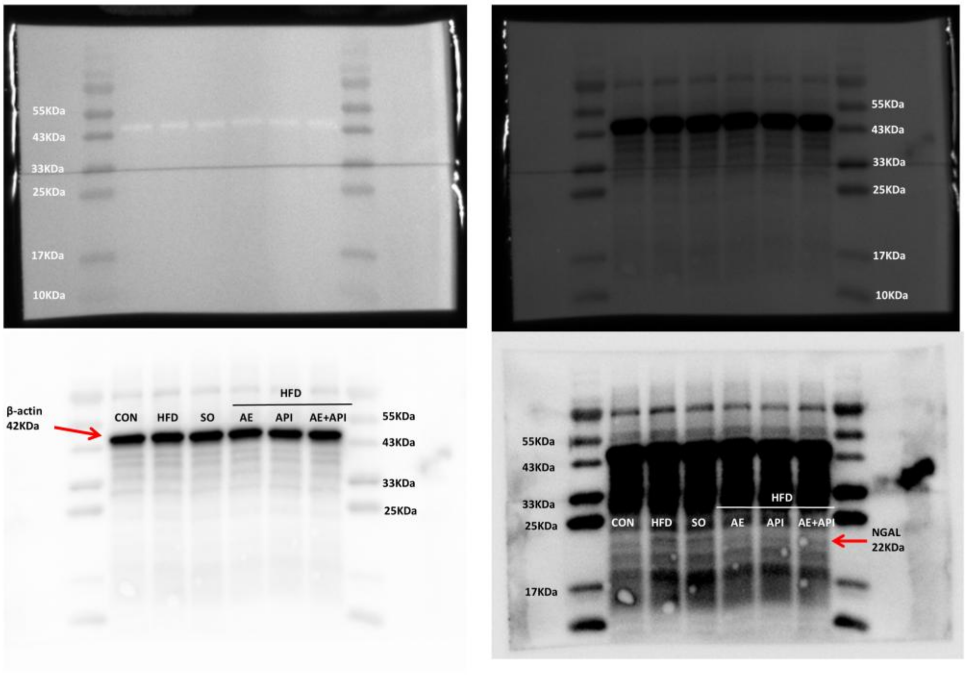

Supplementary Figure S8： Full-length blot image of NGAL (Replicate 2). Red arrows indicate the target protein and GAPDH bands. Molecular weight markers are indicated in the image.

NGAL(3)

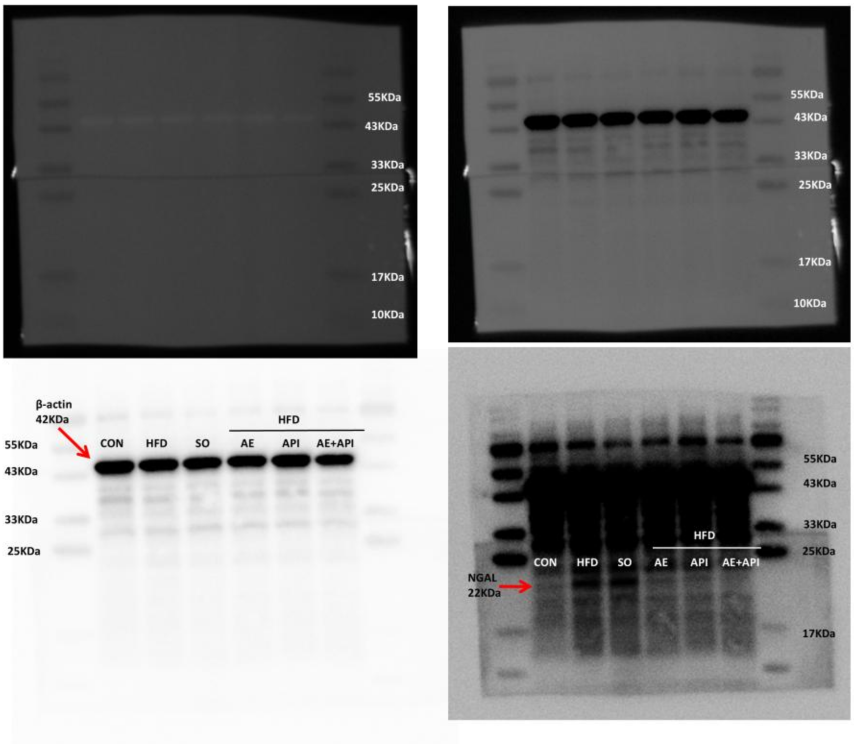

Supplementary Figure S9： Full-length blot image of NGAL (Replicate 3). Red arrows indicate the target protein and GAPDH bands. Molecular weight markers are indicated in the image.

L-FABP(1)

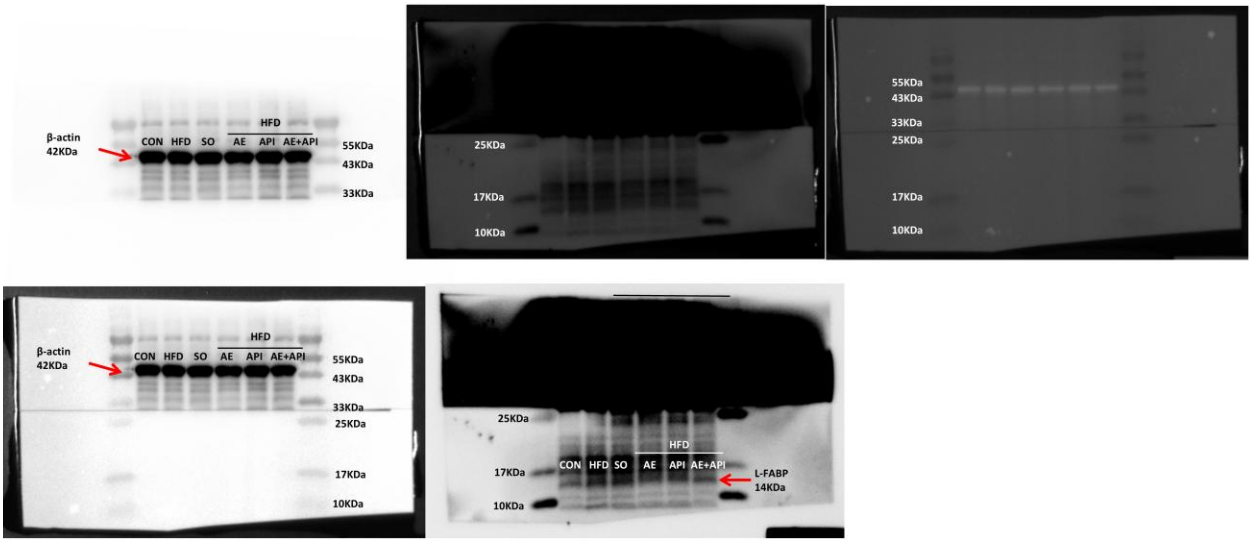

Supplementary Figure S10： Full-length blot image of L-FABP (Replicate 1). Red arrows indicate the target protein and GAPDH bands. Molecular weight markers are indicated in the image.

L-FABP(2)

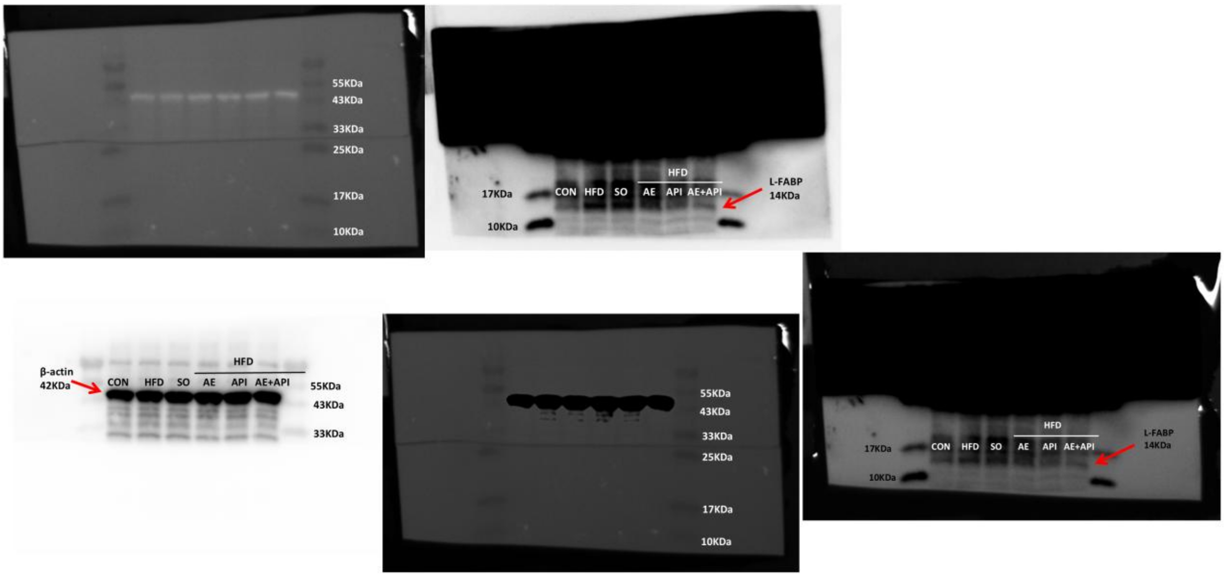

Supplementary Figure S11：　Full-length blot image of L-FABP (Replicate 2). Red arrows indicate the target protein and GAPDH bands. Molecular weight markers are indicated in the image.

L-FABP(3)

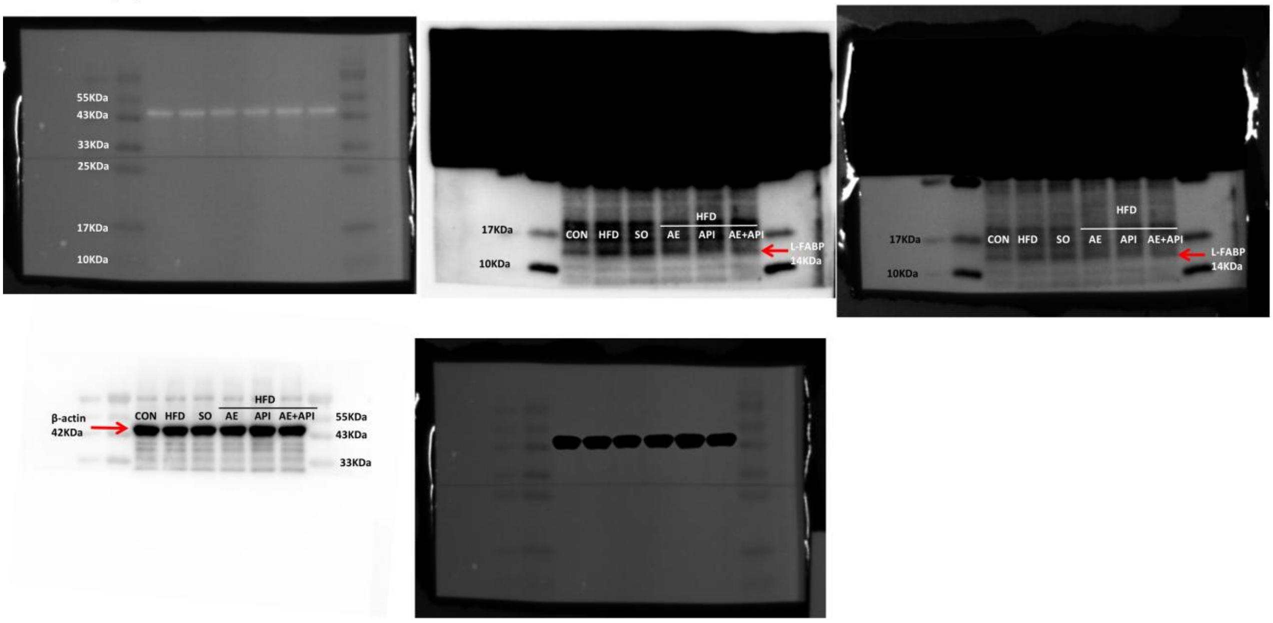

Supplementary Figure S12：　Full-length blot image of L-FABP (Replicate 3). Red arrows indicate the target protein and GAPDH bands. Molecular weight markers are indicated in the image.

AMPK (1)

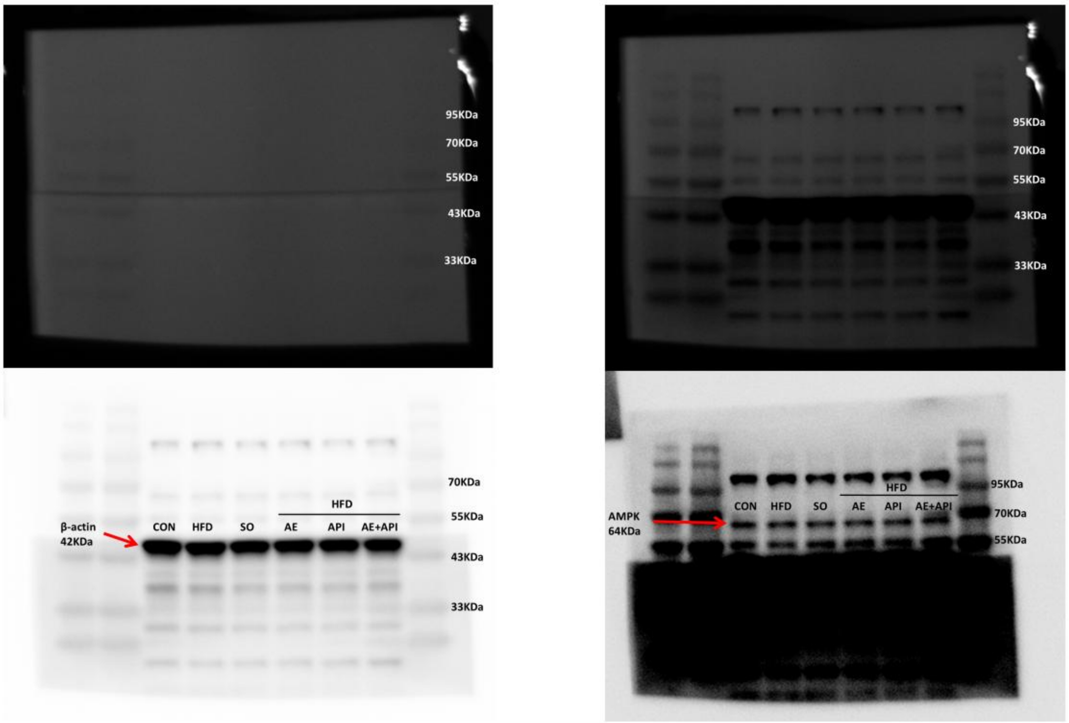

Supplementary Figure S13： Full-length blot image of AMPK (Replicate 1). Red arrows indicate the target protein and GAPDH bands. Molecular weight markers are indicated in the image.

AMPK (2)

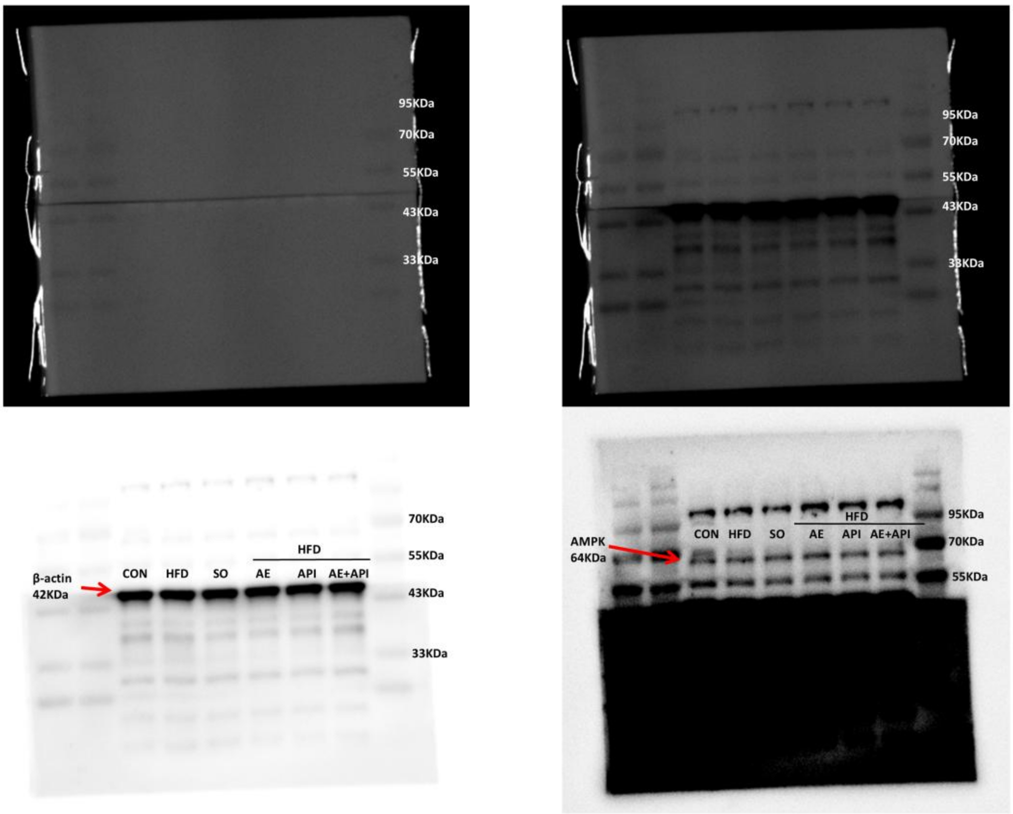

Supplementary Figure S14： Full-length blot image of AMPK (Replicate 2). Red arrows indicate the target protein and GAPDH bands. Molecular weight markers are indicated in the image.

AMPK (3)

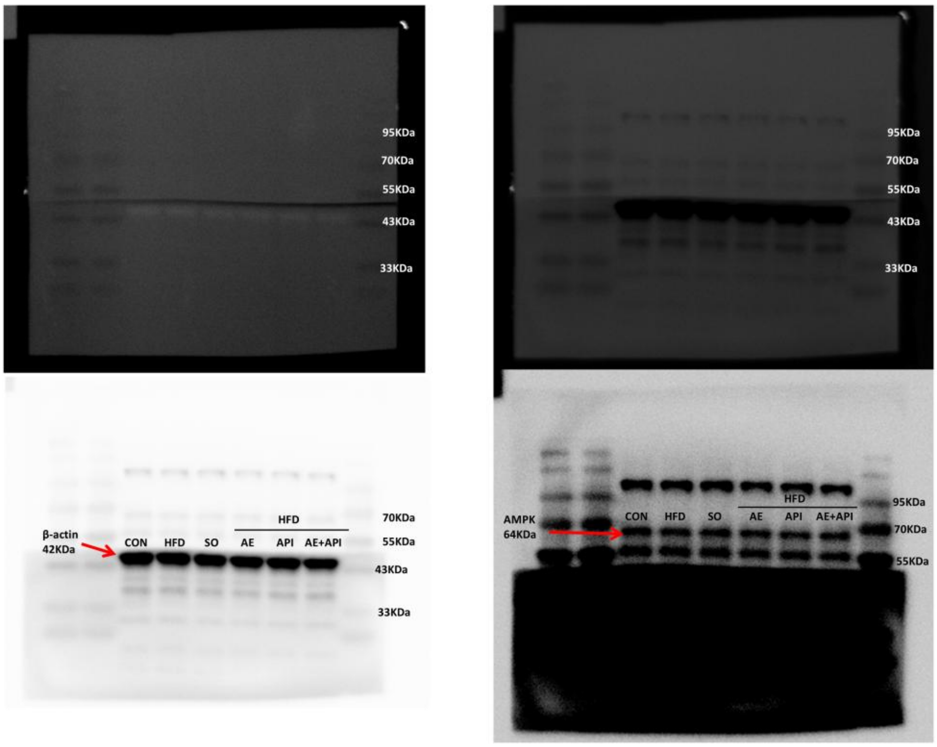

Supplementary Figure S15： Full-length blot image of AMPK (Replicate 3). Red arrows indicate the target protein and GAPDH bands. Molecular weight markers are indicated in the image.

p-AMPK (1)

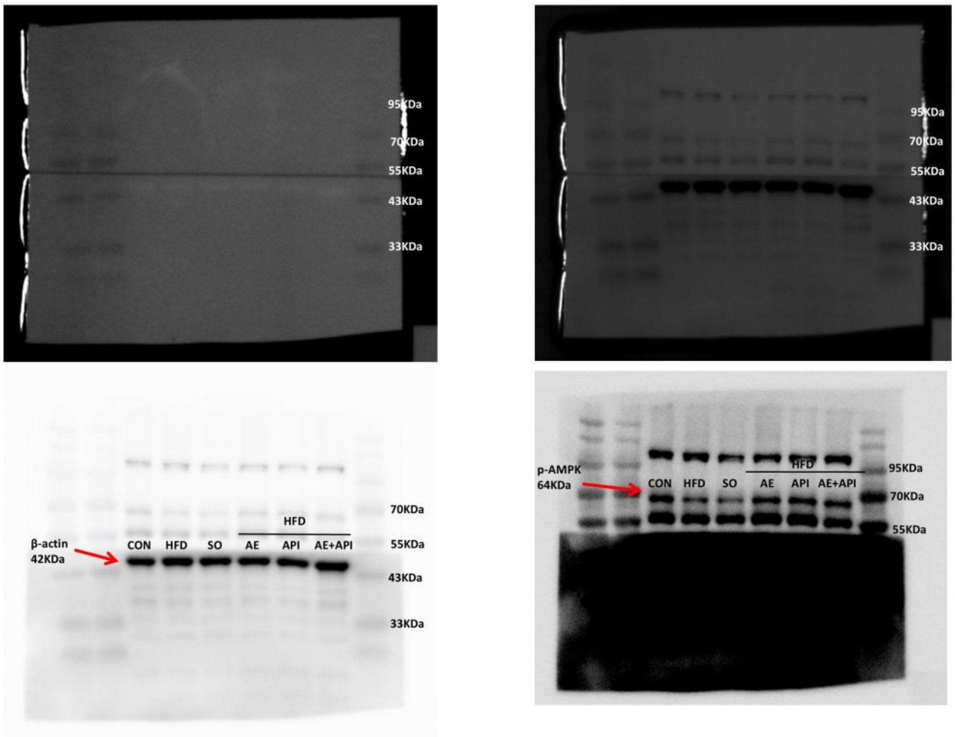

Supplementary Figure S16： Full-length blot image of p- AMPK (Replicate 1). Red arrows indicate the target protein and GAPDH bands. Molecular weight markers are indicated in the image.

p-AMPK (2)

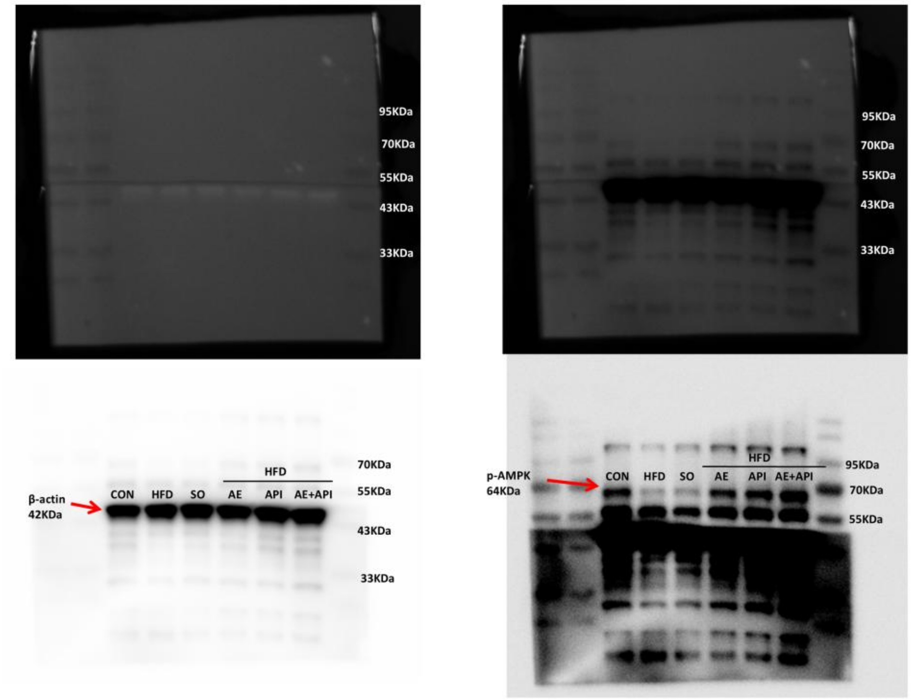

Supplementary Figure S17： **Full-length blot image of p- AMPK (Replicate 2). Red arrows indicate the target protein and GAPDH bands. Molecular weight markers are indicated in the image.**

p-AMPK (3)

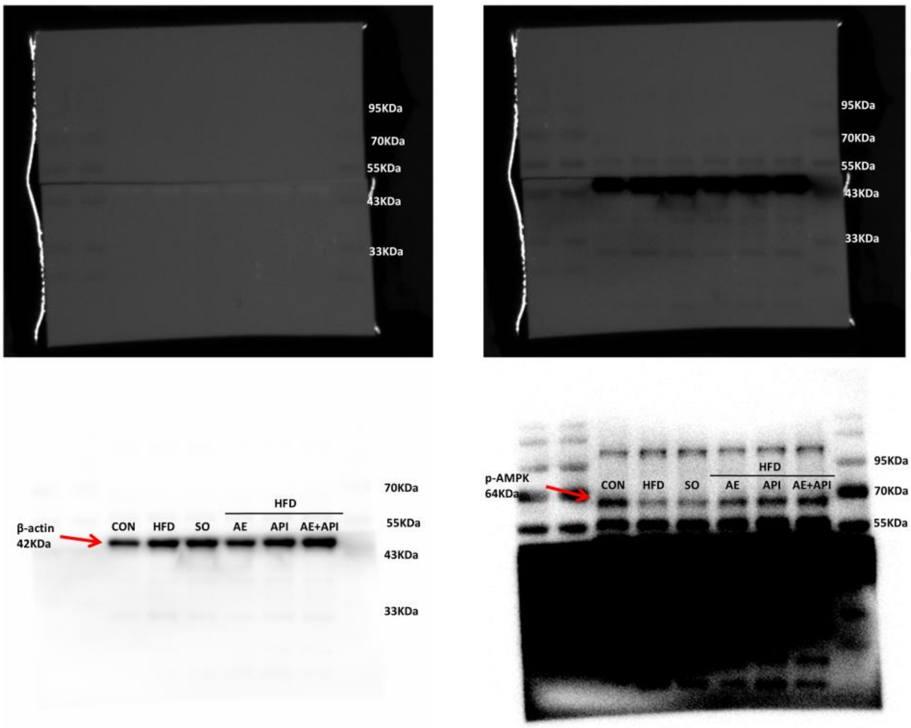

Supplementary Figure S18： **Full-length blot image of p- AMPK (Replicate 3). Red arrows indicate the target protein and GAPDH bands. Molecular weight markers are indicated in the image.**

SIRT1（1）

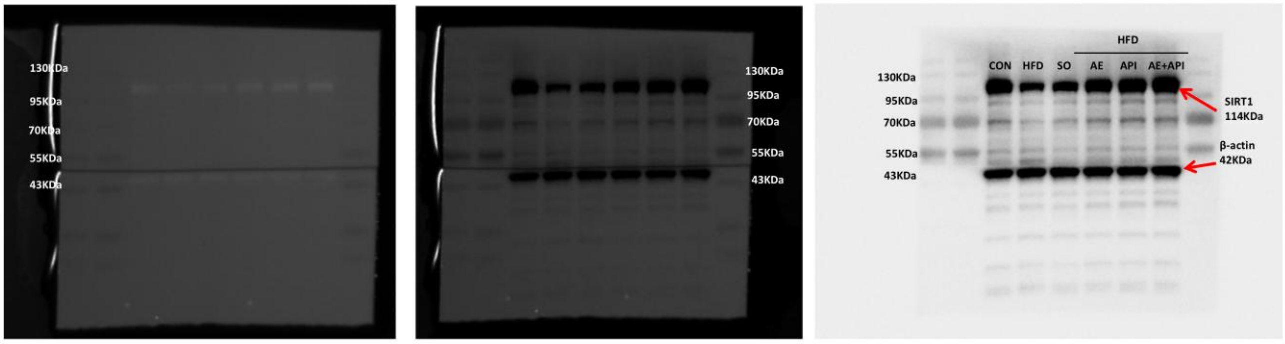

Supplementary Figure S19： Full-length blot image of SIRT1 (Replicate 1). Red arrows indicate the target protein and GAPDH bands. Molecular weight markers are indicated in the image.

SIRT1（2）

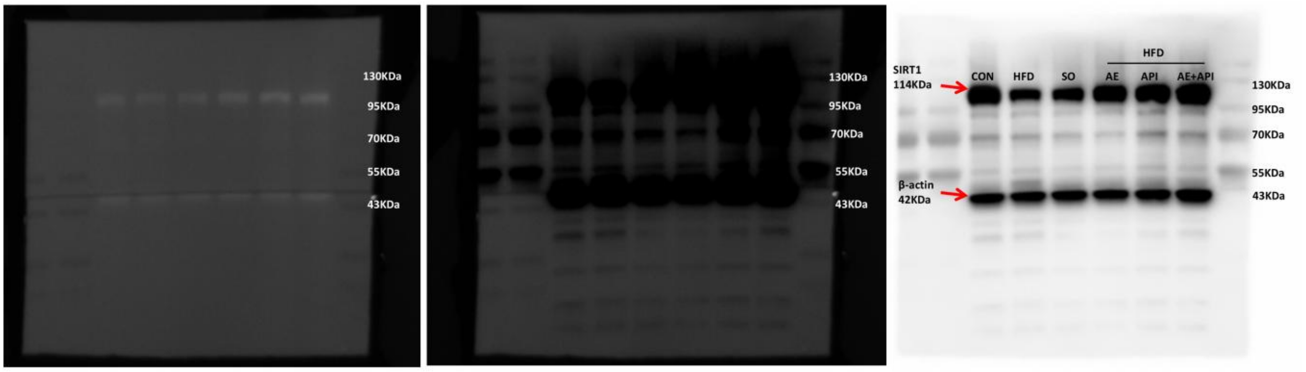

Supplementary Figure S20： Full-length blot image of SIRT1 (Replicate 2). Red arrows indicate the target protein and GAPDH bands. Molecular weight markers are indicated in the image.

SIRT1（3）

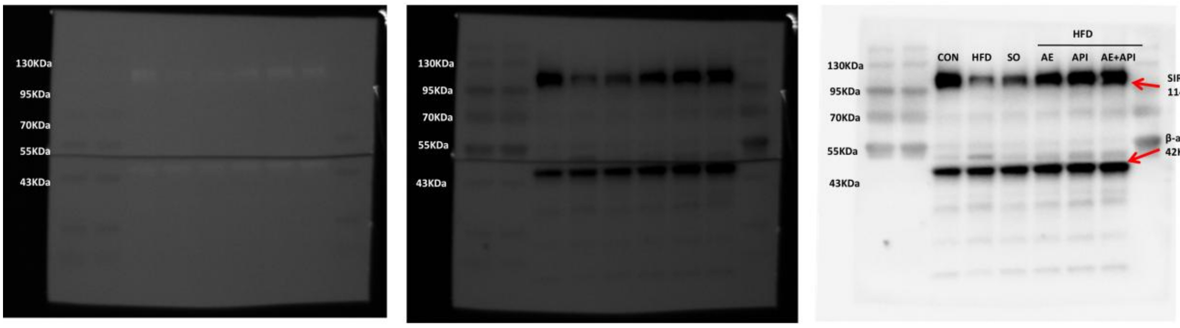

Supplementary Figure S21： Full-length blot image of SIRT1 (Replicate 3). Red arrows indicate the target protein and GAPDH bands. Molecular weight markers are indicated in the image.

PGC-1α（1）

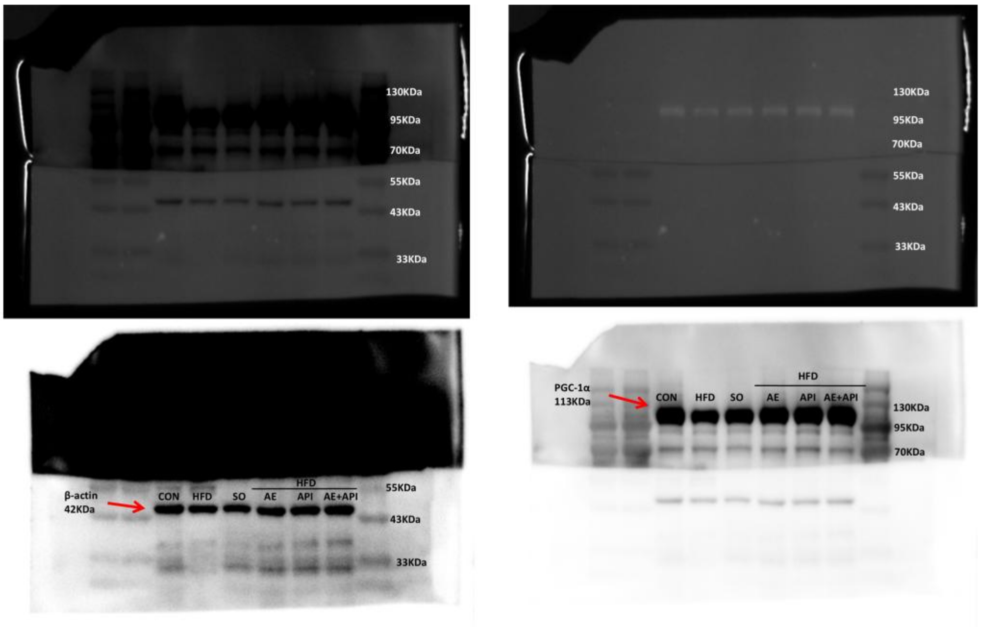

Supplementary Figure S22： Full-length blot image of PGC-1α (Replicate 1). Red arrows indicate the target protein and GAPDH bands. Molecular weight markers are indicated in the image.

PGC-1α（2）

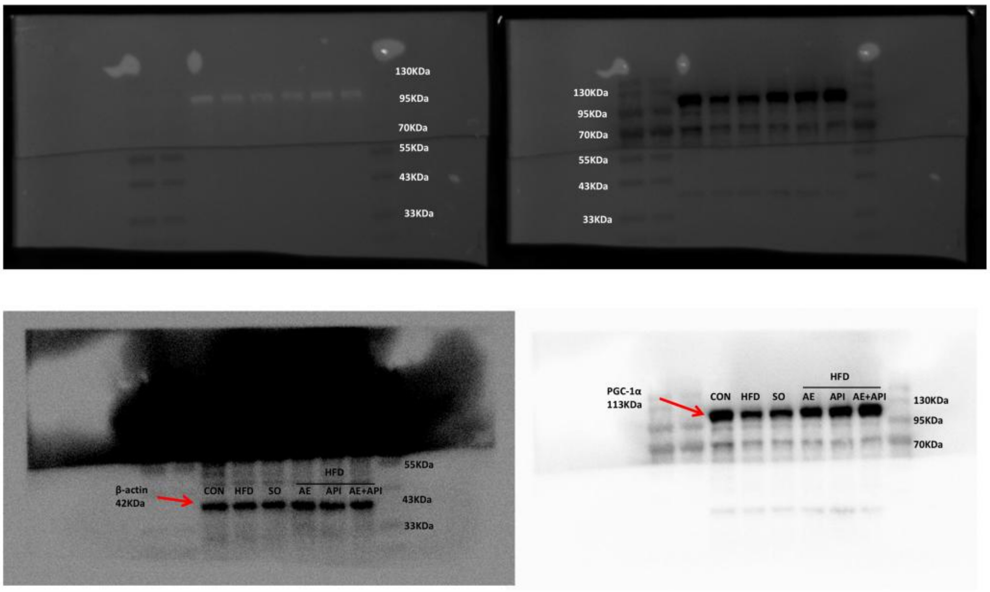

Supplementary Figure S23： Full-length blot image of PGC-1α (Replicate 2). Red arrows indicate the target protein and GAPDH bands. Molecular weight markers are indicated in the image.

PGC-1α（3）

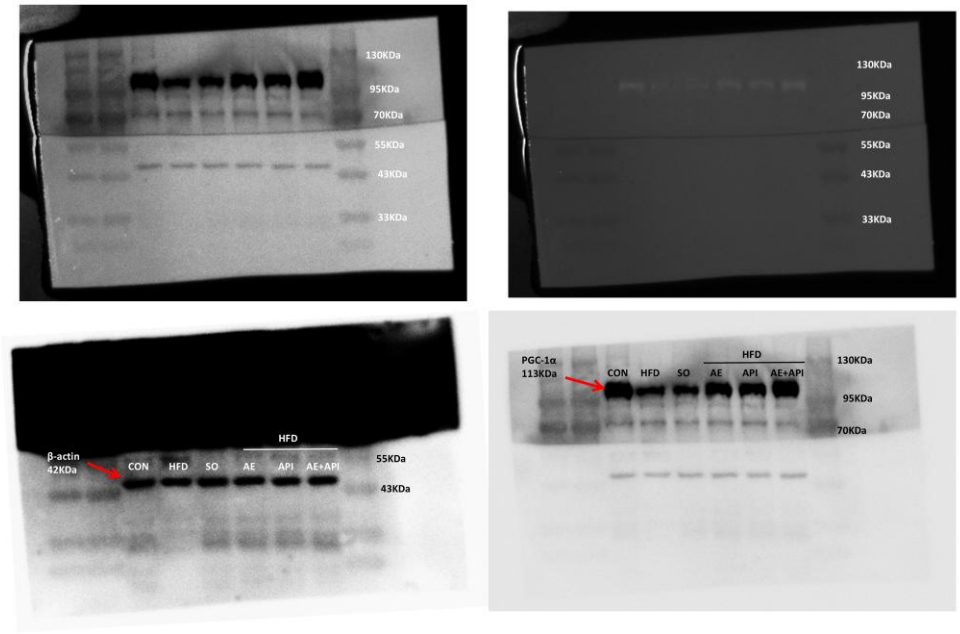

Supplementary Figure S24： Full-length blot image of PGC-1α (Replicate 3). Red arrows indicate the target protein and GAPDH bands. Molecular weight markers are indicated in the image.
