# Supplementary material for: Apigenin Combined with Aerobic Exercise Is Associated with Attenuated Renal Oxidative Stress and AMPK/SIRT1/PGC-1α Pathway Activation in NAFLD Mice
Source: Curr Issues Mol Biol. 2026 Sep 15;48(9):942. doi: 10.3390/cimb48090942 (PMC13605681; doi:10.3390/cimb48090942)
Supplement: Supplementary file 1 [file cimb-48-00942-s001.zip › Supplementary Files/Supplementary Tables/Supplementary Table S1. Complete statistical analysis of biochemical indicators.pdf]

Indicator	Module	Group/Test item	n/DF	Value (Mean ± SD / Statistic)	P value	Conclusion / Significance
**SCr**	1. Descriptive statistics & Shapiro-Wilk normality test	CON	3	35.12 ± 2.568Shapiro-Wilk W = 0.9994	0.9552	Normally distributed
		HFD	3	62.89 ± 4.195Shapiro-Wilk W = 0.8123	0.1443	Normally distributed
		SO	3	58.91 ± 2.779Shapiro-Wilk W = 0.9418	0.5348	Normally distributed
		HFD+AE	3	45.89 ± 2.358Shapiro-Wilk W = 0.8883	0.3492	Normally distributed
		HFD+API	3	46.26 ± 2.320Shapiro-Wilk W = 0.8929	0.3632	Normally distributed
		HFD+AE+API	3	37.58 ± 1.769Shapiro-Wilk W = 0.9993	0.9506	Normally distributed
	2.Homogeneity of variance test & one-way ANOVA	Brown-Forsythe test	5, 12	F = 0.1335	0.98	Equal variances across groups;Welch/Dunnett's T3 not required
		One-way ANOVA (overall)	5, 12	F = 48.73	< 0.001	Significant difference among 6 groups
	3. Tukey's post-hoc multiple comparisons	CON vs. HFD	-	Mean difference = −27.77	< 0.001	***
		HFD vs. SO	-	Mean difference = 3.985	0.52	ns
		HFD vs. HFD+AE	-	Mean difference = 17.00	< 0.001	***
		HFD vs. HFD+API	-	Mean difference = 16.63	< 0.001	***

Indicator	Module	Group/Test item	n/DF	Value (Mean ± SD / Statistic)	P value	Conclusion / Significance
		HFD vs. HFD+AE+API	-	Mean difference = 25.32	< 0.001	***
		HFD+AE vs. HFD+AE+API	-	Mean difference = 8.318	0.03	*
		HFD+API vs. HFD+AE+API	-	Mean difference = 8.683	0.02	*
**BUN**	1. Descriptive statistics & Shapiro-Wilk normality test	CON	3	7.946 ± 0.1693Shapiro-Wilk W = 0.9884	0.7935	Normally distributed
		HFD	3	24.52 ± 0.5272Shapiro-Wilk W = 0.9953	0.8688	Normally distributed
		SO	3	24.25 ± 1.531Shapiro-Wilk W = 0.9949	0.8630	Normally distributed
		HFD+AE	3	15.61 ± 1.206Shapiro-Wilk W = 0.9305	0.4905	Normally distributed
		HFD+API	3	14.53 ± 0.8695Shapiro-Wilk W = 0.8818	0.3297	Normally distributed
		HFD+AE+API	3	11.69 ± 0.9745Shapiro-Wilk W = 0.9140	0.4316	Normally distributed
	2.Homogeneity of variance test & one-way ANOVA	Brown-Forsythe test	5, 12	F = 0.6720	0.65	Equal variances across groups;Welch/Dunnett's T3 not required
		One-way ANOVA (overall)	5, 12	F = 139.7	< 0.001	Significant difference among 6 groups
	3. Tukey's post-hoc multiple comparisons	CON vs. HFD	-	Mean difference = −16.58	< 0.001	***

Indicator	Module	Group/Test item	n/DF	Value (Mean ± SD / Statistic)	P value	Conclusion / Significance
		HFD vs. SO	-	Mean difference = 0.2683	> 0.99	ns
		HFD vs. HFD+AE	-	Mean difference = 8.914	< 0.001	***
		HFD vs. HFD+API	-	Mean difference = 9.988	< 0.001	***
		HFD vs. HFD+AE+API	-	Mean difference = 12.83	< 0.001	***
		HFD+AE vs. HFD+AE+API	-	Mean difference = 3.919	0.004	**
		HFD+API vs. HFD+AE+API	-	Mean difference = 2.845	0.04	*
**TC**	1. Descriptive statistics & Shapiro-Wilk normality test	CON	3	0.0638 ± 0.0066Shapiro-Wilk W = 0.9374	0.5171	Normally distributed
		HFD	3	0.3232 ± 0.0509Shapiro-Wilk W = 0.9840	0.7580	Normally distributed
		SO	3	0.3047 ± 0.0187Shapiro-Wilk W = 0.9987	0.9308	Normally distributed
		HFD+AE	3	0.1655 ± 0.0276Shapiro-Wilk W = 0.9763	0.7048	Normally distributed
		HFD+API	3	0.1556 ± 0.0299Shapiro-Wilk W = 0.8497	0.2396	Normally distributed
		HFD+AE+API	3	0.1092 ± 0.0261Shapiro-Wilk W = 0.7809	0.0697	Normally distributed
	2. Homogeneity of variance test &	Brown-Forsythe test	5, 12	F = 0.6229	0.69	Equal variances across groups;Welch/Dunnett'

Indicator	Module	Group/Test item	n/DF	Value (Mean ± SD / Statistic)	P value	Conclusion / Significance
	one-way ANOVA					s T3 not required
		One-way ANOVA (overall)	5, 12	F = 37.22	< 0.001	Significant difference among 6 groups
	3. Tukey's post-hoc multiple comparisons	CON vs. HFD	-	Mean difference = −0.2594	< 0.001	***
		HFD vs. SO	-	Mean difference = 0.0185	0.97	ns
		HFD vs. HFD+AE	-	Mean difference = 0.1577	< 0.001	***
		HFD vs. HFD+API	-	Mean difference = 0.1677	< 0.001	***
		HFD vs. HFD+AE+API	-	Mean difference = 0.2140	< 0.001	***
		HFD+AE vs. HFD+AE+API	-	Mean difference = 0.0563	0.26	ns
		HFD+API vs. HFD+AE+API	-	Mean difference = 0.0464	0.44	ns
**TG**	1. Descriptive statistics & Shapiro-Wilk normality test	CON	3	0.0202 ± 0.0010Shapiro-Wilk W = 0.9951	0.8665	Normally distributed
		HFD	3	0.2257 ± 0.0077Shapiro-Wilk W = 0.8913	0.3584	Normally distributed
		SO	3	0.1666 ± 0.0024Shapiro-Wilk W = 0.9794	0.7248	Normally distributed
		HFD+AE	3	0.0760 ± 0.0042Shapiro-Wilk W = 0.7500	0.0437	Not normally distributed

Indicator	Module	Group/Test item	n/DF	Value (Mean ± SD / Statistic)	P value	Conclusion / Significance
		HFD+API	3	0.0741 ± 0.0028Shapiro-Wilk W = 0.7695	< 0.05	Not normally distributed
		HFD+AE+API	3	0.0556 ± 0.0009Shapiro-Wilk W = 0.8842	0.3369	Normally distributed
	2. Homogeneity of variance test & one-way ANOVA	Brown-Forsythe test	5, 12	F = 0.6794	0.65	Equal variances across groups;Welch/Dunnett's T3 not required
		One-way ANOVA (overall)	5, 12	F = 1171	< 0.001	Significant difference among 6 groups
	3. Tukey's post-hoc multiple comparisons	CON vs. HFD	-	Mean difference = −0.2055	< 0.001	***
		HFD vs. SO	-	Mean difference = 0.0591	< 0.001	***
		HFD vs. HFD+AE	-	Mean difference = 0.1497	< 0.001	***
		HFD vs. HFD+API	-	Mean difference = 0.1516	< 0.001	***
		HFD vs. HFD+AE+API	-	Mean difference = 0.1701	< 0.001	***
		HFD+AE vs. HFD+AE+API	-	Mean difference = 0.0204	< 0.001	***
		HFD+API vs. HFD+AE+API	-	Mean difference = 0.0185	< 0.001	***
**FFA**	1. Descriptive statistics & Shapiro-Wilk normality test	CON	3	0.0088 ± 0.0007Shapiro-Wilk W = 0.9519	0.5776	Normally distributed
		HFD	3	0.0373 ± 0.0006Shapiro-Wilk	0.2705	Normally distributed

Indicator	Module	Group/Test item	n/DF	Value (Mean ± SD / Statistic)	P value	Conclusion / Significance
				W = 0.8611		
		SO	3	0.0313 ± 0.0028Shapiro-Wilk W = 0.7576	0.0168	Not normally distributed
		HFD+AE	3	0.0154 ± 0.0013Shapiro-Wilk W = 0.9852	0.7670	Normally distributed
		HFD+API	3	0.0163 ± 0.0012Shapiro-Wilk W = 0.9475	0.5584	Normally distributed
		HFD+AE+API	3	0.0098 ± 0.0013Shapiro-Wilk W = 0.8438	0.2239	Normally distributed
	2. Homogeneity of variance test & one-way ANOVA	Brown-Forsythe test	5, 12	F = 0.3346	0.88	Equal variances across groups;Welch/Dunnett's T3 not required
		One-way ANOVA (overall)	5, 12	F = 179.1	< 0.001	Significant difference among 6 groups
	3. Tukey's post-hoc multiple comparisons	CON vs. HFD	-	Mean difference = −0.0285	< 0.001	***
		HFD vs. SO	-	Mean difference = 0.0060	0.004	**
		HFD vs. HFD+AE	-	Mean difference = 0.0219	< 0.001	***
		HFD vs. HFD+API	-	Mean difference = 0.0209	< 0.001	***
		HFD vs. HFD+AE+API	-	Mean difference = 0.0275	< 0.001	***
		HFD+AE vs. HFD+AE+API	-	Mean difference = 0.0056	0.007	**

Indicator	Module	Group/Test item	n/DF	Value (Mean ± SD / Statistic)	P value	Conclusion / Significance
		HFD+API vs. HFD+AE+API	-	Mean difference = 0.0065	0.002	**
**SOD**	1. Descriptive statistics & Shapiro-Wilk normality test	CON	3	37.47 ± 0.6664Shapiro-Wilk W = 0.8193	0.1615	Normally distributed
		HFD	3	20.04 ± 1.464Shapiro-Wilk W = 0.7854	0.0801	Normally distributed
		SO	3	20.10 ± 1.507Shapiro-Wilk W = 0.8356	0.2026	Normally distributed
		HFD+AE	3	27.40 ± 1.829Shapiro-Wilk W = 0.9457	0.5506	Normally distributed
		HFD+API	3	29.26 ± 1.958Shapiro-Wilk W = 0.8367	0.2056	Normally distributed
		HFD+AE+API	3	34.68 ± 0.7253Shapiro-Wilk W = 0.9494	0.5666	Normally distributed
	2. Homogeneity of variance test & one-way ANOVA	Brown-Forsythe test	5, 12	F = 0.2358	0.94	Equal variances across groups;Welch/Dunnett's T3 not required
		One-way ANOVA (overall)	5, 12	F = 75.02	< 0.001	Significant difference among 6 groups
	3. Tukey's post-hoc multiple comparisons	CON vs. HFD	-	Mean difference = 17.43	< 0.001	***
		HFD vs. SO	-	Mean difference = −0.0532	> 0.99	ns
		HFD vs. HFD+AE	-	Mean difference = −7.354	< 0.001	***

Indicator	Module	Group/Test item	n/DF	Value (Mean ± SD / Statistic)	P value	Conclusion / Significance
		HFD vs. HFD+API	-	Mean difference = −9.221	< 0.001	***
		HFD vs. HFD+AE+API	-	Mean difference = −14.64	< 0.001	***
		HFD+AE vs. HFD+AE+API	-	Mean difference = −7.281	< 0.001	***
		HFD+API vs. HFD+AE+API	-	Mean difference = −5.414	0.006	**
**CAT**	1. Descriptive statistics & Shapiro-Wilk normality test	CON	3	5.122 ± 0.1141Shapiro-Wilk W = 0.9628	0.6295	Normally distributed
		HFD	3	1.248 ± 0.0648Shapiro-Wilk W = 0.9182	0.4462	Normally distributed
		SO	3	1.283 ± 0.0302Shapiro-Wilk W = 0.9867	0.7791	Normally distributed
		HFD+AE	3	3.737 ± 0.0789Shapiro-Wilk W = 0.9669	0.6503	Normally distributed
		HFD+API	3	3.867 ± 0.0755Shapiro-Wilk W = 0.9868	0.7804	Normally distributed
		HFD+AE+API	3	4.578 ± 0.0961Shapiro-Wilk W = 0.9968	0.8923	Normally distributed
	2. Homogeneity of variance test & one-way ANOVA	Brown-Forsythe test	5, 12	F = 0.3959	0.84	Equal variances across groups;Welch/Dunnett's T3 not required
		One-way ANOVA (overall)	5, 12	F = 1259	< 0.001	Significant difference among 6 groups

Indicator	Module	Group/Test item	n/DF	Value (Mean ± SD / Statistic)	P value	Conclusion / Significance
	3. Tukey's post-hoc multiple comparisons	CON vs. HFD	-	Mean difference = 3.874	< 0.001	***
		HFD vs. SO	-	Mean difference = −0.0350	> 0.99	ns
		HFD vs. HFD+AE	-	Mean difference = −2.489	< 0.001	***
		HFD vs. HFD+API	-	Mean difference = −2.619	< 0.001	***
		HFD vs. HFD+AE+API	-	Mean difference = −3.330	< 0.001	***
		HFD+AE vs. HFD+AE+API	-	Mean difference = −0.8409	< 0.001	***
		HFD+API vs. HFD+AE+API	-	Mean difference = −0.7111	< 0.001	***
**GSH**	1. Descriptive statistics & Shapiro-Wilk normality test	CON	3	36.38 ± 0.5381Shapiro-Wilk W = 0.8634	0.2770	Normally distributed
		HFD	3	14.74 ± 1.425Shapiro-Wilk W = 0.8526	0.2473	Normally distributed
		SO	3	15.50 ± 0.7049Shapiro-Wilk W = 0.9111	0.4217	Normally distributed
		HFD+AE	3	28.54 ± 1.189Shapiro-Wilk W = 0.9648	0.6397	Normally distributed
		HFD+API	3	28.24 ± 1.275Shapiro-Wilk W = 0.9967	0.8898	Normally distributed
		HFD+AE+API	3	33.43 ± 1.231Shapiro-Wilk	0.7282	Normally distributed

Indicator	Module	Group/Test item	n/DF	Value (Mean ± SD / Statistic)	P value	Conclusion / Significance
				W = 0.9799		
	2. Homogeneity of variance test & one-way ANOVA	Brown-Forsythe test	5, 12	F = 0.2605	0.93	Equal variances across groups;Welch/Dunnett's T3 not required
		One-way ANOVA (overall)	5, 12	F = 200.7	< 0.001	Significant difference among 6 groups
	3. Tukey's post-hoc multiple comparisons	CON vs. HFD	-	Mean difference = 21.63	< 0.001	***
		HFD vs. SO	-	Mean difference = −0.7642	0.95	ns
		HFD vs. HFD+AE	-	Mean difference = −13.80	< 0.001	***
		HFD vs. HFD+API	-	Mean difference = −13.50	< 0.001	***
		HFD vs. HFD+AE+API	-	Mean difference = −18.69	< 0.001	***
		HFD+AE vs. HFD+AE+API	-	Mean difference = −4.884	0.002	**
		HFD+API vs. HFD+AE+API	-	Mean difference = −5.188	0.001	**
**8-OHdG**	1. Descriptive statistics & Shapiro-Wilk normality test	CON	3	3.778 ± 0.0969Shapiro-Wilk W = 0.8826	0.3322	Normally distributed
		HFD	3	7.559 ± 0.2569Shapiro-Wilk W = 0.9956	0.8729	Normally distributed
		SO	3	7.723 ± 0.3381Shapiro-Wilk W = 0.9688	0.6609	Normally distributed

Indicator	Module	Group/Test item	n/DF	Value (Mean ± SD / Statistic)	P value	Conclusion / Significance
		HFD+AE	3	5.657 ± 0.5767Shapiro-Wilk W = 0.9068	0.4076	Normally distributed
		HFD+API	3	5.817 ± 0.4609Shapiro-Wilk W = 0.9843	0.7601	Normally distributed
		HFD+AE+API	3	4.456 ± 0.4546Shapiro-Wilk W = 0.9605	0.6179	Normally distributed
	2. Homogeneity of variance test & one-way ANOVA	Brown-Forsythe test	5, 12	F = 0.4952	0.77	Equal variances across groups;Welch/Dunnett's T3 not required
		One-way ANOVA (overall)	5, 12	F = 48.56	< 0.001	Significant difference among 6 groups
	3. Tukey's post-hoc multiple comparisons	CON vs. HFD	-	Mean difference = −3.780	< 0.001	***
		HFD vs. SO	-	Mean difference = −0.1645	> 0.99	ns
		HFD vs. HFD+AE	-	Mean difference = 1.901	< 0.001	***
		HFD vs. HFD+API	-	Mean difference = 1.741	0.002	**
		HFD vs. HFD+AE+API	-	Mean difference = 3.102	< 0.001	***
		HFD+AE vs. HFD+AE+API	-	Mean difference = 1.201	0.03	*
		HFD+API vs. HFD+AE+API	-	Mean difference = 1.361	0.01	*
**MDA**	1. Descriptive statistics & Shapiro-Wilk	CON	3	3.290 ± 0.2319Shapiro-Wilk W = 0.9834	0.7536	Normally distributed

Indicator	Module	Group/Test item	n/DF	Value (Mean ± SD / Statistic)	P value	Conclusion / Significance
	normality test					
		HFD	3	8.197 ± 0.2094Shapiro-Wilk W = 0.8870	0.3451	Normally distributed
		SO	3	7.877 ± 0.3263Shapiro-Wilk W = 0.9970	0.8948	Normally distributed
		HFD+AE	3	5.232 ± 0.3238Shapiro-Wilk W = 0.9433	0.5408	Normally distributed
		HFD+API	3	5.490 ± 0.1971Shapiro-Wilk W = 0.9887	0.7967	Normally distributed
		HFD+AE+API	3	4.061 ± 0.2874Shapiro-Wilk W = 0.8568	0.2587	Normally distributed
	2. Homogeneity of variance test & one-way ANOVA	Brown-Forsythe test	5, 12	F = 0.1237	0.98	Equal variances across groups;Welch/Dunnett's T3 not required
		One-way ANOVA (overall)	5, 12	F = 165.0	< 0.001	Significant difference among 6 groups
	3. Tukey's post-hoc multiple comparisons	CON vs. HFD	-	Mean difference = −4.908	< 0.001	***
		HFD vs. SO	-	Mean difference = 0.3206	0.69	ns
		HFD vs. HFD+AE	-	Mean difference = 2.965	< 0.001	***
		HFD vs. HFD+API	-	Mean difference = 2.708	< 0.001	***
		HFD vs. HFD+AE+API	-	Mean difference = 4.137	< 0.001	***

Indicator	Module	Group/Test item	n/DF	Value (Mean ± SD / Statistic)	P value	Conclusion / Significance
		HFD+AE vs. HFD+AE+API	-	Mean difference = 1.171	0.002	**
		HFD+API vs. HFD+AE+API	-	Mean difference = 1.429	< 0.001	***
**IL-6**	1. Descriptive statistics & Shapiro-Wilk normality test	CON	3	39.23 ± 2.033Shapiro-Wilk W = 0.8655	0.2828	Normally distributed
		HFD	3	73.31 ± 2.076Shapiro-Wilk W = 0.9529	0.5820	Normally distributed
		SO	3	74.11 ± 1.792Shapiro-Wilk W = 0.9516	0.5766	Normally distributed
		HFD+AE	3	59.98 ± 4.364Shapiro-Wilk W = 0.8809	0.3269	Normally distributed
		HFD+API	3	64.56 ± 1.994Shapiro-Wilk W = 0.9660	0.6456	Normally distributed
		HFD+AE+API	3	39.61 ± 4.167Shapiro-Wilk W = 0.9972	0.8981	Normally distributed
	2. Homogeneity of variance test & one-way ANOVA	Brown-Forsythe test	5, 12	F = 0.4027	0.84	Equal variances across groups;Welch/Dunnett's T3 not required
		One-way ANOVA (overall)	5, 12	F = 85.08	< 0.001	Significant difference among 6 groups
	3. Tukey's post-hoc multiple comparisons	CON vs. HFD	-	Mean difference = −34.08	< 0.001	***
		HFD vs. SO	-	Mean difference = −0.7987	> 0.99	ns

Indicator	Module	Group/Test item	n/DF	Value (Mean ± SD / Statistic)	P value	Conclusion / Significance
		HFD vs. HFD+AE	-	Mean difference = 13.32	0.001	**
		HFD vs. HFD+API	-	Mean difference = 8.746	0.03	*
		HFD vs. HFD+AE+API	-	Mean difference = 33.70	< 0.001	***
		HFD+AE vs. HFD+AE+API	-	Mean difference = 20.38	< 0.001	***
		HFD+API vs. HFD+AE+API	-	Mean difference = 24.95	< 0.001	***
**TNF-α**	1. Descriptive statistics & Shapiro-Wilk normality test	CON	3	49.23 ± 2.025Shapiro-Wilk W = 0.8675	0.2884	Normally distributed
		HFD	3	91.97 ± 4.035Shapiro-Wilk W = 0.9699	0.6669	Normally distributed
		SO	3	87.44 ± 4.096Shapiro-Wilk W = 0.8531	0.2487	Normally distributed
		HFD+AE	3	67.32 ± 4.112Shapiro-Wilk W = 0.9927	0.8365	Normally distributed
		HFD+API	3	63.91 ± 1.802Shapiro-Wilk W = 0.8381	0.2090	Normally distributed
		HFD+AE+API	3	52.95 ± 4.213Shapiro-Wilk W = 0.9537	0.5856	Normally distributed
	2. Homogeneity of variance test & one-way ANOVA	Brown-Forsythe test	5, 12	F = 0.2799	0.92	Equal variances across groups;Welch/Dunnett's T3 not required
		One-way	5, 12	F = 74.08	< 0.001	Significant difference

Indicator	Module	Group/Test item	n/DF	Value (Mean ± SD / Statistic)	P value	Conclusion / Significance
		ANOVA (overall)				among 6 groups
	3. Tukey's post-hoc multiple comparisons	CON vs. HFD	-	Mean difference = −42.74	< 0.001	***
		HFD vs. SO	-	Mean difference = 4.531	0.63	ns
		HFD vs. HFD+AE	-	Mean difference = 24.65	< 0.001	***
		HFD vs. HFD+API	-	Mean difference = 28.06	< 0.001	***
		HFD vs. HFD+AE+API	-	Mean difference = 39.03	< 0.001	***
		HFD+AE vs. HFD+AE+API	-	Mean difference = 14.37	0.003	**
		HFD+API vs. HFD+AE+API	-	Mean difference = 10.96	0.02	*
