# Supplementary material for: Apigenin Combined with Aerobic Exercise Is Associated with Attenuated Renal Oxidative Stress and AMPK/SIRT1/PGC-1α Pathway Activation in NAFLD Mice
Source: Curr Issues Mol Biol. 2026 Sep 15;48(9):942. doi: 10.3390/cimb48090942 (PMC13605681; doi:10.3390/cimb48090942)
Supplement: Supplementary file 1 [file cimb-48-00942-s001.zip › Supplementary Files/Supplementary Tables/Supplementary Table S2. Complete statistical analysis of qPCR gene expression indicators.pdf]

Indicator	Module	Group/Test item	n/DF	Value (Mean ± SD / Statistic)	P value	Conclusion / Significance
Sod1	**1.Descriptive statistics & Shapiro-Wilk normality test**	CON	3	1.000 ± 0.000; Shapiro-Wilk W = Invalid input data	—	—
		HFD	3	0.3415 ± 0.0388; Shapiro-Wilk W = 0.9982	0.9200	Normally distributed
		SO	3	0.3728 ± 0.0404; Shapiro-Wilk W = 0.7975	0.1086	Normally distributed
		HFD+AE	3	0.5491 ± 0.0176; Shapiro-Wilk W = 0.8726	0.3029	Normally distributed
		HFD+API	3	0.5590 ± 0.0286; Shapiro-Wilk W = 0.8455	0.2285	Normally distributed
		HFD+AE+API	3	0.7554 ± 0.0120; Shapiro-Wilk W = 0.7996	0.1135	Normally distributed
	**2.Homogeneity of variance test & one-way ANOVA**	Brown-Forsythe test	5, 12	F = 0.6320	0.68	Equal variances across groups; Welch/Dunnett's T3 not required
		One-way ANOVA (overall)	5, 12	F = 250.5	< 0.001	Significant difference among 6 groups
	**3.Tukey's post-hoc multiple comparisons**	CON vs. HFD		Mean difference = 0.6585	< 0.001	***
		HFD vs. SO		Mean difference = −0.03128	0.72	ns
		HFD vs. HFD+AE		Mean difference = −0.2076	< 0.001	***
		HFD vs. HFD+API		Mean difference = −0.2175	< 0.001	***
		HFD vs.		Mean difference =	< 0.001	***

Indicator	Module	Group/Test item	n/DF	Value (Mean ± SD / Statistic)	P value	Conclusion / Significance
		HFD+AE+API		−0.4139		
		HFD+AE vs. HFD+AE+API		Mean difference = −0.2063	< 0.001	***
		HFD+API vs. HFD+AE+API		Mean difference = −0.1964	< 0.001	***
Cat	**1.Descriptive statistics & Shapiro-Wilk normality test**	CON	3	1.000 ± 0.000; Shapiro-Wilk W = Invalid input data	—	—
		HFD	3	0.3308 ± 0.0203; Shapiro-Wilk W = 0.9835	0.7536	Normally distributed
		SO	3	0.3364 ± 0.0168; Shapiro-Wilk W = 0.9790	0.7219	Normally distributed
		HFD+AE	3	0.5787 ± 0.0331; Shapiro-Wilk W = 0.9709	0.6726	Normally distributed
		HFD+API	3	0.5653 ± 0.0261; Shapiro-Wilk W = 1.000	0.9923	Normally distributed
		HFD+AE+API	3	0.6913 ± 0.0276; Shapiro-Wilk W = 0.9992	0.9453	Normally distributed
	**2.Homogeneity of variance test & one-way ANOVA**	Brown-Forsythe test	5, 12	F = 0.8876	0.52	Equal variances across groups; Welch/Dunnett's T3 not required
		One-way ANOVA (overall)	5, 12	F = 345.1	< 0.001	Significant difference among 6 groups
	**3.Tukey's post-hoc multiple comparisons**	CON vs. HFD		Mean difference = 0.6692	< 0.001	***
		HFD vs. SO		Mean difference = −0.005606	> 0.99	ns

Indicator	Module	Group/Test item	n/DF	Value (Mean ± SD / Statistic)	P value	Conclusion / Significance
		HFD vs. HFD+AE		Mean difference = −0.2480	< 0.001	***
		HFD vs. HFD+API		Mean difference = −0.2345	< 0.001	***
		HFD vs. HFD+AE+API		Mean difference = −0.3605	< 0.001	***
		HFD+AE vs. HFD+AE+API		Mean difference = −0.1125	< 0.001	***
		HFD+API vs. HFD+AE+API		Mean difference = −0.1260	< 0.001	***
Gpx1	**1.Descriptive statistics & Shapiro-Wilk normality test**	CON	3	1.000 ± 0.000; Shapiro-Wilk W = Invalid input data	—	—
		HFD	3	0.2255 ± 0.0389; Shapiro-Wilk W = 0.8509	0.2429	Normally distributed
		SO	3	0.1685 ± 0.0191; Shapiro-Wilk W = 0.9987	0.9315	Normally distributed
		HFD+AE	3	0.6691 ± 0.0197; Shapiro-Wilk W = 0.9984	0.9233	Normally distributed
		HFD+API	3	0.6379 ± 0.0205; Shapiro-Wilk W = 0.9998	0.9721	Normally distributed
		HFD+AE+API	3	0.7725 ± 0.0249; Shapiro-Wilk W = 0.8341	0.1988	Normally distributed
	**2.Homogeneity of variance test & one-way ANOVA**	Brown-Forsythe test	5, 12	F = 0.5336	0.75	Equal variances across groups; Welch/Dunnett's T3 not required
		One-way ANOVA	5, 12	F = 567.0	< 0.001	Significant difference among 6 groups

Indicator	Module	Group/Test item	n/DF	Value (Mean ± SD / Statistic)	P value	Conclusion / Significance
		(overall)				
	**3.Tukey's post-hoc multiple comparisons**	CON vs. HFD		Mean difference = 0.7745	< 0.001	***
		HFD vs. SO		Mean difference = 0.05698	0.09	ns
		HFD vs. HFD+AE		Mean difference = −0.4436	< 0.001	***
		HFD vs. HFD+API		Mean difference = −0.4124	< 0.001	***
		HFD vs. HFD+AE+API		Mean difference = −0.5470	< 0.001	***
		HFD+AE vs. HFD+AE+API		Mean difference = −0.1034	0.002	**
		HFD+API vs. HFD+AE+API		Mean difference = −0.1346	< 0.001	***
Il6	**1.Descriptive statistics & Shapiro-Wilk normality test**	CON	3	1.000 ± 0.000; Shapiro-Wilk W = Invalid input data	—	—
		HFD	3	2.306 ± 0.1143; Shapiro-Wilk W = 0.9464	0.5539	Normally distributed
		SO	3	2.168 ± 0.2343; Shapiro-Wilk W = 0.8332	0.1965	Normally distributed
		HFD+AE	3	1.654 ± 0.0956; Shapiro-Wilk W = 0.9729	0.6844	Normally distributed
		HFD+API	3	1.559 ± 0.0744; Shapiro-Wilk W = 0.9769	0.7086	Normally distributed
		HFD+AE+API	3	1.252 ± 0.1170; Shapiro-Wilk W =	0.6435	Normally distributed

Indicator	Module	Group/Test item	n/DF	Value (Mean ± SD / Statistic)	P value	Conclusion / Significance
				0.9656		
	**2.Homogeneity of variance test & one-way ANOVA**	Brown-Forsythe test	5, 12	F = 0.6342	0.68	Equal variances across groups; Welch/Dunnett's T3 not required
		One-way ANOVA (overall)	5, 12	F = 48.09	< 0.001	Significant difference among 6 groups
	**3.Tukey's post-hoc multiple comparisons**	CON vs. HFD		Mean difference = −1.306	< 0.001	***
		HFD vs. SO		Mean difference = 0.1382	0.76	ns
		HFD vs. HFD+AE		Mean difference = 0.6513	< 0.001	***
		HFD vs. HFD+API		Mean difference = 0.7466	< 0.001	***
		HFD vs. HFD+AE+API		Mean difference = 1.054	< 0.001	***
		HFD+AE vs. HFD+AE+API		Mean difference = 0.4027	0.02	*
		HFD+API vs. HFD+AE+API		Mean difference = 0.3075	0.09	ns
Tnf	**1.Descriptive statistics & Shapiro-Wilk normality test**	CON	3	1.000 ± 0.000; Shapiro-Wilk W = Invalid input data	—	—
		HFD	3	4.150 ± 0.2018; Shapiro-Wilk W = 0.9569	0.6004	Normally distributed
		SO	3	3.853 ± 0.1841; Shapiro-Wilk W = 0.9679	0.6559	Normally distributed
		HFD+AE	3	2.489 ± 0.3189; Shapiro-Wilk W =	0.4208	Normally distributed

Indicator	Module	Group/Test item	n/DF	Value (Mean ± SD / Statistic)	P value	Conclusion / Significance
				0.9108		
		HFD+API	3	2.370 ± 0.0877; Shapiro-Wilk W = 0.9569	0.6005	Normally distributed
		HFD+AE+API	3	1.319 ± 0.1465; Shapiro-Wilk W = 0.9972	0.8989	Normally distributed
	**2.Homogeneity of variance test & one-way ANOVA**	Brown-Forsythe test	5, 12	F = 0.8471	0.54	Equal variances across groups; Welch/Dunnett's T3 not required
		One-way ANOVA (overall)	5, 12	F = 143.8	< 0.001	Significant difference among 6 groups
	**3.Tukey's post-hoc multiple comparisons**	CON vs. HFD		Mean difference = −3.150	< 0.001	***
		HFD vs. SO		Mean difference = 0.2969	0.41	ns
		HFD vs. HFD+AE		Mean difference = 1.661	< 0.001	***
		HFD vs. HFD+API		Mean difference = 1.780	< 0.001	***
		HFD vs. HFD+AE+API		Mean difference = 2.831	< 0.001	***
		HFD+AE vs. HFD+AE+API		Mean difference = 1.170	< 0.001	***
		HFD+API vs. HFD+AE+API		Mean difference = 1.051	< 0.001	***
Havcr1	**1.Descriptive statistics & Shapiro-Wilk normality test**	CON	3	1.000 ± 0.000; Shapiro-Wilk W = Invalid input data	—	—
		HFD	3	3.903 ± 0.2006; Shapiro-Wilk W =	0.5473	Normally distributed

Indicator	Module	Group/Test item	n/DF	Value (Mean ± SD / Statistic)	P value	Conclusion / Significance
				0.9449		
		SO	3	3.474 ± 0.2556; Shapiro-Wilk W = 0.9968	0.8919	Normally distributed
		HFD+AE	3	1.907 ± 0.1579; Shapiro-Wilk W = 0.9976	0.9068	Normally distributed
		HFD+API	3	1.719 ± 0.1366; Shapiro-Wilk W = 0.9617	0.6237	Normally distributed
		HFD+AE+API	3	1.153 ± 0.2859; Shapiro-Wilk W = 0.8205	0.1645	Normally distributed
	**2.Homogeneity of variance test & one-way ANOVA**	Brown-Forsythe test	5, 12	F = 0.5917	0.71	Equal variances across groups; Welch/Dunnett's T3 not required
		One-way ANOVA (overall)	5, 12	F = 115.0	< 0.001	Significant difference among 6 groups
	**3.Tukey's post-hoc multiple comparisons**	CON vs. HFD		Mean difference = −2.903	< 0.001	***
		HFD vs. SO		Mean difference = 0.4290	0.15	ns
		HFD vs. HFD+AE		Mean difference = 1.996	< 0.001	***
		HFD vs. HFD+API		Mean difference = 2.185	< 0.001	***
		HFD vs. HFD+AE+API		Mean difference = 2.751	< 0.001	***
		HFD+AE vs. HFD+AE+API		Mean difference = 0.7545	0.005	**
		HFD+API vs.		Mean difference =	0.04	*

Indicator	Module	Group/Test item	n/DF	Value (Mean ± SD / Statistic)	P value	Conclusion / Significance
		HFD+AE+API		0.5661		
Lcn2	**1.Descriptive statistics & Shapiro-Wilk normality test**	CON	3	1.000 ± 0.000; Shapiro-Wilk W = Invalid input data	—	—
		HFD	3	4.672 ± 0.3465; Shapiro-Wilk W = 0.8889	0.3511	Normally distributed
		SO	3	4.390 ± 0.3819; Shapiro-Wilk W = 0.7639	0.0309	Not normally distributed
		HFD+AE	3	2.379 ± 0.3105; Shapiro-Wilk W = 0.8217	0.1675	Normally distributed
		HFD+API	3	1.742 ± 0.1943; Shapiro-Wilk W = 0.9729	0.6844	Normally distributed
		HFD+AE+API	3	1.278 ± 0.0971; Shapiro-Wilk W = 0.9989	0.9363	Normally distributed
	**2.Homogeneity of variance test & one-way ANOVA**	Brown-Forsythe test	5, 12	F = 0.4664	0.79	Equal variances across groups; Welch/Dunnett's T3 not required
		One-way ANOVA (overall)	5, 12	F = 110.6	< 0.001	Significant difference among 6 groups
	**3.Tukey's post-hoc multiple comparisons**	CON vs. HFD		Mean difference = −3.672	< 0.001	***
		HFD vs. SO		Mean difference = 0.2816	0.77	ns
		HFD vs. HFD+AE		Mean difference = 2.293	< 0.001	***
		HFD vs. HFD+API		Mean difference = 2.930	< 0.001	***

Indicator	Module	Group/Test item	n/DF	Value (Mean ± SD / Statistic)	P value	Conclusion / Significance
		HFD vs. HFD+AE+API		Mean difference = 3.393	< 0.001	***
		HFD+AE vs. HFD+AE+API		Mean difference = 1.101	0.002	**
		HFD+API vs. HFD+AE+API		Mean difference = 0.4634	0.32	ns
Fabp1	**1.Descriptive statistics & Shapiro-Wilk normality test**	CON	3	1.000 ± 0.000; Shapiro-Wilk W = Invalid input data	—	—
		HFD	3	3.831 ± 0.0905; Shapiro-Wilk W = 0.9802	0.7307	Normally distributed
		SO	3	3.647 ± 0.3348; Shapiro-Wilk W = 0.9520	0.5779	Normally distributed
		HFD+AE	3	1.882 ± 0.0449; Shapiro-Wilk W = 0.8899	0.3539	Normally distributed
		HFD+API	3	2.366 ± 0.3206; Shapiro-Wilk W = 0.7708	0.0466	Not normally distributed
		HFD+AE+API	3	1.354 ± 0.2127; Shapiro-Wilk W = 0.9999	0.9794	Normally distributed
	**2.Homogeneity of variance test & one-way ANOVA**	Brown-Forsythe test	5, 12	F = 0.8525	0.54	Equal variances across groups; Welch/Dunnett's T3 not required
		One-way ANOVA (overall)	5, 12	F = 92.04	< 0.001	Significant difference among 6 groups
	**3.Tukey's post-hoc multiple comparisons**	CON vs. HFD		Mean difference = −2.831	< 0.001	***
		HFD vs. SO		Mean difference =	0.89	ns

Indicator	Module	Group/Test item	n/DF	Value (Mean ± SD / Statistic)	P value	Conclusion / Significance
				0.1841		
		HFD vs. HFD+AE		Mean difference = 1.949	< 0.001	***
		HFD vs. HFD+API		Mean difference = 1.465	< 0.001	***
		HFD vs. HFD+AE+API		Mean difference = 2.477	< 0.001	***
		HFD+AE vs. HFD+AE+API		Mean difference = 0.5276	0.08	ns
		HFD+API vs. HFD+AE+API		Mean difference = 1.012	< 0.001	***
Prkaa1	**1.Descriptive statistics & Shapiro-Wilk normality test**	CON	3	1.000 ± 0.000; Shapiro-Wilk W = Invalid input data	—	—
		HFD	3	0.3897 ± 0.0304; Shapiro-Wilk W = 0.9987	0.9311	Normally distributed
		SO	3	0.4264 ± 0.0356; Shapiro-Wilk W = 0.9744	0.6933	Normally distributed
		HFD+AE	3	0.9115 ± 0.0271; Shapiro-Wilk W = 0.7825	0.0733	Normally distributed
		HFD+API	3	0.6900 ± 0.0197; Shapiro-Wilk W = 0.9846	0.7621	Normally distributed
		HFD+AE+API	3	1.029 ± 0.0458; Shapiro-Wilk W = 1.000	0.9986	Normally distributed
	**2.Homogeneity of variance test & one-way ANOVA**	Brown-Forsythe test	5, 12	F = 0.8350	0.55	Equal variances across groups; Welch/Dunnett's T3 not required

Indicator	Module	Group/Test item	n/DF	Value (Mean ± SD / Statistic)	P value	Conclusion / Significance
		One-way ANOVA (overall)	5, 12	F = 268.7	< 0.001	Significant difference among 6 groups
	**3.Tukey's post-hoc multiple comparisons**	CON vs. HFD		Mean difference = 0.6103	< 0.001	***
		HFD vs. SO		Mean difference = −0.03673	0.67	ns
		HFD vs. HFD+AE		Mean difference = −0.5218	< 0.001	***
		HFD vs. HFD+API		Mean difference = −0.3003	< 0.001	***
		HFD vs. HFD+AE+API		Mean difference = −0.6390	< 0.001	***
		HFD+AE vs. HFD+AE+API		Mean difference = −0.1172	0.005	**
		HFD+API vs. HFD+AE+API		Mean difference = −0.3387	< 0.001	***
Sirt1	**1.Descriptive statistics & Shapiro-Wilk normality test**	CON	3	1.000 ± 0.000; Shapiro-Wilk W = Invalid input data	—	—
		HFD	3	0.3255 ± 0.0260; Shapiro-Wilk W = 0.9910	0.8185	Normally distributed
		SO	3	0.3452 ± 0.0447; Shapiro-Wilk W = 0.9833	0.7522	Normally distributed
		HFD+AE	3	0.9298 ± 0.0282; Shapiro-Wilk W = 0.9171	0.4420	Normally distributed
		HFD+API	3	0.7700 ± 0.0538; Shapiro-Wilk W = 0.8980	0.3790	Normally distributed

Indicator	Module	Group/Test item	n/DF	Value (Mean ± SD / Statistic)	P value	Conclusion / Significance
		HFD+AE+API	3	1.041 ± 0.0386; Shapiro-Wilk W = 0.8510	0.2432	Normally distributed
	**2.Homogeneity of variance test & one-way ANOVA**	Brown-Forsythe test	5, 12	F = 0.5984	0.70	Equal variances across groups; Welch/Dunnett's T3 not required
		One-way ANOVA (overall)	5, 12	F = 239.7	< 0.001	Significant difference among 6 groups
	**3.Tukey's post-hoc multiple comparisons**	CON vs. HFD		Mean difference = 0.6745	< 0.001	***
		HFD vs. SO		Mean difference = −0.01967	0.98	ns
		HFD vs. HFD+AE		Mean difference = −0.6043	< 0.001	***
		HFD vs. HFD+API		Mean difference = −0.4445	< 0.001	***
		HFD vs. HFD+AE+API		Mean difference = −0.7158	< 0.001	***
		HFD+AE vs. HFD+AE+API		Mean difference = −0.116	0.02	*
		HFD+API vs. HFD+AE+API		Mean difference = −0.2713	< 0.001	***
Ppargc1a	**1.Descriptive statistics & Shapiro-Wilk normality test**	CON	3	1.000 ± 0.000; Shapiro-Wilk W = Invalid input data	—	—
		HFD	3	0.2696 ± 0.0248; Shapiro-Wilk W = 0.9363	0.5128	Normally distributed
		SO	3	0.3540 ± 0.0219; Shapiro-Wilk W = 0.9578	0.6049	Normally distributed

Indicator	Module	Group/Test item	n/DF	Value (Mean ± SD / Statistic)	P value	Conclusion / Significance
		HFD+AE	3	0.9184 ± 0.0367; Shapiro-Wilk W = 0.9822	0.7446	Normally distributed
		HFD+API	3	0.8629 ± 0.0400; Shapiro-Wilk W = 1.000	0.9961	Normally distributed
		HFD+AE+API	3	1.064 ± 0.0922; Shapiro-Wilk W = 0.9475	0.5584	Normally distributed
	**2.Homogeneity of variance test & one-way ANOVA**	Brown-Forsythe test	5, 12	F = 1.302	0.33	Equal variances across groups; Welch/Dunnett's T3 not required
		One-way ANOVA (overall)	5, 12	F = 169.3	< 0.001	Significant difference among 6 groups
	**3.Tukey's post-hoc multiple comparisons**	CON vs. HFD		Mean difference = 0.7304	< 0.001	***
		HFD vs. SO		Mean difference = −0.08441	0.28	ns
		HFD vs. HFD+AE		Mean difference = −0.6488	< 0.001	***
		HFD vs. HFD+API		Mean difference = −0.5933	< 0.001	***
		HFD vs. HFD+AE+API		Mean difference = −0.7943	< 0.001	***
		HFD+AE vs. HFD+AE+API		Mean difference = −0.1455	0.02	*
		HFD+API vs. HFD+AE+API		Mean difference = −0.2010	0.002	**
