# Supplementary material for: Apigenin Combined with Aerobic Exercise Is Associated with Attenuated Renal Oxidative Stress and AMPK/SIRT1/PGC-1α Pathway Activation in NAFLD Mice
Source: Curr Issues Mol Biol. 2026 Sep 15;48(9):942. doi: 10.3390/cimb48090942 (PMC13605681; doi:10.3390/cimb48090942)
Supplement: Supplementary file 1 [file cimb-48-00942-s001.zip › Supplementary Files/Supplementary Tables/Supplementary Table S3. Complete statistical analysis of Western blot protein expression indicators.pdf]

Indicator	Module	Group/Test item	n/DF	Value (Mean ± SD / Statistic)	P value	Conclusion / Significance
**P-AMPK/AMPK**	**1.Descriptive statistics & Shapiro-Wilk normality test**	CON	3	1.265 ± 0.0877Shapiro-Wilk W = 0.9986	0.9289	Normally distributed
		HFD	3	0.5326 ± 0.0437Shapiro-Wilk W = 0.9997	0.9686	Normally distributed
		SO	3	0.5943 ± 0.0189Shapiro-Wilk W = 0.8590	0.2648	Normally distributed
		HFD+AE	3	0.9207 ± 0.0301Shapiro-Wilk W = 0.9166	0.4404	Normally distributed
		HFD+API	3	0.9136 ± 0.0169Shapiro-Wilk W = 0.9978	0.9113	Normally distributed
		HFD+AE+API	3	1.100 ± 0.0613Shapiro-Wilk W = 0.9569	0.6007	Normally distributed
	**2. Homogeneity of variance test & one-way ANOVA**	Brown-Forsythe test	5, 12	F = 1.069	0.42	Equal variances across groups;Welch/Dunnett's T3 not required
		One-way ANOVA (overall)	5, 12	F = 97.04	< 0.001	Significant difference among 6 groups
	**3. Tukey's post-hoc multiple comparisons**	CON vs. HFD	-	Mean difference = 0.7324	< 0.001	***
		HFD vs. SO	-	Mean difference	0.66	ns

Indicator	Module	Group/Test item	n/DF	Value (Mean ± SD / Statistic)	P value	Conclusion / Significance
				= −0.0617		
		HFD vs. HFD+AE	-	Mean difference = −0.3881	< 0.001	***
		HFD vs. HFD+API	-	Mean difference = −0.3810	< 0.001	***
		HFD vs. HFD+AE+API	-	Mean difference = −0.5674	< 0.001	***
		HFD+AE vs. HFD+AE+API	-	Mean difference = −0.1793	0.002	**
		HFD+API vs. HFD+AE+API	-	Mean difference = −0.1864	0.006	**
**SIRT1**	**1. Descriptive statistics & Shapiro-Wilk normality test**	CON	3	0.9864 ± 0.0354Shapiro-Wilk W = 0.8827	0.3325	Normally distributed
		HFD	3	0.4929 ± 0.0474Shapiro-Wilk W = 0.8824	0.3315	Normally distributed
		SO	3	0.5706 ± 0.0319Shapiro-Wilk W = 0.9602	0.6165	Normally distributed
		HFD+AE	3	0.9226 ± 0.0391Shapiro-Wilk W = 0.9238	0.4657	Normally distributed
		HFD+API	3	0.9368 ± 0.0335Shapiro-Wilk W = 0.9204	0.4537	Normally distributed
		HFD+AE+API	3	0.9648 ± 0.0230Shapiro-Wilk W =	0.6207	Normally distributed

Indicator	Module	Group/Test item	n/DF	Value (Mean ± SD / Statistic)	P value	Conclusion / Significance
				0.9611		
	**2. Homogeneity of variance test & one-way ANOVA**	Brown-Forsythe test	5, 12	F = 0.0969	> 0.99	Equal variances across groups;Welch/Dunnett's T3 not required
		One-way ANOVA (overall)	5, 12	F = 113.1	< 0.001	Significant difference among 6 groups
	**3. Tukey's post-hoc multiple comparisons**	CON vs. HFD	-	Mean difference = 0.4936	< 0.001	***
		HFD vs. SO	-	Mean difference = −0.0778	0.16	ns
		HFD vs. HFD+AE	-	Mean difference = −0.4297	< 0.001	***
		HFD vs. HFD+API	-	Mean difference = −0.4440	< 0.001	***
		HFD vs. HFD+AE+API	-	Mean difference = −0.4719	< 0.001	***
		HFD+AE vs. HFD+AE+API	-	Mean difference = −0.0422	0.70	ns
		HFD+API vs. HFD+AE+API	-	Mean difference = −0.0279	0.92	ns
**PGC-1α**	**1. Descriptive statistics & Shapiro-Wilk normality test**	CON	3	1.225 ± 0.0565Shapiro-Wilk W = 0.9126	0.4268	Normally distributed
		HFD	3	0.6207 ± 0.0333Shapiro-Wilk W = 0.9998	0.9748	Normally distributed
		SO	3	0.6433 ± 0.0552Shapiro-	0.5558	Normally distributed

Indicator	Module	Group/Test item	n/DF	Value (Mean ± SD / Statistic)	P value	Conclusion / Significance
				Wilk W = 0.9469		
		HFD+AE	3	1.088 ± 0.0400Shapiro-Wilk W = 0.9656	0.6436	Normally distributed
		HFD+API	3	1.110 ± 0.0707Shapiro-Wilk W = 0.9653	0.6421	Normally distributed
		HFD+AE+API	3	1.234 ± 0.0512Shapiro-Wilk W = 0.8680	0.2898	Normally distributed
	**2. Homogeneity of variance test & one-way ANOVA**	Brown-Forsythe test	5, 12	F = 0.1565	0.97	Equal variances across groups;Welch/Dunnett's T3 not required
		One-way ANOVA (overall)	5, 12	F = 85.83	< 0.001	Significant difference among 6 groups
	**3. Tukey's post-hoc multiple comparisons**	CON vs. HFD	-	Mean difference = 0.6040	< 0.001	***
		HFD vs. SO	-	Mean difference = −0.0225	> 0.99	ns
		HFD vs. HFD+AE	-	Mean difference = −0.4671	< 0.001	***
		HFD vs. HFD+API	-	Mean difference = −0.4892	< 0.001	***
		HFD vs. HFD+AE+API	-	Mean difference = −0.6128	< 0.001	***
		HFD+AE vs. HFD+AE+API	-	Mean difference = −0.1275	0.10	ns

Indicator	Module	Group/Test item	n/DF	Value (Mean ± SD / Statistic)	P value	Conclusion / Significance
		HFD+API vs. HFD+AE+API	-	Mean difference = −0.1236	0.11	ns
**KIM-1**	**1. Descriptive statistics & Shapiro-Wilk normality test**	CON	3	0.6190 ± 0.0824Shapiro-Wilk W = 0.8162	0.1537	Normally distributed
		HFD	3	1.241 ± 0.0417Shapiro-Wilk W = 0.8738	0.3063	Normally distributed
		SO	3	1.124 ± 0.0644Shapiro-Wilk W = 0.9093	0.4159	Normally distributed
		HFD+AE	3	0.8682 ± 0.0327Shapiro-Wilk W = 0.8428	0.2213	Normally distributed
		HFD+API	3	0.8685 ± 0.0140Shapiro-Wilk W = 0.9547	0.5903	Normally distributed
		HFD+AE+API	3	0.6587 ± 0.0815Shapiro-Wilk W = 0.9731	0.6852	Normally distributed
	**2. Homogeneity of variance test & one-way ANOVA**	Brown-Forsythe test	5, 12	F = 0.4386	0.81	Equal variances across groups;Welch/Dunnett's T3 not required
		One-way ANOVA (overall)	5, 12	F = 53.52	< 0.001	Significant difference among 6 groups
	**3. Tukey's post-hoc multiple**	CON vs. HFD	-	Mean difference = −0.6222	< 0.001	***

Indicator	Module	Group/Test item	n/DF	Value (Mean ± SD / Statistic)	P value	Conclusion / Significance
	**comparisons**					
		HFD vs. SO	-	Mean difference = 0.1168	0.22	ns
		HFD vs. HFD+AE	-	Mean difference = 0.3730	< 0.001	***
		HFD vs. HFD+API	-	Mean difference = 0.3726	< 0.001	***
		HFD vs. HFD+AE+API	-	Mean difference = 0.5824	< 0.001	***
		HFD+AE vs. HFD+AE+API	-	Mean difference = 0.2095	0.009	**
		HFD+API vs. HFD+AE+API	-	Mean difference = 0.2098	0.009	**
**NGAL**	**1. Descriptive statistics & Shapiro-Wilk normality test**	CON	3	0.5048 ± 0.1276Shapiro-Wilk W = 0.8142	0.1490	Normally distributed
		HFD	3	0.8592 ± 0.0538Shapiro-Wilk W = 0.9625	0.6278	Normally distributed
		SO	3	0.7535 ± 0.0601Shapiro-Wilk W = 0.8219	0.1680	Normally distributed
		HFD+AE	3	0.5414 ± 0.0980Shapiro-Wilk W = 0.7822	0.0727	Normally distributed
		HFD+API	3	0.5731 ± 0.0969Shapiro-Wilk W = 0.8124	0.1446	Normally distributed

Indicator	Module	Group/Test item	n/DF	Value (Mean ± SD / Statistic)	P value	Conclusion / Significance
		HFD+AE+API	3	0.5257 ± 0.0840Shapiro-Wilk W = 0.8576	0.2611	Normally distributed
	**2. Homogeneity of variance test & one-way ANOVA**	Brown-Forsythe test	5, 12	F = 0.1108	0.99	Equal variances across groups;Welch/Dunnett's T3 not required
		One-way ANOVA (overall)	5, 12	F = 7.767	0.002	Significant difference among 6 groups
	**3. Tukey's post-hoc multiple comparisons**	CON vs. HFD	-	Mean difference = −0.3544	0.004	**
		HFD vs. SO	-	Mean difference = 0.1058	0.71	ns
		HFD vs. HFD+AE	-	Mean difference = 0.3178	0.010	**
		HFD vs. HFD+API	-	Mean difference = 0.2861	0.02	*
		HFD vs. HFD+AE+API	-	Mean difference = 0.3335	0.007	**
		HFD+AE vs. HFD+AE+API	-	Mean difference = 0.0157	> 0.99	ns
		HFD+API vs. HFD+AE+API	-	Mean difference = 0.0474	0.98	ns
**L-FABP**	**1. Descriptive statistics & Shapiro-Wilk normality test**	CON	3	0.6456 ± 0.0642Shapiro-Wilk W = 0.8497	0.2396	Normally distributed
		HFD	3	1.017 ± 0.0936Shapiro-Wilk W = 0.9904	0.8130	Normally distributed

Indicator	Module	Group/Test item	n/DF	Value (Mean ± SD / Statistic)	P value	Conclusion / Significance
		SO	3	1.013 ± 0.0608Shapiro-Wilk W = 0.9878	0.7884	Normally distributed
		HFD+AE	3	0.7147 ± 0.0205Shapiro-Wilk W = 0.7779	0.0627	Normally distributed
		HFD+API	3	0.6946 ± 0.0179Shapiro-Wilk W = 0.9558	0.5954	Normally distributed
		HFD+AE+API	3	0.6048 ± 0.0488Shapiro-Wilk W = 0.8273	0.1815	Normally distributed
	**2. Homogeneity of variance test & one-way ANOVA**	Brown-Forsythe test	5, 12	F = 0.6675	0.66	Equal variances across groups;Welch/Dunnett's T3 not required
		One-way ANOVA (overall)	5, 12	F = 31.20	< 0.001	Significant difference among 6 groups
	**3. Tukey's post-hoc multiple comparisons**	CON vs. HFD	-	Mean difference = −0.3710	< 0.001	***
		HFD vs. SO	-	Mean difference = 0.0032	> 0.99	ns
		HFD vs. HFD+AE	-	Mean difference = 0.3020	< 0.001	***
		HFD vs. HFD+API	-	Mean difference = 0.3220	< 0.001	***
		HFD vs. HFD+AE+API	-	Mean difference = 0.4119	< 0.001	***

Indicator	Module	Group/Test item	n/DF	Value (Mean ± SD / Statistic)	P value	Conclusion / Significance
		HFD+AE vs. HFD+AE+API	-	Mean difference = 0.1099	0.25	ns
		HFD+API vs. HFD+AE+API	-	Mean difference = 0.0898	0.44	ns
**SOD**	**1. Descriptive statistics & Shapiro-Wilk normality test**	CON	3	0.9042 ± 0.0306Shapiro-Wilk W = 0.9808	0.7346	Normally distributed
		HFD	3	0.3576 ± 0.0250Shapiro-Wilk W = 0.8621	0.2734	Normally distributed
		SO	3	0.3993 ± 0.0600Shapiro-Wilk W = 0.7725	0.0505	Normally distributed
		HFD+AE	3	0.7536 ± 0.1213Shapiro-Wilk W = 0.9477	0.5592	Normally distributed
		HFD+API	3	0.8426 ± 0.0693Shapiro-Wilk W = 0.8255	0.1769	Normally distributed
		HFD+AE+API	3	0.9993 ± 0.0509Shapiro-Wilk W = 0.8752	0.3105	Normally distributed
	**2. Homogeneity of variance test & one-way ANOVA**	Brown-Forsythe test	5, 12	F = 0.5525	0.73	Equal variances across groups;Welch/Dunnett's T3 not required
		One-way ANOVA (overall)	5, 12	F = 51.12	< 0.001	Significant difference among 6 groups

Indicator	Module	Group/Test item	n/DF	Value (Mean ± SD / Statistic)	P value	Conclusion / Significance
	**3. Tukey's post-hoc multiple comparisons**	CON vs. HFD	-	Mean difference = 0.5466	< 0.001	***
		HFD vs. SO	-	Mean difference = −0.0417	0.97	ns
		HFD vs. HFD+AE	-	Mean difference = −0.3960	< 0.001	***
		HFD vs. HFD+API	-	Mean difference = −0.4850	< 0.001	***
		HFD vs. HFD+AE+API	-	Mean difference = −0.6751	< 0.001	***
		HFD+AE vs. HFD+AE+API	-	Mean difference = −0.2791	0.003	**
		HFD+API vs. HFD+AE+API	-	Mean difference = −0.1901	0.04	*
