# Supplementary material for: Apigenin Combined with Aerobic Exercise Is Associated with Attenuated Renal Oxidative Stress and AMPK/SIRT1/PGC-1α Pathway Activation in NAFLD Mice
Source: Curr Issues Mol Biol. 2026 Sep 15;48(9):942. doi: 10.3390/cimb48090942 (PMC13605681; doi:10.3390/cimb48090942)
Supplement: Supplementary file 1 [file cimb-48-00942-s001.zip › Supplementary Files/Supplementary Tables/Supplementary Table S4. Complete statistical analysis of all immunohistochemical indicators and mesangial matrix area fraction measured by PAS staining.pdf]

Indicator	Module	Group/Test item	n/DF	Value (Mean ± SD / Statistic)	P value	Conclusion / Significance
**4-HNE**	**1.Descriptive statistics & Shapiro-Wilk normality test**	CON	3	1.000 ± 0.000; Shapiro-Wilk W = Invalid input data	—	—
		HFD	3	5.608 ± 0.9284; Shapiro-Wilk W = 0.7891	0.0888	Normally distributed
		SO	3	5.875 ± 0.5040; Shapiro-Wilk W = 0.9331	0.5004	Normally distributed
		HFD+AE	3	3.745 ± 0.1815; Shapiro-Wilk W = 0.9715	0.6759	Normally distributed
		HFD+API	3	3.645 ± 0.3372; Shapiro-Wilk W = 0.9952	0.8679	Normally distributed
		HFD+AE+API	3	1.378 ± 0.0929; Shapiro-Wilk W = 0.8728	0.3034	Normally distributed
	**2.Homogeneity of variance test & one-way ANOVA**	Brown-Forsythe test	5, 12	F = 0.7655	0.59	Equal variances across groups; Welch/Dunnett's T3 not required
		One-way ANOVA (overall)	5, 12	F = 59.22	< 0.001	Significant difference among 6 groups
	**3.Tukey's post-hoc multiple comparisons**	CON vs. HFD		Mean difference = −4.608	< 0.001	***
		HFD vs. SO		Mean difference = −0.2671	0.98	ns
		HFD vs. HFD+AE		Mean difference = 1.863	0.003	**
		HFD vs. HFD+API		Mean difference = 1.963	0.002	**
		HFD vs. HFD+AE+API		Mean difference = 4.230	< 0.001	***

Indicator	Module	Group/Test item	n/DF	Value (Mean ± SD / Statistic)	P value	Conclusion / Significance
		HFD+AE vs. HFD+AE+API		Mean difference = 2.367	< 0.001	***
		HFD+API vs. HFD+AE+API		Mean difference = 2.268	< 0.001	***
**KIM-1**	**1.Descriptive statistics & Shapiro-Wilk normality test**	CON	3	1.000 ± 0.000; Shapiro-Wilk W = Invalid input data	—	—
		HFD	3	5.433 ± 0.4887; Shapiro-Wilk W = 0.7865	0.0826	Normally distributed
		SO	3	5.317 ± 0.2447; Shapiro-Wilk W = 0.9984	0.9231	Normally distributed
		HFD+AE	3	3.163 ± 0.2247; Shapiro-Wilk W = 0.7792	0.0659	Normally distributed
		HFD+API	3	3.302 ± 0.2333; Shapiro-Wilk W = 0.9303	0.4895	Normally distributed
		HFD+AE+API	3	2.116 ± 0.1199; Shapiro-Wilk W = 0.9327	0.4988	Normally distributed
	**2.Homogeneity of variance test & one-way ANOVA**	Brown-Forsythe test	5, 12	F = 0.5238	0.75	Equal variances across groups; Welch/Dunnett's T3 not required
		One-way ANOVA (overall)	5, 12	F = 131.6	< 0.001	Significant difference among 6 groups
	**3.Tukey's post-hoc multiple comparisons**	CON vs. HFD		Mean difference = −4.433	< 0.001	***
		HFD vs. SO		Mean difference = 0.1159	> 0.99	ns
		HFD vs. HFD+AE		Mean difference = 2.270	< 0.001	***

Indicator	Module	Group/Test item	n/DF	Value (Mean ± SD / Statistic)	P value	Conclusion / Significance
		HFD vs. HFD+API		Mean difference = 2.131	< 0.001	***
		HFD vs. HFD+AE+API		Mean difference = 3.317	< 0.001	***
		HFD+AE vs. HFD+AE+API		Mean difference = 1.047	0.004	**
		HFD+API vs. HFD+AE+API		Mean difference = 1.186	0.001	**
**NGAL**	**1.Descriptive statistics & Shapiro-Wilk normality test**	CON	3	1.000 ± 0.000; Shapiro-Wilk W = Invalid input data	—	—
		HFD	3	5.970 ± 0.5677; Shapiro-Wilk W = 0.9854	0.7689	Normally distributed
		SO	3	5.292 ± 0.4428; Shapiro-Wilk W = 0.9530	0.5827	Normally distributed
		HFD+AE	3	2.223 ± 0.1456; Shapiro-Wilk W = 0.9977	0.9091	Normally distributed
		HFD+API	3	2.208 ± 0.2317; Shapiro-Wilk W = 0.9503	0.5707	Normally distributed
		HFD+AE+API	3	1.579 ± 0.1248; Shapiro-Wilk W = 0.8119	0.1432	Normally distributed
	**2.Homogeneity of variance test & one-way ANOVA**	Brown-Forsythe test	5, 12	F = 1.408	0.29	Equal variances across groups; Welch/Dunnett's T3 not required
		One-way ANOVA (overall)	5, 12	F = 126.0	< 0.001	Significant difference among 6 groups
	**3.Tukey's post-hoc multiple comparisons**	CON vs. HFD		Mean difference = −4.970	< 0.001	***

Indicator	Module	Group/Test item	n/DF	Value (Mean ± SD / Statistic)	P value	Conclusion / Significance
		HFD vs. SO		Mean difference = 0.6777	0.17	ns
		HFD vs. HFD+AE		Mean difference = 3.746	< 0.001	***
		HFD vs. HFD+API		Mean difference = 3.761	< 0.001	***
		HFD vs. HFD+AE+API		Mean difference = 4.391	< 0.001	***
		HFD+AE vs. HFD+AE+API		Mean difference = 0.6442	0.21	ns
		HFD+API vs. HFD+AE+API		Mean difference = 0.6292	0.22	ns
**L-FABP**	**1.Descriptive statistics & Shapiro-Wilk normality test**	CON	3	1.000 ± 0.000; Shapiro-Wilk W = Invalid input data	—	—
		HFD	3	6.704 ± 0.8770; Shapiro-Wilk W = 0.8966	0.3747	Normally distributed
		SO	3	6.194 ± 0.7004; Shapiro-Wilk W = 0.9871	0.7824	Normally distributed
		HFD+AE	3	3.086 ± 0.1752; Shapiro-Wilk W = 0.9543	0.5887	Normally distributed
		HFD+API	3	2.608 ± 0.2137; Shapiro-Wilk W = 0.8344	0.1995	Normally distributed
		HFD+AE+API	3	1.749 ± 0.1682; Shapiro-Wilk W = 0.9913	0.8213	Normally distributed
	**2.Homogeneity of variance test & one-way ANOVA**	Brown-Forsythe test	5, 12	F = 1.269	0.34	Equal variances across groups; Welch/Dunnett's T3 not required

Indicator	Module	Group/Test item	n/DF	Value (Mean ± SD / Statistic)	P value	Conclusion / Significance
		One-way ANOVA (overall)	5, 12	F = 73.32	< 0.001	Significant difference among 6 groups
	**3.Tukey's post-hoc multiple comparisons**	CON vs. HFD		Mean difference = −5.704	< 0.001	***
		HFD vs. SO		Mean difference = 0.5093	0.78	ns
		HFD vs. HFD+AE		Mean difference = 3.618	< 0.001	***
		HFD vs. HFD+API		Mean difference = 4.095	< 0.001	***
		HFD vs. HFD+AE+API		Mean difference = 4.954	< 0.001	***
		HFD+AE vs. HFD+AE+API		Mean difference = 1.337	0.04	*
		HFD+API vs. HFD+AE+API		Mean difference = 0.8590	0.30	ns
**p-AMPK/AMPK**	**1.Descriptive statistics & Shapiro-Wilk normality test**	CON	3	3.421 ± 0.2474; Shapiro-Wilk W = 0.8198	0.1627	Normally distributed
		HFD	3	0.4128 ± 0.04760; Shapiro-Wilk W = 0.7507	0.0015	Not normally distributed
		SO	3	0.5213 ± 0.07472; Shapiro-Wilk W = 0.7753	0.0567	Normally distributed
		HFD+AE	3	1.536 ± 0.07137; Shapiro-Wilk W = 0.9647	0.6390	Normally distributed
		HFD+API	3	1.461 ± 0.5213; Shapiro-Wilk W = 0.8654	0.2825	Normally distributed
		HFD+AE+API	3	3.419 ± 0.1385;	0.0281	Not normally distributed

Indicator	Module	Group/Test item	n/DF	Value (Mean ± SD / Statistic)	P value	Conclusion / Significance
				Shapiro-Wilk W = 0.7627		
	**2.Homogeneity of variance test & one-way ANOVA**	Brown-Forsythe test	5, 12	F = 0.8531	0.54	Equal variances across groups; Welch/Dunnett's T3 not required
		One-way ANOVA (overall)	5, 12	F = 88.67	< 0.001	Significant difference among 6 groups
	**3.Tukey's post-hoc multiple comparisons**	CON vs. HFD		Mean difference = 3.008	< 0.001	***
		HFD vs. SO		Mean difference = −0.1086	> 0.99	ns
		HFD vs. HFD+AE		Mean difference = −1.123	0.001	**
		HFD vs. HFD+API		Mean difference = −1.049	0.002	**
		HFD vs. HFD+AE+API		Mean difference = −3.006	< 0.001	***
		HFD+AE vs. HFD+AE+API		Mean difference = −1.883	< 0.001	***
		HFD+API vs. HFD+AE+API		Mean difference = −1.957	< 0.001	***
**SIRT1**	**1.Descriptive statistics & Shapiro-Wilk normality test**	CON	3	1.000 ± 0.000; Shapiro-Wilk W = Invalid input data	—	—
		HFD	3	0.3938 ± 0.03292; Shapiro-Wilk W = 0.9896	0.8050	Normally distributed
		SO	3	0.3627 ± 0.08854; Shapiro-Wilk W = 0.8366	0.2052	Normally distributed
		HFD+AE	3	0.6054 ± 0.05783; Shapiro-Wilk W =	0.2559	Normally distributed

Indicator	Module	Group/Test item	n/DF	Value (Mean ± SD / Statistic)	P value	Conclusion / Significance
				0.8557		
		HFD+API	3	0.6234 ± 0.04137; Shapiro-Wilk W = 0.9541	0.5876	Normally distributed
		HFD+AE+API	3	0.9180 ± 0.03735; Shapiro-Wilk W = 0.7735	0.0527	Normally distributed
	**2.Homogeneity of variance test & one-way ANOVA**	Brown-Forsythe test	5, 12	F = 0.5229	0.75	Equal variances across groups; Welch/Dunnett's T3 not required
		One-way ANOVA (overall)	5, 12	F = 80.83	< 0.001	Significant difference among 6 groups
	**3.Tukey's post-hoc multiple comparisons**	CON vs. HFD		Mean difference = 0.6062	< 0.001	***
		HFD vs. SO		Mean difference = 0.03109	0.97	ns
		HFD vs. HFD+AE		Mean difference = −0.2116	0.003	**
		HFD vs. HFD+API		Mean difference = −0.2296	0.001	**
		HFD vs. HFD+AE+API		Mean difference = −0.5242	< 0.001	***
		HFD+AE vs. HFD+AE+API		Mean difference = −0.3126	< 0.001	***
		HFD+API vs. HFD+AE+API		Mean difference = −0.2946	< 0.001	***
**PGC-1α**	**1.Descriptive statistics & Shapiro-Wilk normality test**	CON	3	1.000 ± 0.000; Shapiro-Wilk W = Invalid input data	—	—
		HFD	3	0.2238 ± 0.03371; Shapiro-Wilk W = 0.9233	0.4640	Normally distributed

**Indicator**	**Module**	**Group/Test item**	**n/DF**	**Value (Mean ± SD / Statistic)**	**P value**	**Conclusion / Significance**
		SO	3	0.2564 ± 0.01792; Shapiro-Wilk W = 0.9220	0.4593	Normally distributed
		HFD+AE	3	0.6259 ± 0.04258; Shapiro-Wilk W = 0.9969	0.8944	Normally distributed
		HFD+API	3	0.6337 ± 0.05245; Shapiro-Wilk W = 0.8715	0.2999	Normally distributed
		HFD+AE+API	3	0.8489 ± 0.08019; Shapiro-Wilk W = 0.7717	0.0485	Not normally distributed
	**2.Homogeneity of variance test & one-way ANOVA**	Brown-Forsythe test	5, 12	F = 0.5244	0.75	Equal variances across groups; Welch/Dunnett's T3 not required
		One-way ANOVA (overall)	5, 12	F = 139.7	< 0.001	Significant difference among 6 groups
	**3.Tukey's post-hoc multiple comparisons**	CON vs. HFD		Mean difference = 0.7762	< 0.001	***
		HFD vs. SO		Mean difference = −0.03260	0.95	ns
		HFD vs. HFD+AE		Mean difference = −0.4021	< 0.001	***
		HFD vs. HFD+API		Mean difference = −0.4099	< 0.001	***
		HFD vs. HFD+AE+API		Mean difference = −0.6251	< 0.001	***
		HFD+AE vs. HFD+AE+API		Mean difference = −0.2230	< 0.001	***
		HFD+API vs. HFD+AE+API		Mean difference = −0.2152	< 0.001	***
**Mesangial**	**1.Descriptive statistics**	CON	3	20.09 ± 0.6939;	0.1902	Normally distributed

Indicator	Module	Group/Test item	n/DF	Value (Mean ± SD / Statistic)	P value	Conclusion / Significance
**matrix area fraction**	**& Shapiro-Wilk normality test**			Shapiro-Wilk W = 0.8307		
		HFD	3	68.06 ± 2.014; Shapiro-Wilk W = 0.7995	0.1134	Normally distributed
		SO	3	72.60 ± 5.703; Shapiro-Wilk W = 0.9849	0.7649	Normally distributed
		HFD+AE	3	58.00 ± 1.339; Shapiro-Wilk W = 0.9023	0.3928	Normally distributed
		HFD+API	3	53.98 ± 2.576; Shapiro-Wilk W = 0.9584	0.6079	Normally distributed
		HFD+AE+API	3	44.83 ± 4.618; Shapiro-Wilk W = 0.8915	0.3591	Normally distributed
	**2.Homogeneity of variance test & one-way ANOVA**	Brown-Forsythe test	5, 12	F = 0.8963	0.51	Equal variances across groups; Welch/Dunnett's T3 not required
		One-way ANOVA (overall)	5, 12	F = 96.30	< 0.001	Significant difference among 6 groups
	**3.Tukey's post-hoc multiple comparisons**	CON vs. HFD		Mean difference = −47.98	< 0.001	***
		HFD vs. SO		Mean difference = −4.540	0.58	ns
		HFD vs. HFD+AE		Mean difference = 10.06	0.03	*
		HFD vs. HFD+API		Mean difference = 14.08	0.002	**
		HFD vs. HFD+AE+API		Mean difference = 23.24	< 0.001	***

Indicator	Module	Group/Test item	n/DF	Value (Mean ± SD / Statistic)	P value	Conclusion / Significance
		HFD+AE vs. HFD+AE+API		Mean difference = 13.17	0.004	**
		HFD+API vs. HFD+AE+API		Mean difference = 9.157	0.05	*
